# Investigation of Potential Amorphisation and Co-Amorphisation Behaviour of the Benzene Di-Carboxylic Acids upon Cryo-Milling

**DOI:** 10.3390/molecules24213990

**Published:** 2019-11-05

**Authors:** Rehab Elfakhri, Jonathan C. Burley

**Affiliations:** School of Pharmacy, University of Nottingham, Nottingham NG7 2RD, UK; rehab.elfakhri@uob.edu.ly

**Keywords:** formulation, cryo-milling, amorphous, co-amorphous, XRPD, DSC, ATR-FTIR, mechanochemistry

## Abstract

Multi-component formulations offer a way to modulate the physico-chemical properties of drug molecules and thereby enhance their efficacy as medicines compared to using only the raw drug, with mechano-chemical synthesis being an increasingly popular way to create these novel materials in a research setting. However, to date studies have focussed on employing pharmaceutically acceptable components, which has led to the literature featuring chemically diverse pairings of drug and excipient. Here we investigate the outcome of cryo-milling and co-cryo-milling of a series of three simple geometrical isomers of benzene di-carboxylic acid with a view to developing a chemically simple model system to investigate areas including cryo-milling, co-cryo-milling, co-amorphous formulation, etc. All three single-component materials exhibit differing behaviour upon cryo-milling and subsequent storage, as do the two-component mixtures. The surprisingly differing behaviours of these chemically similar species upon cryo-milling and co-cryo-milling suggest that molecular chemistry may not be the dominant influence on the outcome of mechano-chemical syntheses, and that other properties should be explored to develop a predictive model for the outcomes of these types of reactions.

## 1. Introduction

Many drug molecules for oral delivery exhibit poor gastro-intestinal (GI) solubility and/or dissolution rates which render them unsuitable for use in medicines [1]. This seems to be a growing issue in recent years and can be addressed using strategies designed to create super-saturation of the drug in the intestine [2]. Typically, this is achieved by creating a new multi-component single-phase drug form. The most popular strategy employed here is salt formation [3,4,5], but other strategies such as formation of co-crystals [6,7,8,9,10], amorphous solid dispersions (ASDs) in which the drug is molecularly dispersed into a polymer matrix [11,12,13,14], lipid formulations [15,16,17,18], etc. are also options. Changing the solid form of the drug impacts a range of properties beyond simply the maximum concentration of drug which can be achieved in the GI tract and formulation is therefore undertaken on a case-by-case basis.

An emerging area of formulation is the creation of so-called co-amorphous systems (see [2,19,20] and references therein). These are similar to ASDs in that they are two-component, single-phase amorphous materials. They are distinct from ASDs in that co-amorphous formulations comprise two small molecules (either drug-drug or drug-coformer), rather than a small molecule and polymer as happens with ASDs. The co-amorphous approach is essentially another way of stabilising the drug in its amorphous form and thereby hopefully boosting the maximum concentration of the drug in the GI tract compared to the as-synthesised pure drug (which is usually crystalline). The use of small molecules having low molecular weights compared to polymers allows for a potentially higher drug loading of the formulation. It is interesting to note that co-amorphous formulation can allow access to amorphous forms of drugs which are not stable in the amorphous state by themselves, similar to the use of ASDs. For example, naproxen could not be made amorphous by melt-quenching on its own but could when mixed with indomethacin [21].

While co-amorphous systems are potentially attractive formulation options, a reliable predictive model for formation of co-amorphous materials does not yet exist. The importance of potential salt formation has been emphasised, with titration-type experiments indicating that the co-amorphous salt is a distinct chemical entity compared to the drug and co-former [22]. However, beyond salt formation there exist no clear guidelines on how to best select a co-former for a drug to produce a co-amorphous formulation. To date work performed in this area has mainly targeted co-formers based upon a consideration of their biocompatibility, with amino acids being well studied for example [22,23,24]. There do not appear to be any studies investigating the potential formation of co-amorphous systems where structural features are used to guide selection of the co-former. Such an approach might be expected to shed some light on any structural requirements or guidelines for co-amorphous formulation.

**Figure 1:**
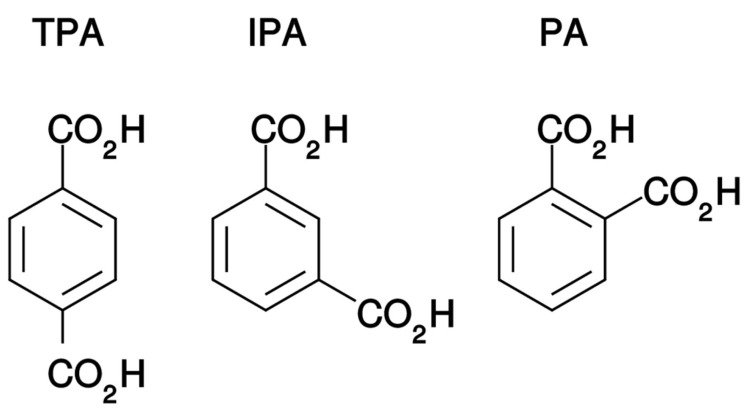
Chemical structures of terephthalic acid (TPA), isophthalic acid (IPA) and phthalic acid (PA).

With this in mind and the usual preference for simple model systems where possible, we therefore investigate a series of geometric isomers of the same molecule in this paper with a view to probing any relationship between their chemical structure and propensity to both amorphisation and co-amorphisation. The three compounds selected are the benzene di-carboxylic acids: terephthalic acid (TPA: benzene-1,4-dicarboxylic acid); isophthalic acid (IPA: benzene-1,3-dicarboxylic acid); and phthalic acid (PA: benzene-1,2-dicarboxylic acid). These compounds are shown in Figure 1, and were selected for several reasons: (i) their safety profile is good; (ii) they exhibit very low aqueous solubilities [25,26,27] so are reasonable models for the “brick dust” pharmaceutical compounds; (iii) they are readily available and well characterised (for example TPA is a commodity chemical produced on a scale of several million tonnes a year [28]); (iv) they are not likely to form salts when mixed which simplifies matters; (v) they contain functional groups (phenyl ring and carboxylic acids) commonly found in pharmaceuticals. None of the three compounds had previously been reported to adopt anything other than crystalline forms [29,30,31]. As such there are no reports of glass transition temperatures for any of these three di-carboxylic acids.

Various methods of combining materials to make co-amorphous systems have been employed in the literature, including melt-quenching [21,32], solvent evaporation [33], milling [34] and cryo-milling [35]. Of these the milling and cryo-milling methods are the most obviously suitable for TPA, IPA and PA due to their very low solubilities.

We therefore report below the results obtained by cryo-milling TPA, IPA and PA separately and in pair-wise combination. Analytical methods employed include X-ray powder diffraction (XRPD), Attenuated Total Reflectance Infra-Red (ATR-FTIR) spectroscopy, and Differential Scanning Calorimetry (DSC).

## 2. Results

The results of cryo-milling the separate components are reported first, followed by the results for co-cryo-milling. The two questions being addressed are: (i) what changes, if any, occur upon cryo-milling; (ii) is co-cryo-milling equivalent to separate cryo-milling or is there evidence for some synergy when co-cryo-milling is performed?

### 2.1. Single Component Samples

#### 2.1.1. DSC Experiments

**Figure 2:**
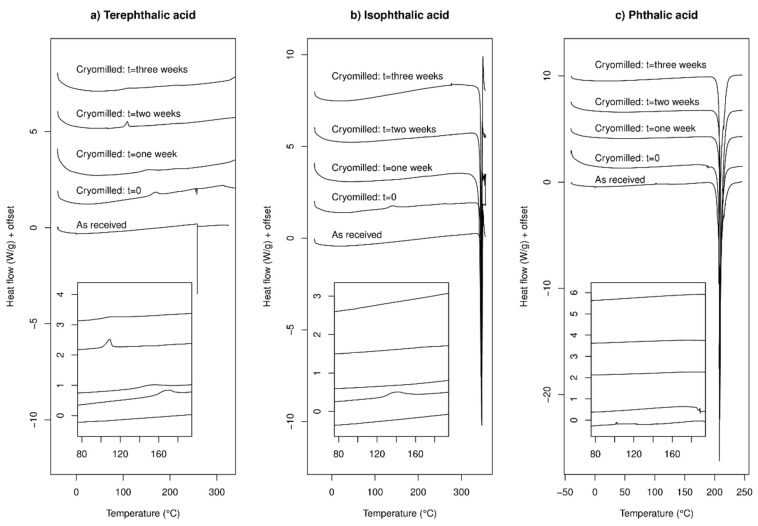
Differential Scanning Calorimetry (DSC) data for: (**a**) TPA; (**b**) IPA; and (**c**) PA. Exothermic up.

DSC data for TPA before and after cryo-milling are presented in Figure 2a. The reported experimental melting and sublimation temperature of TPA (402 °C, [36]) is well above the temperature range studied in this experiment. The as-received sample exhibits a DSC trace in full accord with that expected, i.e., nothing of note happens in the temperature range studied (the small downward spike is an instrumental artefact). The cryo-milled samples however exhibit DSC traces which are subtly but noticeably different to the as-received sample. For a sample studied immediately after cryo-milling, a small exothermic peak is visible around 160–170 °C. As the sample is aged for one and two weeks the small exothermic peak becomes more prominent and moves to lower temperatures, reaching ca. 100 °C after two weeks of storage at 43% realtive humidity (RH). After three weeks the peak is not readily apparent, but there may be a small broad feature around 100 °C. This exothermic peak in the cryo-milled samples is assigned as a recrystallisation, and this is taken to indicate that cryo-milling has disrupted the crystallinity of the original sample, either by converting some of the initial crystalline material into the amorphous state, or by introducing defects into the crystal lattice. The lowering of the recrystallisation peak temperature as a function of storage time may be due to the sample absorbing moisture, which is known well to increase the ease of recrystallisation [37]. The lack of a clear observable glass transition T_g_ in any of the traces in Figure 2a is not a clear diagnostic test for the presence of amorphous material, as the T_g_ could (for example) lie under the recrystallisation peak, be lower in magnitude than the limit of detection (due to only a small amount of amorphous material in the sample) or indeed be below the temperature range covered in these experiments.

DSC data for IPA are presented in Figure 2b. All samples exhibit a clear and large endothermic peak around 345 °C, which is taken to be the melting of the material, in accordance with the materials safety data sheet provided by the manufacturer (341–343 °C). Immediately after cryo-milling, a small exothermic peak appears at around 125 °C which is taken to indicate recrystallisation (as with TPA). This suggests that at least part of the sample has become significantly less crystalline due to the cryo-milling. However, for the samples measured one, two and three weeks after cryo-milling this peak is no longer detectable and the DSC traces strongly resemble that from the starting material. This observation is taken to indicate that amorphous (or crystal-defective) material produced by cryo-milling IPA rapidly recrystallises upon storage. This rapid reversion to an apparently fully crystalline material is in marked contrast to the behaviour of terephthalic acid noted earlier, which still exhibited a clear recrystallisation peak after two weeks of storage.

DSC data for PA are given in Figure 2c, the reported melting point of PA is 208 °C [38]. Data for the as-received sample show only a single endothermic event around 200–205 °C, and in line with literature this is ascribed to the sample melting. Upon first inspection cryo-milling does not appear to have a notable effect on the DSC trace, but with closer inspection (see inset) a thermal event is noted at around 189 °C in the sample measured immediately after cryo-milling, which is tentatively ascribed to a glass transition. This suggests the presence of some amorphous material, which was not present before cryo-milling and is not apparent in the samples measured after one, two and three weeks. If this thermal event is indeed a glass transition, cryo-milling has caused some of the PA sample to become amorphous. Because this feature is not detectable for later time-points this possible amorphous component is not stable upon storage.

To the best of our knowledge there are no previous reports of any amorphous samples of these di-carboxylic acids nor any T_g_ values for them. This makes it difficult to draw firm conclusions about any amorphisation of the samples due to cryo-milling from DSC data alone, beyond stating that TPA and IPA present some clear evidence for cryo-milling leading to some loss of crystallinity, for PA the situation is less clear. We therefore undertook some XRPD studies of these samples to further investigate the outcome of cryo-milling.

#### 2.1.2. XRPD Experiments

**Figure 3:**
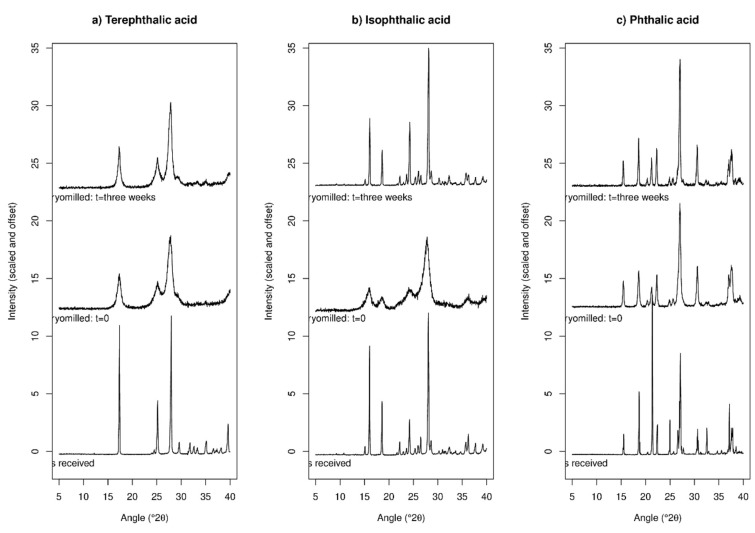
X-ray powder diffraction (XRPD) data for: (**a**) TPA; (**b**) IPA; and (**c**) PA, before and after cryo-milling and after storage at 43% relative humidity (RH) and room temperature for three weeks.

XRPD data for TPA before and after cryo-milling, and after three weeks storage, are given in Figure 3a. The as-received material exhibits multiple sharp Bragg peaks, indicative of high crystallinity, which is expected. Upon cryo-milling, the Bragg peaks remain present but are greatly broadened. After three weeks storage the peaks appear slightly sharper than immediately after cryo-milling but are still notably broader than the crystalline as-received material. The peak broadening observed is a clear indication of reduction in crystallite size, and estimates using the Scherrer equation suggest a crystallite size of around 9 nm immediately after cryo-milling, with this increasing to 14 nm after three weeks of storage. The peak position of the most intense peak also shifts slightly, being at 17.37(1)° prior to milling and 17.31(1)° afterwards (Figure S1a). This suggests a slight expansion of the crystal lattice upon cryo-milling, concomitant with the reduction in crystallite size. This may be caused by the introduction of defects into the lattice by the cryo-milling. The apparent stability of TPA upon storage here correlates well with the DSC observations in which an exothermic peak was remained apparent in the data after three weeks storage at 43% RH.

XRPD data for IPA are given in Figure 3b. The data prior to and immediately after cryo-milling are similar to those for terephthalic acid, with the material exhibiting sharp Bragg peaks prior to cryo-milling compared to very broad peaks immediately afterwards (for example the peak width of the 16° peak exhibits a Full Width at Half Maximum (FWHM) of 0.14° prior to cryo-milling and 1.45° afterwards. However, after three weeks of storage the Bragg peaks are again very sharp (0.21° FWHM), approaching the pre-milling state. If the peak broadening upon cryo-milling is due to crystallite size reduction, the three-week ageing period has clearly led to a size increase of these small crystallites, presumably via Ostwald ripening. There are some small peak shifts upon cryo-milling similar to those observed for TPA (Figure S1b), the recovery of sharp peaks widths upon storage at 43% RH for three weeks also causes the peaks to return to approximately their pre-milled position.

XRPD data for PA are presented in Figure 3c. While there are apparent changes in the XRPD intensities for this sample upon cryo-milling, these are simply due to the presence of strong preferred orientation of the crystallites in the as-received sample—this effect arises from the crystallites having very regular shapes and it is not surprising that cryo-milling disrupts this. No peak positions change upon cryo-milling and the peak intensities change to match those predicted from the single crystal structure [31]. Peak broadening upon cryo-milling is relatively minor for this sample, which contrasts with both terephthalic and isophthalic acid. Peak widths for the peak at approximately 21.2° are 0.16° prior to cryo-milling, 0.42° immediately after cryo-milling, and 0.23° after three weeks of storage. This peak also seems to shift to slightly lower angle (higher d-spacing) upon cryo-milling and subsequent storage (Figure S1c), with the measured peak position being 21.30, 21.25 and 21.22° prior to cryo-milling, immediately after cryo-milling, and after three weeks of subsequent storage respectively. The peak shifts appear reasonable if defects are being introduced into the lattice, and/or if the inter-molecular bonding is being weakened upon cryo-milling.

Overall, cryo-milling leads to peak broadening in XRPD for all three samples in the order IPA > TPA >> PA. After three weeks of storage all samples showed a sharpening of XRPD peaks, albeit this did not lead to a return to the original pre-cryo-milling sharpness. The peaks for TPA remained notably broad even after the storage for three weeks at 43% RH, those for IPA sharpened up to almost their pre-cryo-milling width after storage. All samples exhibited a small peak shift to lower angles upon cryo-milling. The small peak shifts are likely due to the introduction of defects into the crystal lattice upon cryo-milling and subsequent lowering of lattice energy, with defective crystals expected to have lower density than non-defective crystals. The behaviour of the three materials upon storage mirrored the behaviour seen using DSC.

#### 2.1.3. ATR-FTIR Experiments

We note that assignments of IR peaks for all samples are available in the literature, see refs [39,40,41] for TPA, IPA and PA respectively.

**Figure 4:**
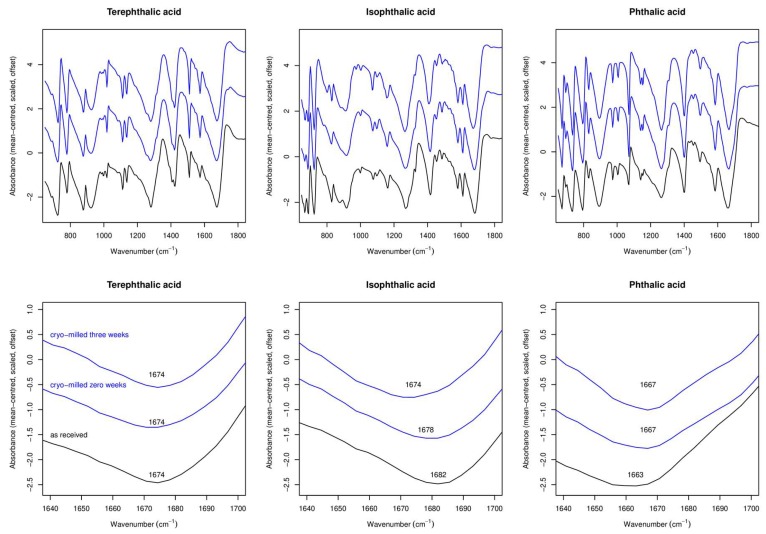
Attenuated Total Reflectance Infra-Red (ATR-FTIR) spectra for: TPA; IPA; and PA, as-received and after cryo-milling.

ATR-FTIR data for all single-component samples before cryo-milling, immediately after cryo-milling, and after three weeks of storage, are shown in Figure 4, along with expanded plots showing the C=O carbonyl peak.

For all samples the changes observed in the FTIR spectra are very small at most. There is no obvious consistency in the changes, with the carbonyl peak in TPA remaining unmoved upon cryo-milling, the same peak for IPA being slightly blue-shifted, and the same peak for PA being red-shifted. The limited changes observed in FTIR are in full accord with the molecules having relatively rigid structures and few rotatable bonds.

#### 2.1.4. Summary of Experimental Results for Single-Component Systems

Upon cryo-milling, XRPD and DSC data suggest that TPA is transformed into a mixed amorphous/nano-crystalline state (based upon a recrystallisation exotherm in DSC and very broad peaks in XRPD), the new material is reasonably stable for at least three weeks upon storage in 43% RH at room temperature. No changes are detected with ATR-FTIR upon cryo-milling. IPA likewise is transformed to a mixed amorphous/nano-crystalline state, but recrystallises within a week, as indicated by the sharpening of the XRPD peaks and the disappearance of the cryo-milling induced exotherm in DSC. The carbonyl peak is shifted slightly to lower wavenumber upon cryo-milling. PA in contrast does not appear to be rendered nano-crystalline and the evidence for any amorphous material is minimal (an ambiguous signal in DSC), the FTIR spectrum remains unchanged upon cryo-milling within limits of detection.

### 2.2. Multi-Component Samples

In the interests of brevity the data for the 1:1 molar mix two-component samples are discussed primarily in terms of whether or not there is any evidence that the behaviour upon cryo-milling is different to that of a simple 1:1 mix of the data for the two individual components, i.e., whether there is any evidence for a synergistic effect due to co-milling.

#### 2.2.1. DSC Experiments

We note again that the reported melting points of the components are: TPA 402 °C; IPA 341-343 °C; and PA 208 °C.

**Figure 5:**
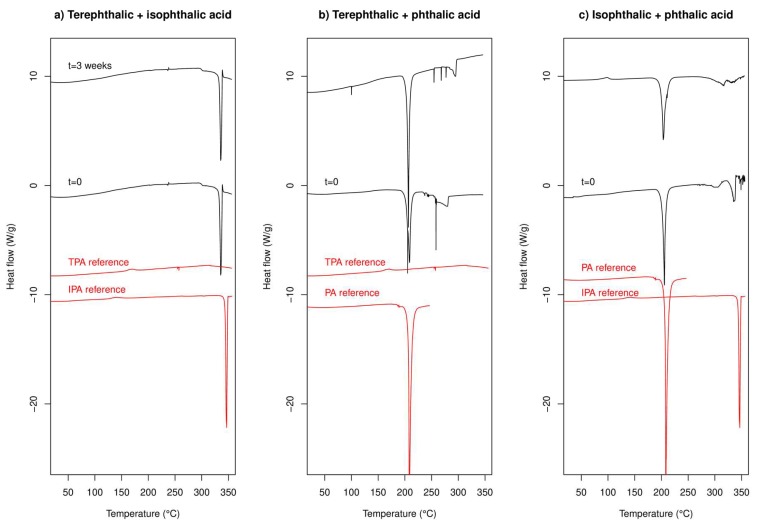
DSC data for co-cryo-milled combinations of (**a**) TPA, (**b**) IPA and (**c**) PA, with reference traces for the cryo-milled individual components at zero days also shown in red for reference. Exothermic up.

DSC data for co-cryo-milling TPA/IPA in a 1:1 molar ratio are presented in Figure 5a, where the DSC traces for the co-cryo-milled and separately cryo-milled samples are shown. TPA/IPA shows a strong endotherm around 330–340 °C, which is ascribed to the melting of the IPA component (pure melting point around 340 °C), with the melting of TPA being outside the temperature range probed in this experiment. There is a slight depression of the melting point of IPA in this mixture, any depression of the melting point of TPA is less than 50 °C (as our experiments stop 50 °C below the melting point of TPA). There is no obvious change in the traces after three weeks of aging.

TPA/PA shows a strong endotherm 200–205 °C and a weaker endotherm at around 280 °C. The first is ascribed to the melting of the PA component of the sample (pure melting point 208 °C). The second does not coincide with the reported melting point of TPA (it is at least 120 °C lower than expected) and seems too suppressed to be the usual colligative reduction of melting point. The enthalpy of fusion of TPA has been reported to be 40.425 kJ/mol [42] and a simple Schroder van Laar calculation suggests that a melting point depression of 120 °C would require a mole fraction of TPA of only 0.2. Not only does this seem unlikely when the actual mole fraction of TPA is 0.5, this mole fraction likely lies the far side of the eutectic point and is likely to be experimentally inaccessible. The observed thermal event in the TPA/PA system around 280 °C is therefore unexplained but may represent the melting point of a new material having been formed upon co-cryo-milling. We return to this point when discussing XRPD data. No obvious change in the DSC data is seen after three weeks of storage.

IPA/PA shows a strong endotherm at around 200 °C, ascribed to the melting of PA and a weaker one around 320 °C ascribed to the melting of IPA at a slightly depressed temperature compared to the pure material. There is little change in the traces after three weeks of aging.

No clear changes occur in the DSC traces on storing the samples for three weeks. The small exotherm which appears for the IPA/PA trace around 100 °C may be amorphous material recrystallising, but given the lack of anything similar in the t = 0 trace it may rather be instrumental.

Overall the DSC data indicate an anomaly for the TPA/PA system with a new endothermic event, likely a melt, which does not correspond with the reported melting points of either of the components and which seems to be difficult to explain using standard melting point depression theory, but data are otherwise generally unremarkable.

#### 2.2.2. XRPD Experiments

**Figure 6:**
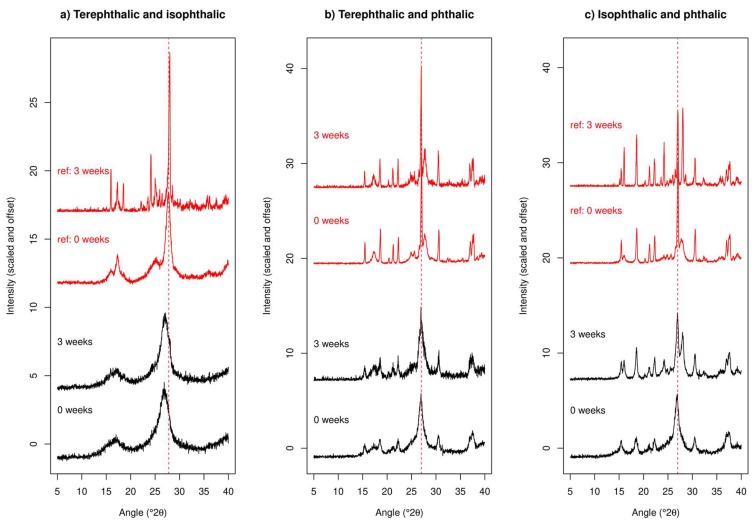
XRPD data for co-cryo-milled combinations of (**a**) TPA, (**b**) IPA and (**c**) PA as labelled. Red lines indicate the peak positions of the most intense peaks of the reference materials (freshly cryo-milled individual components) which are labelled “ref” and shown in red.

XRPD data for co-cryo-milling and separately cryo-milling TPA/IPA are shown in Figure 6a. It is immediately apparent from inspection of the data that the outcome of these two processes are markedly different. The co-cryo-milled samples exhibit both a notable peak shift to low angle (large d-spacing) and a significant broadening compared to the separately cryo-milled samples. Also, the co-cryo-milled XRPD traces do not change a great deal upon three weeks storage, whereas the separately cryo-milled samples exhibit clear peak sharpening upon storage. This peak sharpening is interpreted as an increase in crystallite size due to Ostwald ripening.

**Figure 7:**
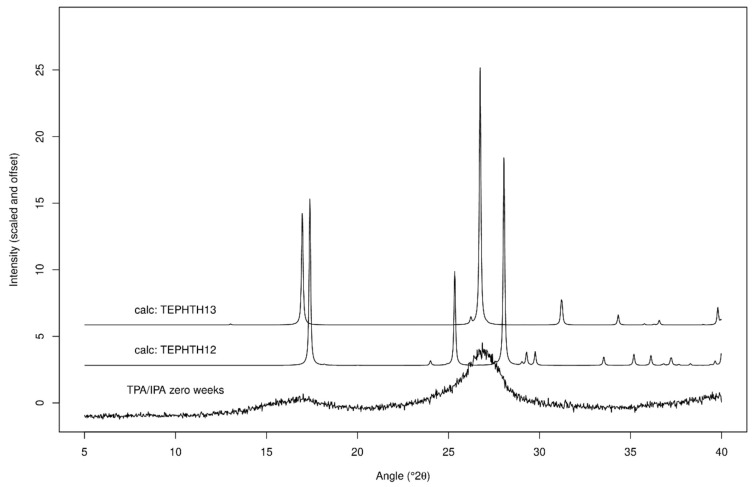
XRPD data for co-cryo-milled TPA/IPA, along with the predicted XRPD traces for the various TPA polymorphs.

Careful inspection of these new and unexpected traces for the co-cryo-milled TPA/IPA materials indicates that the new peak positions coincide almost exactly with the positions expected for a previously reported polymorph of TPA, the C2/c variant rather than the P 1 structure which comprised the starting material [43], this is illustrated in Figure 7. The observation of Bragg peaks for TPA but not IPA suggests that the IPA present in the mixture has been rendered amorphous (and therefore does not provide detectable Bragg peaks in the XRPD trace), or perhaps that a new material has been formed which is a nano-crystalline hybrid of TPA/IPA with a very similar crystal structure to that of the C2/c variant of TPA. With only two very broad Bragg peaks evident in the trace there is limited information available. The DSC data showed a clear melting event around 340 °C—if this is indeed due to IPA then it appears that the IPA which was undetectable in XRPD has recrystallised upon heating in the DSC, but if this was the case we might expect to see an exothermic event in the DSC trace, which is not observed. It may be that the XRPD and DSC data in combination point to a new TPA/IPA nano-crystalline hybrid, which has a melting point close to that of IPA. Further work will be necessary to clarify this point.

XRPD for co-milling and separate milling of TPA/PA are shown in Figure 6b. Again, there is a marked difference between the outcomes of co-milling and separate milling. Separate milling produces (unsurprisingly) a trace that appears to be a linear combination of the two components. In contrast co-cryo-milling leads to a XRPD trace which features broad peaks, and which clearly does not look like a simple mixture of the two components (for example, the main peak for TPA at 27.8° is not observed). The trace for the co-cryo-milled material instead resembles that for cryo-milled PA, albeit with broader peaks. It appears that in this case PA has remained relatively crystalline but TPA has been rendered amorphous. The co-cryo-milled trace remains unchanged upon storage, i.e., TPA remains amorphous and PA does not exhibit any obvious peak sharpening. Comparing with the data collected after the materials were cryo-milled by themselves, it appears that in this case co-cryo-milling stabilises the nano-crystalline form of PA and also the amorphous form of TPA. The unexpected endothermic thermal event around 280 °C observed in DSC may therefore represent the melting of a meta-stable polymorph of TPA formed by recrystallisation upon heating, although the lack of an obvious recrystallisation exotherm in the DSC trace is puzzling.

XRPD for the IPA/PA system are given in Figure 6c. Co-cryo-milling again leads to a markedly different XRPD trace compared to separate milling, with the trace at zero weeks not showing any peaks due to IPA but instead resembling a broadened version of PA (similar in fact to the TPA/PA system in this respect). After three weeks of storage, however, the peaks expected due to IPA re-appear and the XRPD trace now resembles a mixture of cryo-milled IPA and PA, albeit with slightly broader peaks. We conclude that PA has again resisted conversion to the amorphous form but has been rendered nano-crystalline by co-cryo-milling (which was not observed as clearly when it was cryo-milled by itself), IPA has been rendered amorphous by co-cryo-milling but then was able to recrystallise upon further storage. We note that IPA was not rendered amorphous when cryo-milled by itself but rather became nano-crystalline.

Overall it is clear that co-cryo-milling leads to a different outcome compared to separate cryo-milling and that it encourages loss of crystallinity in a way that separate cryo-milling does not. Whether this leads to formation of nano-crystalline material (PA when co-cryo-milled) or amorphous material (IPA with both TPA and PA, TPA with PA) seems to link back to the ease with which the materials were rendered nano-crystalline upon separate milling. The observation of a different polymorph of TPA to the starting form when co-cryo-milled with IPA is unexpected. Certainly, for the materials studied in this paper co-cryo-milling produces a synergistic effect compared to milling components individually when crystallinity reduction is considered.

#### 2.2.3. ATR-FTIR Experiments

Figure SI-ATR-FTIR presents the ATR-FTIR data from the co-cryo-milled samples of TPA/IPA, TPA/PA and IPA/PA. There are no drastic changes in the spectra when any of the plotted comparisons are inspected (co-cryo-milled at zero and three weeks; separately cryo-milled at zero and three weeks; co-cryo-milled vs. separately cryo-milled both at zero weeks; and as-received vs. co-cryo-milled). Interpretation of any subtle changes in the spectra is complicated by the presence of two components. We summarise these results by stating that there are no unexpected peak shifts to note.

## 3. Discussion

### 3.1. Single Components

Both TPA and IPA show clear evidence in terms of strong broadening of XRPD peaks for a change to a nano-crystalline form immediately upon cryo-milling. This change did not occur to the same extent for PA. No obvious amorphisation was observed, which suggests that at most only a small fraction of the sample was rendered amorphous upon cryo-milling. Subsequent sharpening of Bragg peaks upon three weeks storage was noted for all samples, with the biggest change occurring for IPA. TPA in contrast retained notable peak broadening even after this time. Despite TPA showing the largest change in XRPD trace upon cryo-milling, it showed the smallest change in carbonyl peak position from ATR-FTIR spectroscopy. IPA showed a red-shift in the carbonyl peak upon cryo-milling and indeed after storage for three weeks, PA in contrast showed a blue-shift upon cryo-milling and no subsequent change upon storage. TPA and IPA showed evidence in DSC of recrystallisation of some material upon heating, there was a possible glass transition observed for PA.

The three materials show surprisingly variable behaviour upon cryo-milling given their similar chemical structures, it is however clear that cryo-milling does change the initial crystalline starting material and a change to a nano-crystalline form (from XRPD), likely with crystal defects, occurs for both TPA and IPA whereas PA seems to resist this. It may be that TPA and IPA can support a higher concentration of crystal defect than PA if these defects are stabilised by improved inter-molecular hydrogen-bonding compared to the crystalline state, where lattice energy is maximised instead. If this is the case PA will be less able to support crystal defects than TPA and IPA due to it having one of the two -CO_2_H groups forming an intra-molecular hydrogen bond which cannot easily become involved in inter-molecular hydrogen bonding upon formation of crystalline defects. These defects would therefore be of prohibitively high energy for PA compared to TPA and IPA.

### 3.2. Multiple Components

A nano-crystalline material was formed when TPA/IPA were co-cryo-milled, with a XRPD diffraction pattern which was markedly different to that of either of the starting materials or their cryo-milled forms. The most likely explanation is that the C2/c polymorph of TPA was formed while IPA was rendered amorphous. For co-cryo-milling involving PA the Bragg peaks of PA were markedly broadened, whereas the Bragg peaks for TPA or IPA were no longer detectable immediately after this treatment. While there is little evidence that co-amorphisation has happened, the addition of PA to TPA and IPA clearly enhances their propensity to turn amorphous upon cryo-milling. Likewise, the Bragg peaks of PA were broader for co-cryo-milling than when it was cryo-milled by itself, so multi-component milling must create a synergistic effect between components. Again, TPA seemed to be the slowest at reverting towards its pre-cryo-milled state, with no TPA-related Bragg peaks visible three weeks after co-cryo-milling with PA.

DSC data did not provide any clear evidence for recrystallisation of either TPA and IPA in the mixtures, despite the observation of a melting event ascribed to IP in the IPA/PA system. The TPA/PA DSC indicated a new unexplained endothermic event around 280 °C which is tentatively assigned to melting of a meta-stable polymorph of TPA.

## 4. Materials and Methods 

### 4.1. Materials

Terephthalic acid (A12527, batch no.-10180244, +98%), Isophthalic acid (A14445, batch no.-B25×004, +99%), and Phthalic acid (A14450, batch no.-10178989, +99%) were obtained from Alfa Aesar. In order to prepare a 43% relative humidity atmosphere for the cryo-milled samples to be kept in throughout the stability study, potassium carbonate (NFPA 704) was purchased from Minerals-Water Ltd.

Terephthalic acid (1 g), isophthalic acid (1 g) and phthalic acid (1 g) were cryo-milled individually for a total of time of 60 min. Physical pair mixtures were prepared by cryo-milling each of the above material alone for 60 min followed by mixing similar proportion of 1:1 triturated by using mortar and pestle. The cryo-milled together mixtures were prepared by weighing 500 mg of each individual material (a 1:1 molar ratio as the molecular masses are the same), the mixture was then cryo-milled for 60 min.

### 4.2. Methods

Initially milling and co-milling at room temperature was attempted using a Retsch MM400 ball mill. The mill was equipped with a stainless steel 10 mL grinding jar and an 8 mm diameter stainless steel ball (approximate measured mass 2 grams), milling was undertaken on 200 mg of sample for 60 min at 30 Hz. This simply led to particle size reduction.

Cryo-milling was achieved by using SamplePrep 6870 Freezer/Mill (SPEX, Metuchen, NJ, USA). It is supplied with an electromagnetic coil which can shuttle the “6751P stainless steel impactor bar” back and forward inside the 25 mL polycarbonate “6751C4 sample vial” in order to be ground against the stainless steel “6751E end caps”. The impactor bar is approximately 60 mm in length and 9.5 mm in diameter and has a measured mass of approximately 32 grams. The liquid nitrogen submerges the coil and vial to keep the sample temperature as low as possible. The cryo-milling time was 60 min for all samples. The instrument was set to pre-cool for 5 min then milling cycles starts for 2 min each at frequency of 15 cycles per second. Throughout the grinding process, there was a pause of 1 minute between cycles to prevent temperature rise. Four samples were cryo-milled in parallel using the “6751 small grinding vial set” The cryo-milled samples were then vacuum sealed inside a dry glove box in order to minimise moisture uptake, it is supplied with an argon cylinder to achieve minimum relative humidity. Photographs of the impactor bar for the cryomill and the milling ball for the Retsch mill are included in SI.

A Q2000 Differential Scanning Calorimeter (DSC) from TA Instruments, New Castle, DE, USA was used for thermal analysis with a constant purge of nitrogen gas of 50 mL per minute. Tzero aluminium hermetic pans and lids were used to contain the samples with a sample size of 5 mg. These pans were sealed in an argon purged dry-box and tapped lightly to spread out the sample evenly within the pans. The instrument was calibrated for temperature and enthalpy using indium. The standard or conventional DSC mode was adopted to measure Tg, melting points, recrystallisation and enthalpy. Modulated DSC would have provided extra information (reversing and non-reversing components of heat flow) but would have led to unreasonably long runs times compared to conventional DSC (1 °C/min vs. 10 °C/min heating rates). This in turn would have affected sample preparation by removing the ability to analyse our samples on their day of preparation when four samples were cryo-milled in parallel as in the current report. It was therefore decided to employ conventional DSC only. Measurements were run from −40 °C to specific temperatures above each component’s melting temperature with specific heating rates except for terephthalic acid, the temperature was raised to around 380 °C and no melting endotherm was detected. Analysis was carried out through the Universal Analysis Software (TA Analysis) to estimate the enthalpy, onset and peak of thermal events such as the glass transition, melting and recrystallisation. Experiments were carried out in triplicate (*n* = 3).

The XRPD was measured by using flat-plate diffractometer, the X’Pert PRO (PANalytical, Almelo, The Netherlands). Reflection mode was set up using Cu Kα1 (λ = 1.54 Å) operating in Bragg-Brentano geometry. 0.0260 2θ/° was used as a step size with a total collection time of 5 min. The voltage of the generator was set to 40 kV and the current to 40 mA. Brass was used as a holder for the samples. The brass plate did lead to two peaks being observed in the majority of the XRPD traces due to brass. To remove these a brass pattern was collected and peak-fitted (using two Gaussians), this model trace was then suitably scaled and subtracted from all affected traces. Full details are given in the computer code in SI.

The IR spectra of the samples were collected using Agilent/Cary630/FTIR, United States. Around 5–8 mg of the samples were applied to the Diamond ATR type II crystal plate. The signal reflection diamond has a 1 mm diameter sampling surface with 200 µm^2^ active area and provides approximately 2 µm depth of penetration for infrared energy at 1700 cm^−1^. The spectral range employed was 4000–500 cm^−1^.

With the intention of studying the stability of the cryo-milled samples, a saturated solution of potassium carbonate was prepared and then kept in a gas-tight environment to achieve the required humidity of 43% at room temperature [44]. The results that will be presented in this study are for the freshly cryo-milled samples and for the samples after subjected to storage for three weeks, the samples were analysed regularly by XRPD, FTIR and DSC after 1, 2 and 3 weeks of storage.

All data analysis was undertaken using the “R” software environment [45], all raw data and associated numerical routines can be found in the supplementary information, including all code and analyses used to generate the figures contained in the current paper. Data were scaled by mean-centering and then divided by their standard deviations for plotting (using the “scale” command in R).

## 5. Conclusions

Attempts to amorphise TPA, IPA and PA and to co-amorphise their pair-wise combinations led to a surprisingly wide range of behaviour given that they are all chemically extremely similar. While co-amorphisation was not observed, strong synergy was observed when co-cryo-milling the two-component systems with amorphisation and/or particle size reduction being greatly enhanced compared to milling the individual materials. The apparent conversion of the TPA in TPA/IPA (but not in TPA/PA) to a different polymorph to the starting material was unexpected. No previous reports of any amorphisation of TPA, IPA or PA are available in the literature despite their production and use being in the region of millions of tonnes per year.

Given the aims of our study were to investigate a simple structural series and gain insight into any rules for producing co-amorphous formulations, we can conclude that very similar chemical structures can behave very differently when subject to amorphisation processes. This may suggest that mechanical properties rather than chemical properties should be a focus for future investigation.

## Supplementary Materials

The following are available online.
