# Supplementary material for: Investigation of Potential Amorphisation and Co-Amorphisation Behaviour of the Benzene Di-Carboxylic Acids upon Cryo-Milling
Source: Molecules. 2019 Nov 5;24(21):3990. doi: 10.3390/molecules24213990 (PMC6865180; doi:10.3390/molecules24213990)
Supplement: Supplementary file 1 [file molecules-24-03990-s001.zip › SI_pack/Burley_Elfakhri_figures.pdf]

TPA

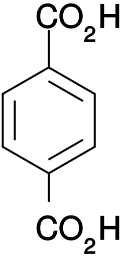

IPA

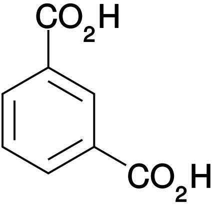

PA

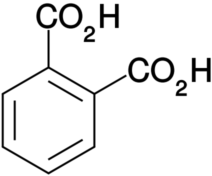

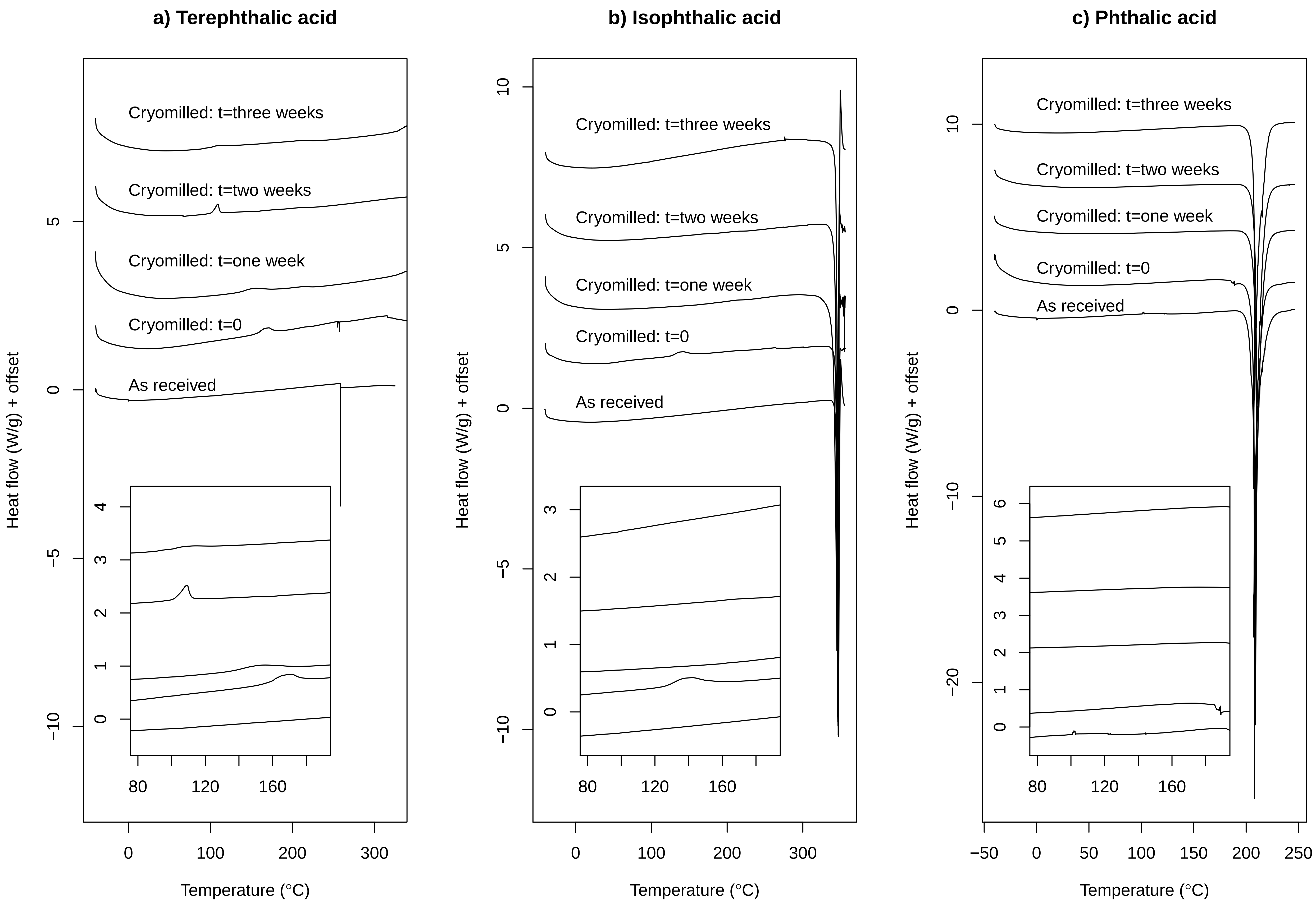

a) Terephthalic acid
b) Isophthalic acid
c) Phthalic acid
Cryomilled: t=three weeks
Cryomilled: t=two weeks
Cryomilled: t=one week
Cryomilled: t=0
As received
Heat flow (W/g) + offset
Temperature (°C)

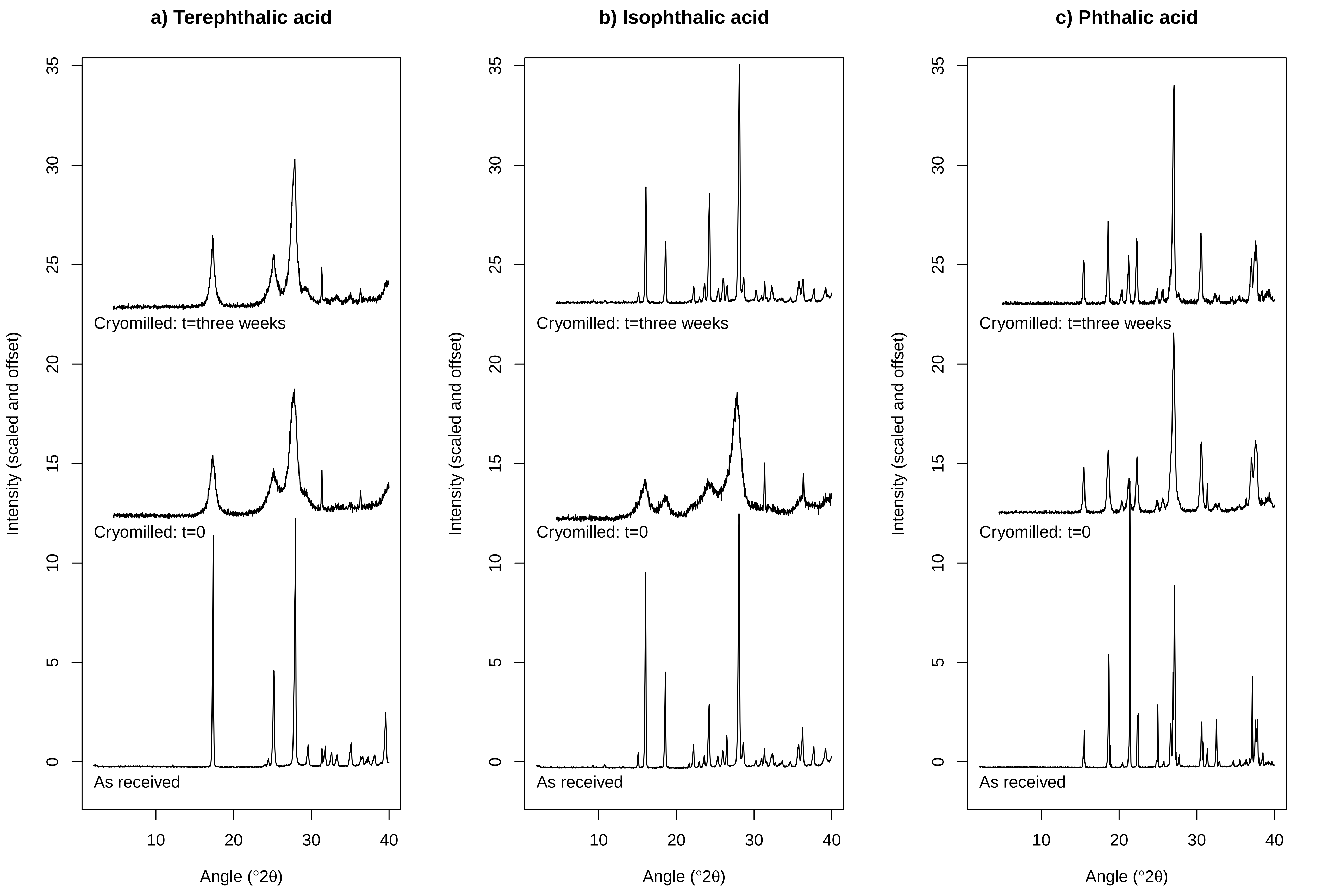
a) Terephthalic acid
b) Isophthalic acid
c) Phthalic acid
Cryomilled: t=three weeks
Cryomilled: t=0
As received
Intensity (scaled and offset)
Angle (°2θ)
0
5
10
15
20
25
30
35
10
20
30
40

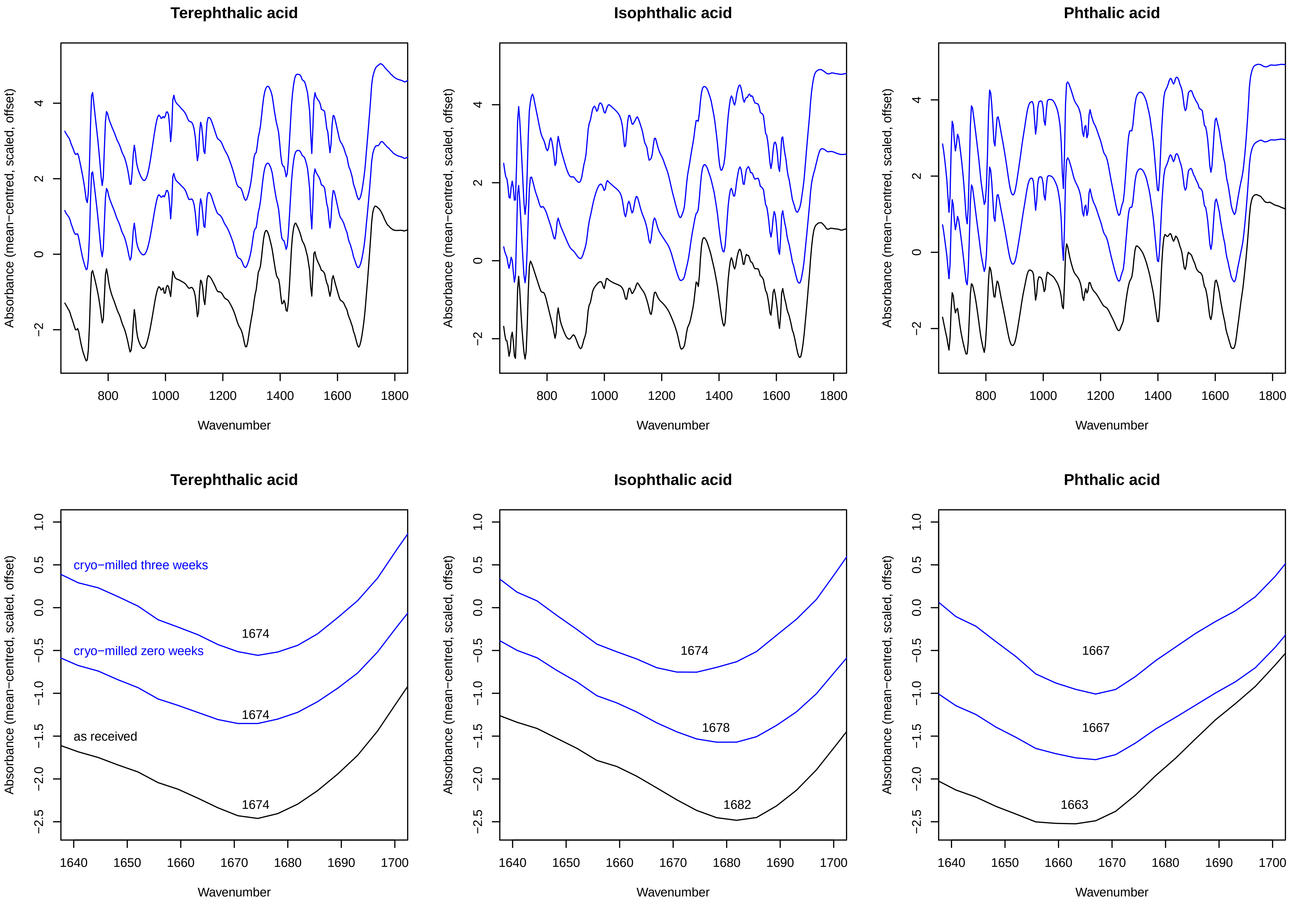
Terephthalic acid
Isophthalic acid
Phthalic acid
Absorbance (mean−centred, scaled, offset)
Wavenumber
800
1000
1200
1400
1600
1800
Terephthalic acid
Isophthalic acid
Phthalic acid
cryo−milled three weeks
cryo−milled zero weeks
as received
1674
1674
1674
1674
1678
1682
1667
1667
1663
Absorbance (mean−centred, scaled, offset)
Wavenumber
1640
1650
1660
1670
1680
1690
1700

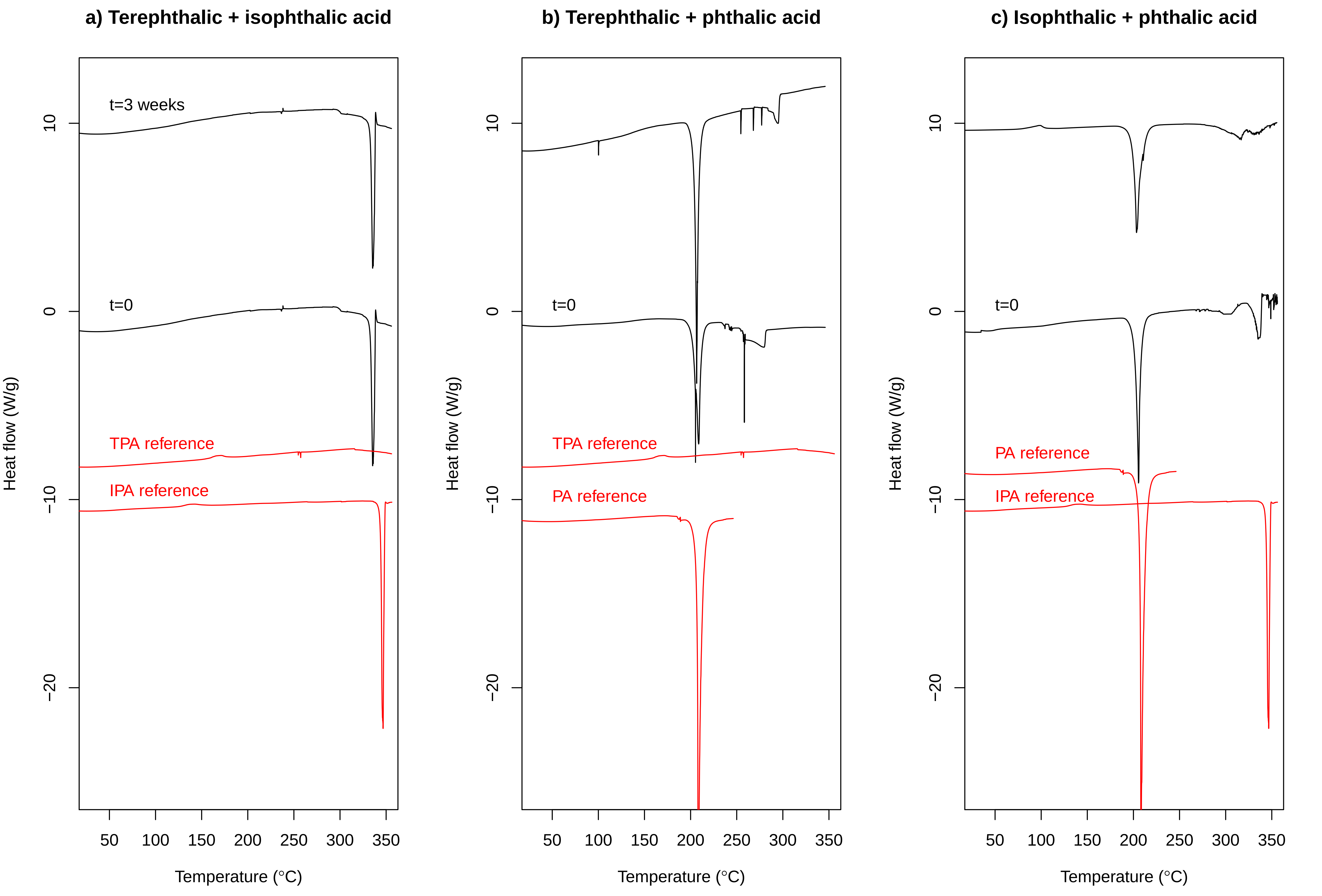
a) Terephthalic + isophthalic acid
b) Terephthalic + phthalic acid
c) Isophthalic + phthalic acid
t=3 weeks
t=0
TPA reference
IPA reference
PA reference
Heat flow (W/g)
Temperature (°C)
10
0
−10
−20
50
100
150
200
250
300
350

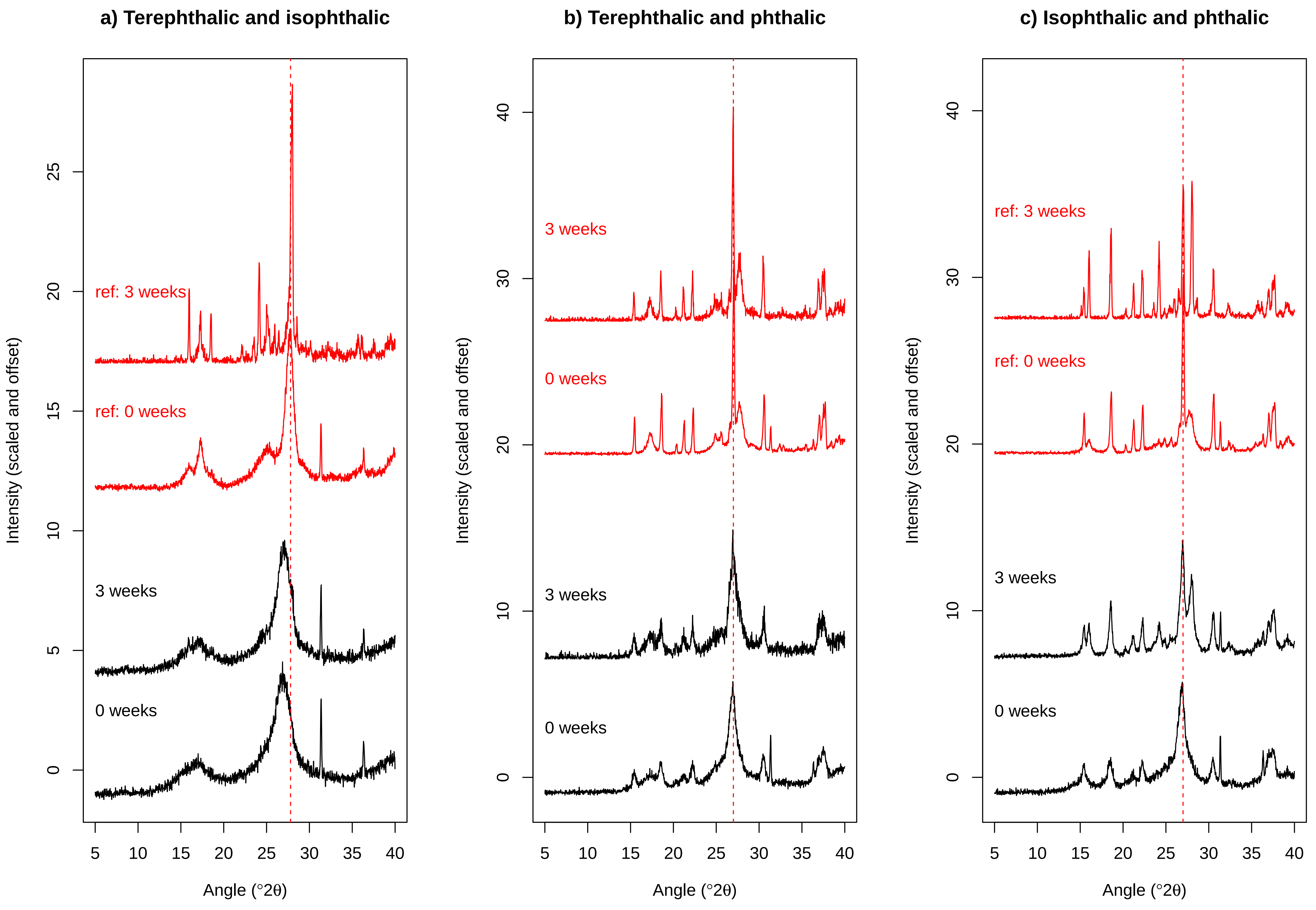

a) Terephthalic and isophthalic
b) Terephthalic and phthalic
c) Isophthalic and phthalic
Intensity (scaled and offset)
Angle (°2θ)
ref: 3 weeks
ref: 0 weeks
3 weeks
0 weeks

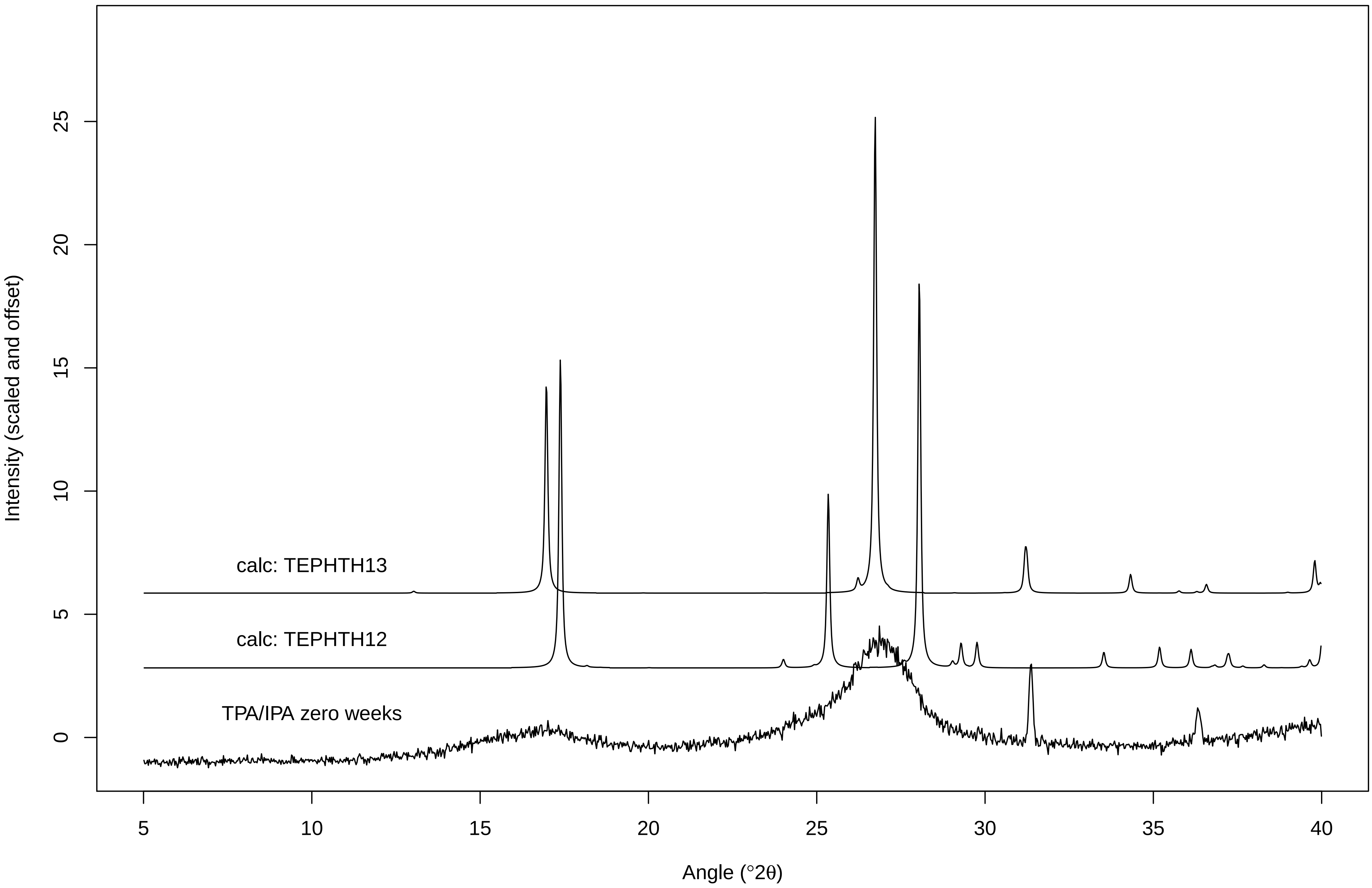

calc: TEPHTH13
calc: TEPHTH12
TPA/IPA zero weeks
Intensity (scaled and offset)
Angle (°2θ)
0
5
10
15
20
25
5
10
15
20
25
30
35
40
