# Supplementary material for: Investigation of Potential Amorphisation and Co-Amorphisation Behaviour of the Benzene Di-Carboxylic Acids upon Cryo-Milling
Source: Molecules. 2019 Nov 5;24(21):3990. doi: 10.3390/molecules24213990 (PMC6865180; doi:10.3390/molecules24213990)
Supplement: Supplementary file 1 [file molecules-24-03990-s001.zip › SI_pack/Figure_4_FTIR_single_components/Data/Isophthalic acid/Isophthalic 2.pdf]

Isophthalic acid cryomilled for 60 mins after 3 weel

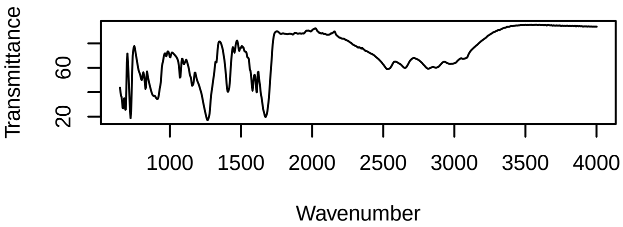

Isophthalic acid cryomilled for 60 mins at zero wee

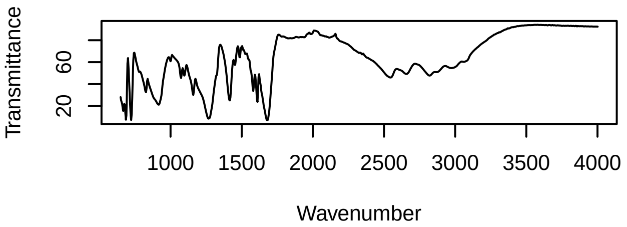

Isophthalic acid as received

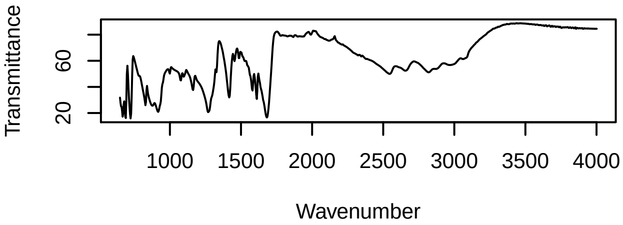

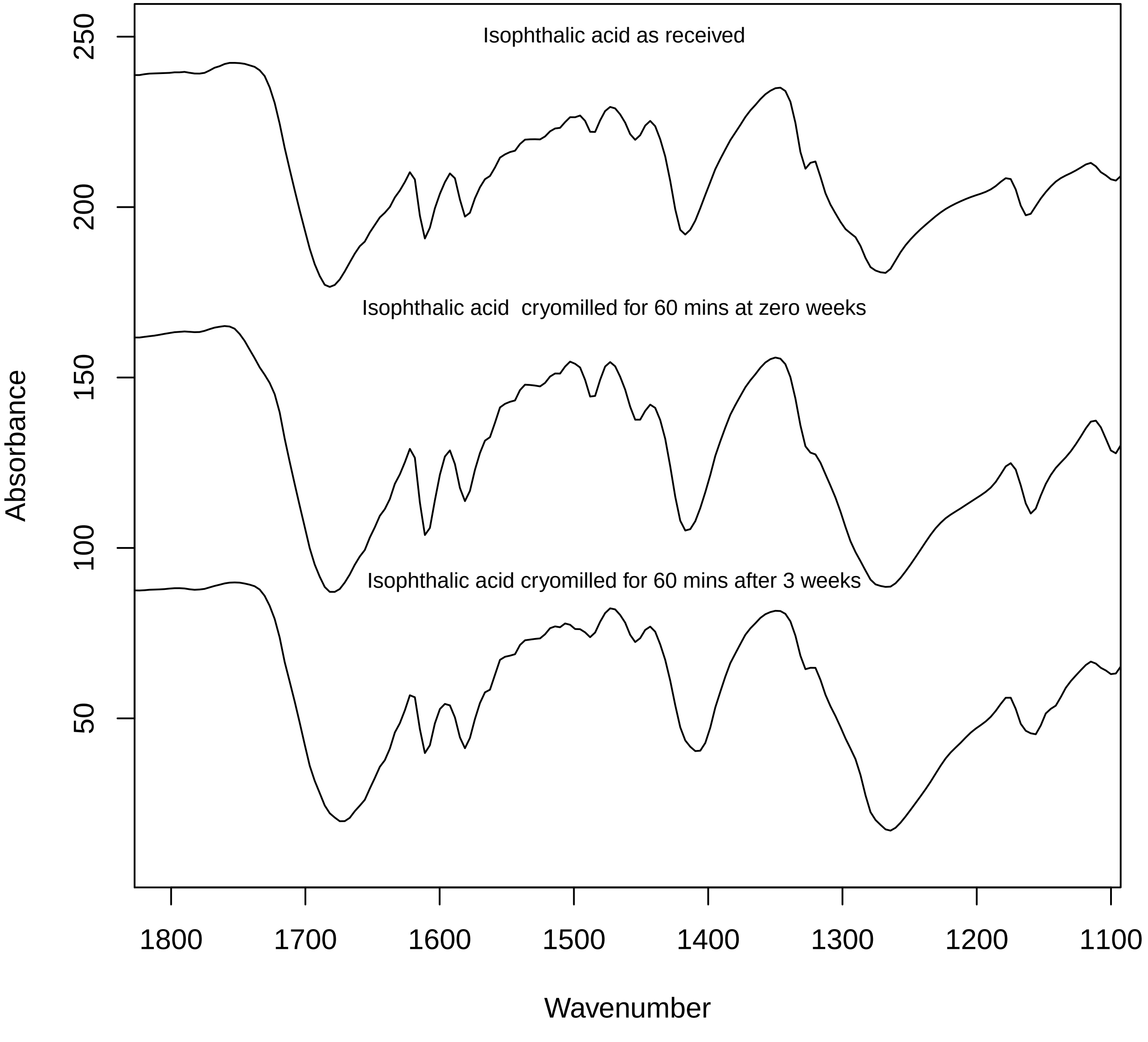
Isophthalic acid as received
Isophthalic acid cryomilled for 60 mins at zero weeks
Isophthalic acid cryomilled for 60 mins after 3 weeks
Absorbance
250
200
150
100
50
1800
1700
1600
1500
1400
1300
1200
1100
Wavenumber
