# Supplementary material for: Investigation of Potential Amorphisation and Co-Amorphisation Behaviour of the Benzene Di-Carboxylic Acids upon Cryo-Milling
Source: Molecules. 2019 Nov 5;24(21):3990. doi: 10.3390/molecules24213990 (PMC6865180; doi:10.3390/molecules24213990)
Supplement: Supplementary file 1 [file molecules-24-03990-s001.zip › SI_pack/Figure_4_FTIR_single_components/Data/Phthalic acid/example rect.pdf]

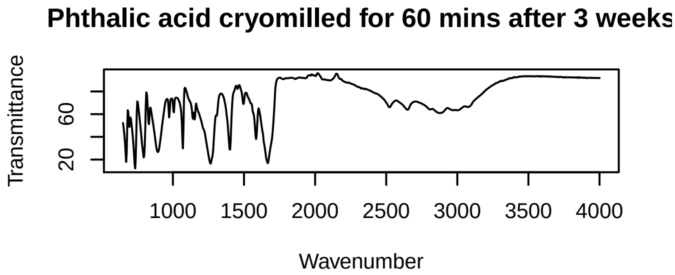
Phthalic acid cryomilled for 60 mins after 3 weeks
Transmittance
20
60
1000
1500
2000
2500
3000
3500
4000
Wavenumber

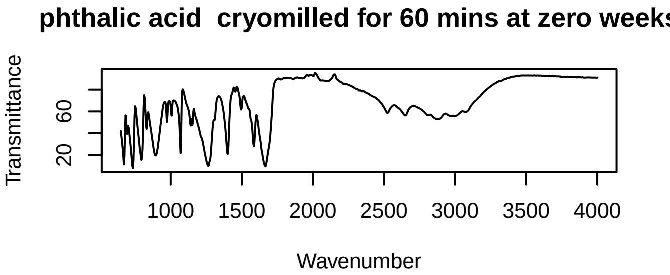
phthalic acid cryomilled for 60 mins at zero weeks
Transmittance
20
60
1000
1500
2000
2500
3000
3500
4000
Wavenumber

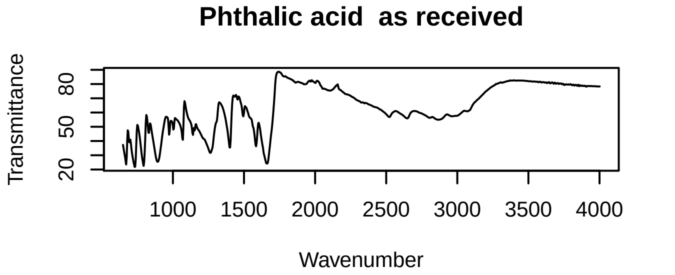
Phthalic acid as received
Transmittance
20
50
80
1000
1500
2000
2500
3000
3500
4000
Wavenumber

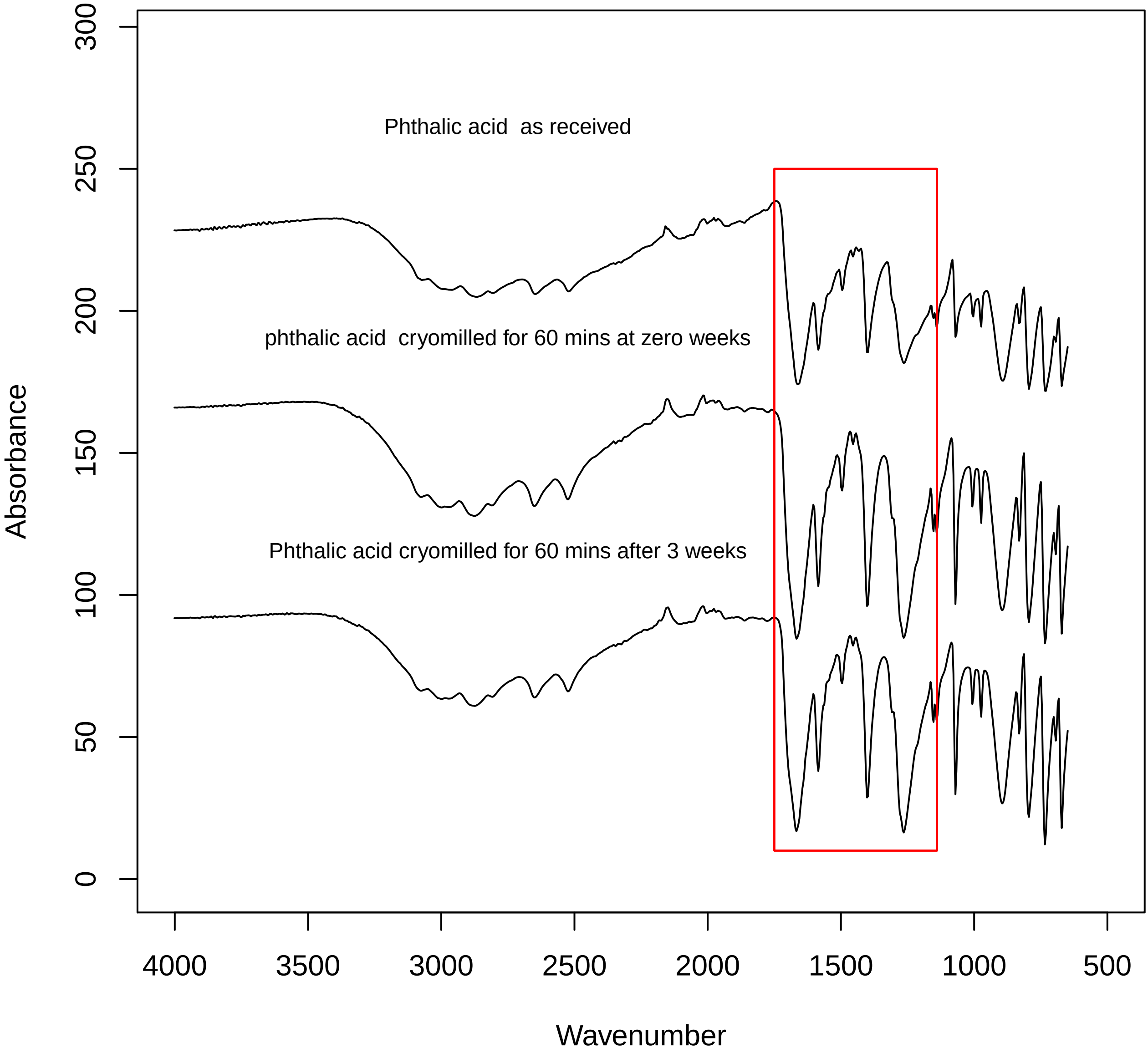

Phthalic acid as received
phthalic acid cryomilled for 60 mins at zero weeks
Phthalic acid cryomilled for 60 mins after 3 weeks
Absorbance
Wavenumber
0
50
100
150
200
250
300
4000
3500
3000
2500
2000
1500
1000
500
