# Supplementary material for: Investigation of Potential Amorphisation and Co-Amorphisation Behaviour of the Benzene Di-Carboxylic Acids upon Cryo-Milling
Source: Molecules. 2019 Nov 5;24(21):3990. doi: 10.3390/molecules24213990 (PMC6865180; doi:10.3390/molecules24213990)
Supplement: Supplementary file 1 [file molecules-24-03990-s001.zip › SI_pack/Figure_4_FTIR_single_components/Data/Terephthalic acid/Terephthalic acid.pdf]

Terephthalic acid CM for 60mins at week 3

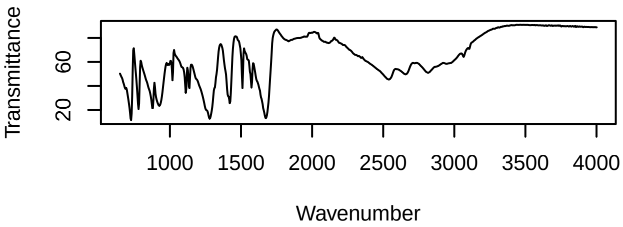

Terepthalic acid CM for 60mins at zero weeks

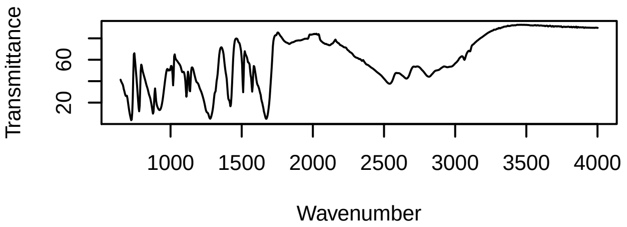

Terephthalic acid as received

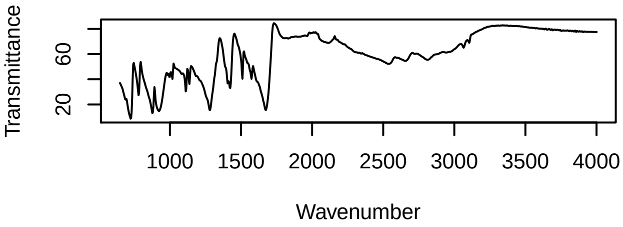

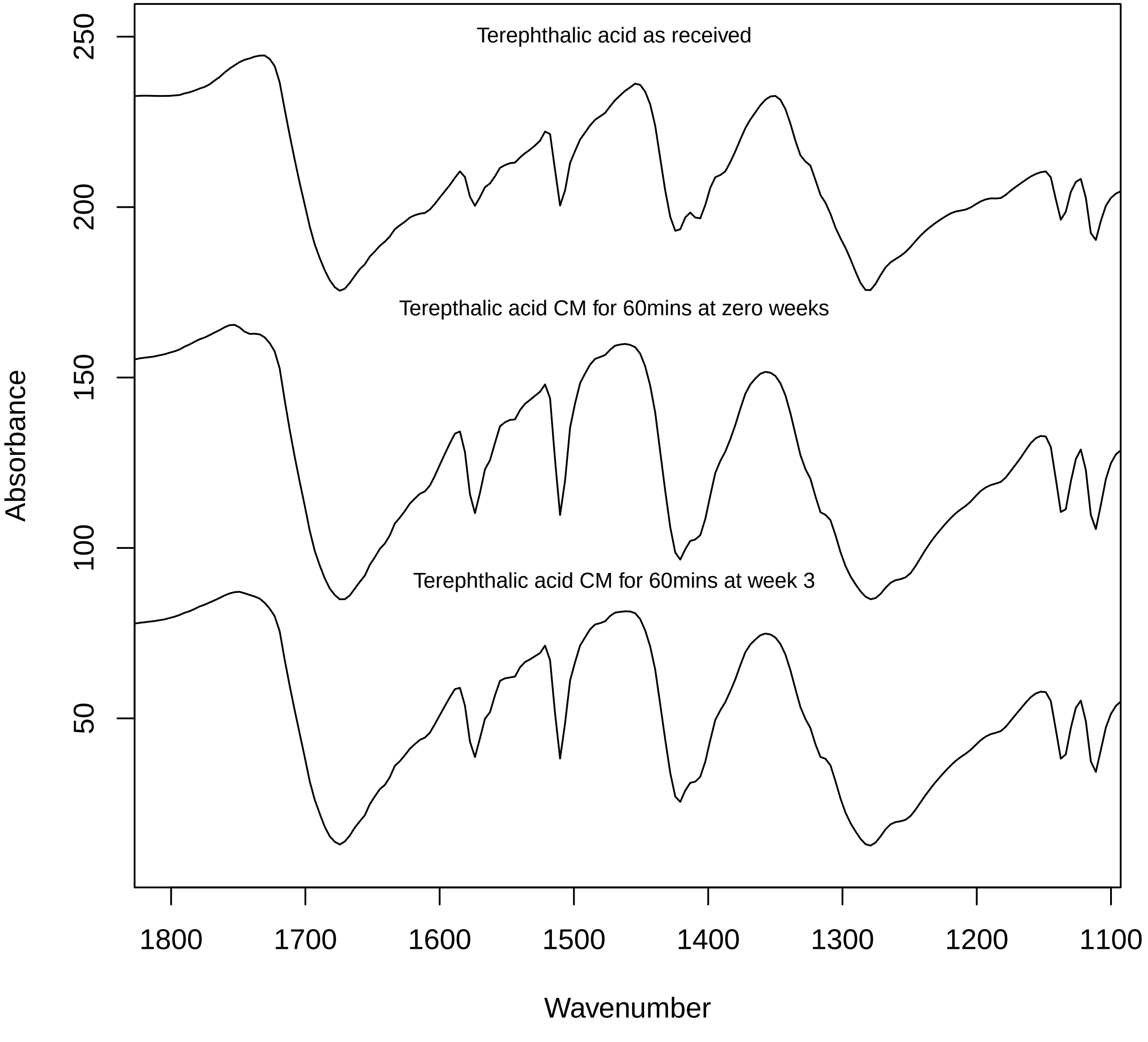

Terephthalic acid as received
Terepthalic acid CM for 60mins at zero weeks
Terephthalic acid CM for 60mins at week 3
Absorbance
250
200
150
100
50
1800
1700
1600
1500
1400
1300
1200
1100
Wavenumber
