# Supplementary material for: Investigation of Potential Amorphisation and Co-Amorphisation Behaviour of the Benzene Di-Carboxylic Acids upon Cryo-Milling
Source: Molecules. 2019 Nov 5;24(21):3990. doi: 10.3390/molecules24213990 (PMC6865180; doi:10.3390/molecules24213990)
Supplement: Supplementary file 1 [file molecules-24-03990-s001.zip › SI_pack/Figure_SI_ATR_FTIR_two_components/Data/IT/it mixtures.pdf]

nthalic and terephthalic acid CM together for 60mins (

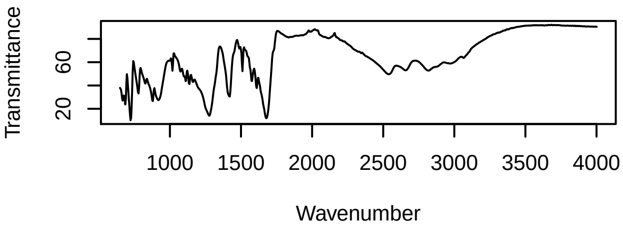

halicand terephthalic acid CM togrther for 60mins (ze

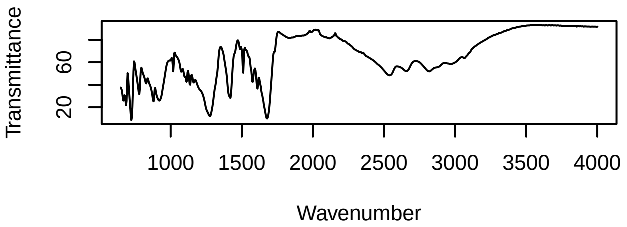

halic and terephthalic acid CM separately for 60mins

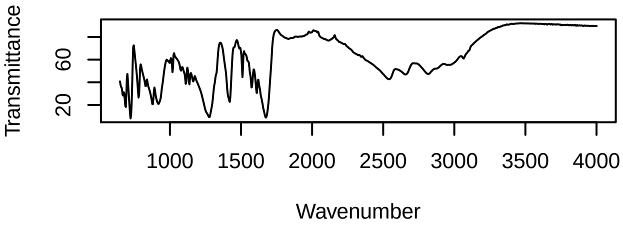

alic and terephthalic acid CM separately for 60mins (z

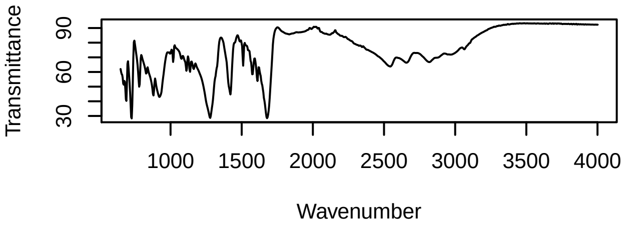

Terepthalic cid CM 60mins

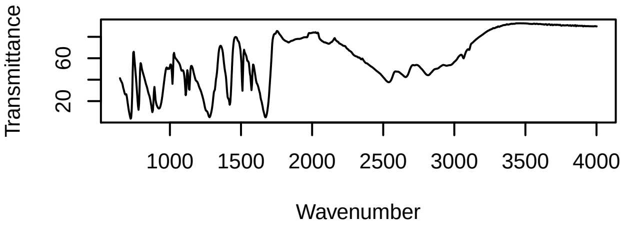

Isophthalic acid CM 60mins

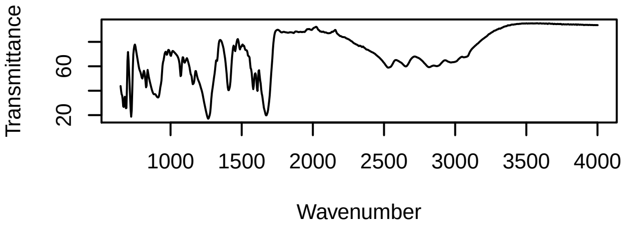

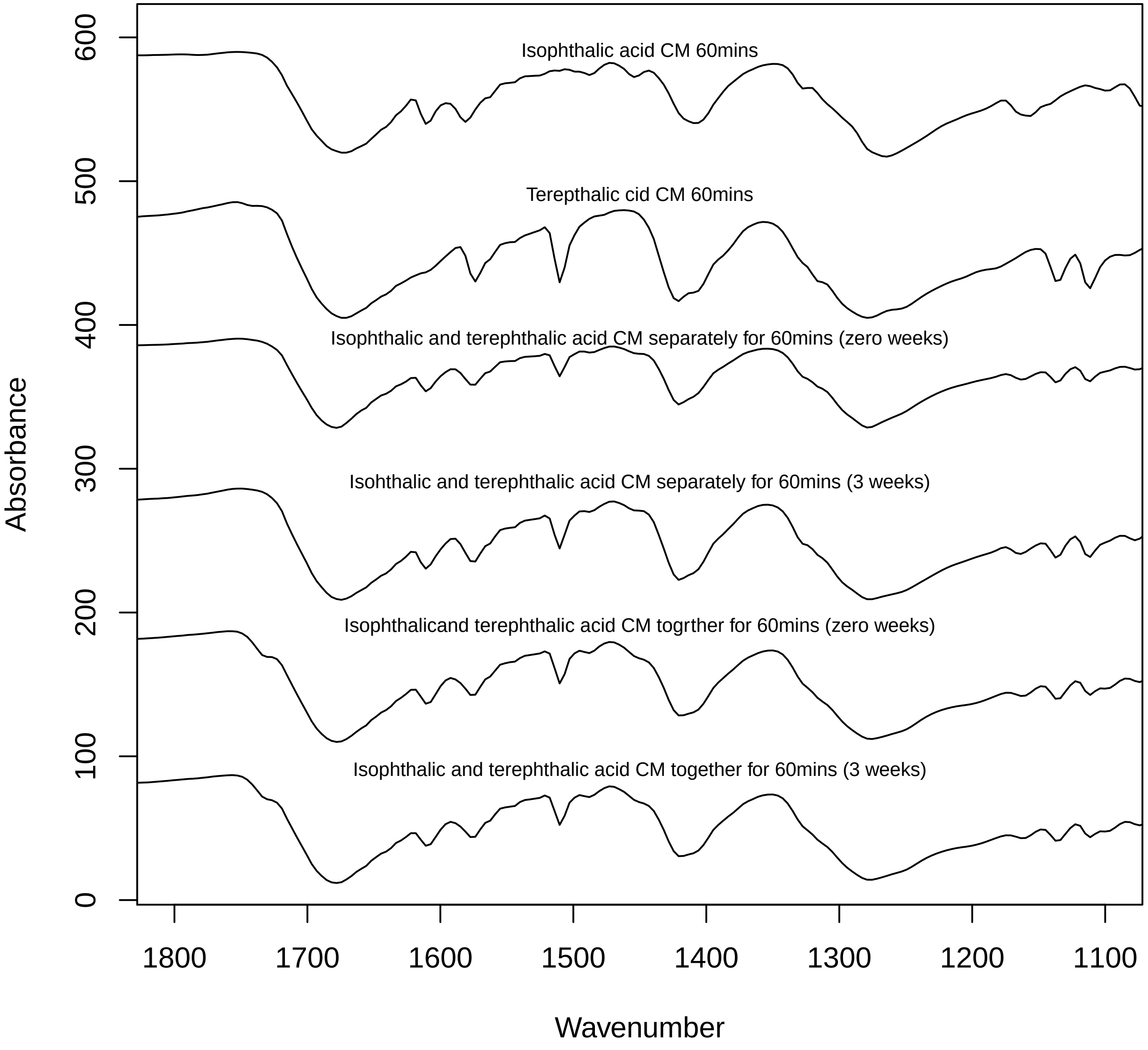

Isophthalic acid CM 60mins
Terepthalic cid CM 60mins
Isophthalic and terephthalic acid CM separately for 60mins (zero weeks)
Isohthalic and terephthalic acid CM separately for 60mins (3 weeks)
Isophthalicand terephthalic acid CM togrther for 60mins (zero weeks)
Isophthalic and terephthalic acid CM together for 60mins (3 weeks)
Absorbance
0
100
200
300
400
500
600
Wavenumber
1800
1700
1600
1500
1400
1300
1200
1100
