# Supplementary material for: Investigation of Potential Amorphisation and Co-Amorphisation Behaviour of the Benzene Di-Carboxylic Acids upon Cryo-Milling
Source: Molecules. 2019 Nov 5;24(21):3990. doi: 10.3390/molecules24213990 (PMC6865180; doi:10.3390/molecules24213990)
Supplement: Supplementary file 1 [file molecules-24-03990-s001.zip › SI_pack/Figure_SI_ATR_FTIR_two_components/Data/IT/IT.pdf]

alic and terephthalic acid CM together for 60mins (af

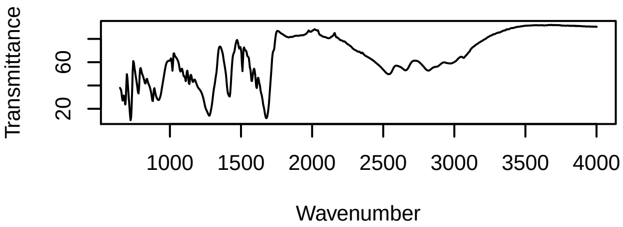

alic and terephthalic acid CM together for 60mins (at :

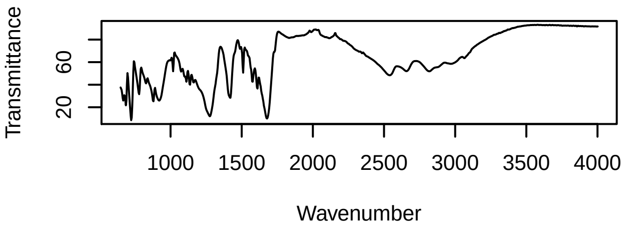

alic and terephthalic acid CM separatly for 60mins (af

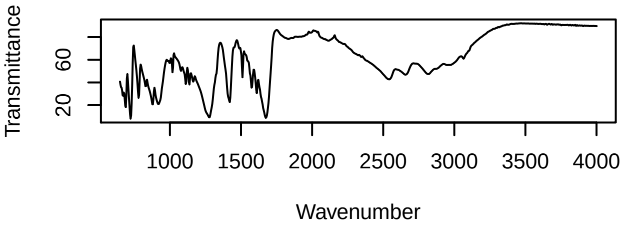

alic and terephthalic acid CM separatly for 60mis (at z

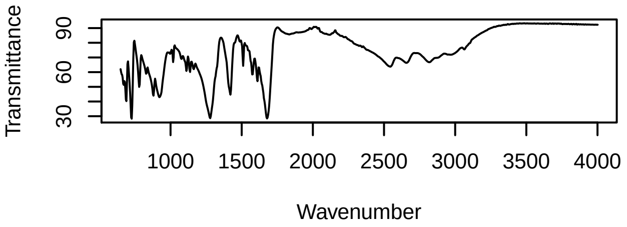

Terephthalic acid CM for 60mins

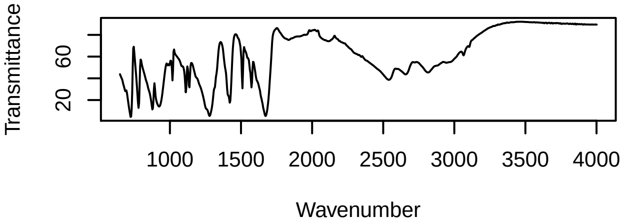

Isophthalic acid CM for 60mins

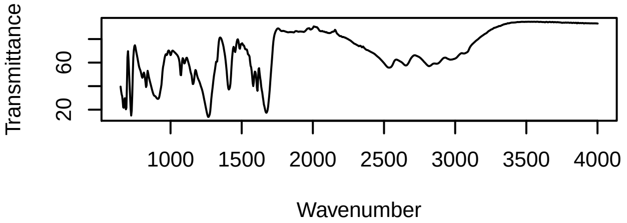

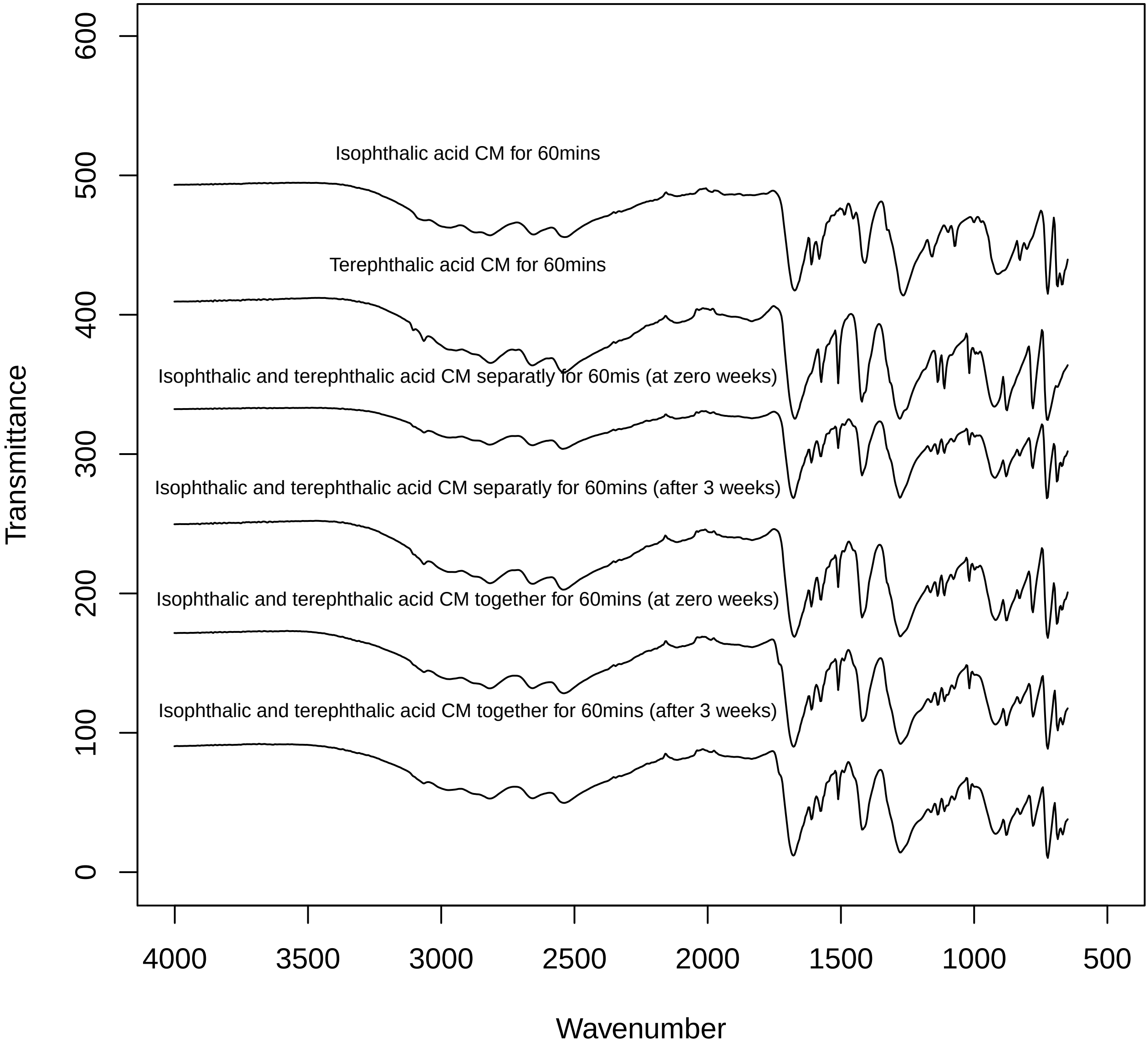
Isophthalic acid CM for 60mins
Terephthalic acid CM for 60mins
Isophthalic and terephthalic acid CM separatly for 60mis (at zero weeks)
Isophthalic and terephthalic acid CM separatly for 60mins (after 3 weeks)
Isophthalic and terephthalic acid CM together for 60mins (at zero weeks)
Isophthalic and terephthalic acid CM together for 60mins (after 3 weeks)
Transmittance
Wavenumber
0
100
200
300
400
500
600
4000
3500
3000
2500
2000
1500
1000
500
