# Supplementary material for: Investigation of Potential Amorphisation and Co-Amorphisation Behaviour of the Benzene Di-Carboxylic Acids upon Cryo-Milling
Source: Molecules. 2019 Nov 5;24(21):3990. doi: 10.3390/molecules24213990 (PMC6865180; doi:10.3390/molecules24213990)
Supplement: Supplementary file 1 [file molecules-24-03990-s001.zip › SI_pack/Figure_SI_ATR_FTIR_two_components/Data/PI/pi mixtures.pdf]

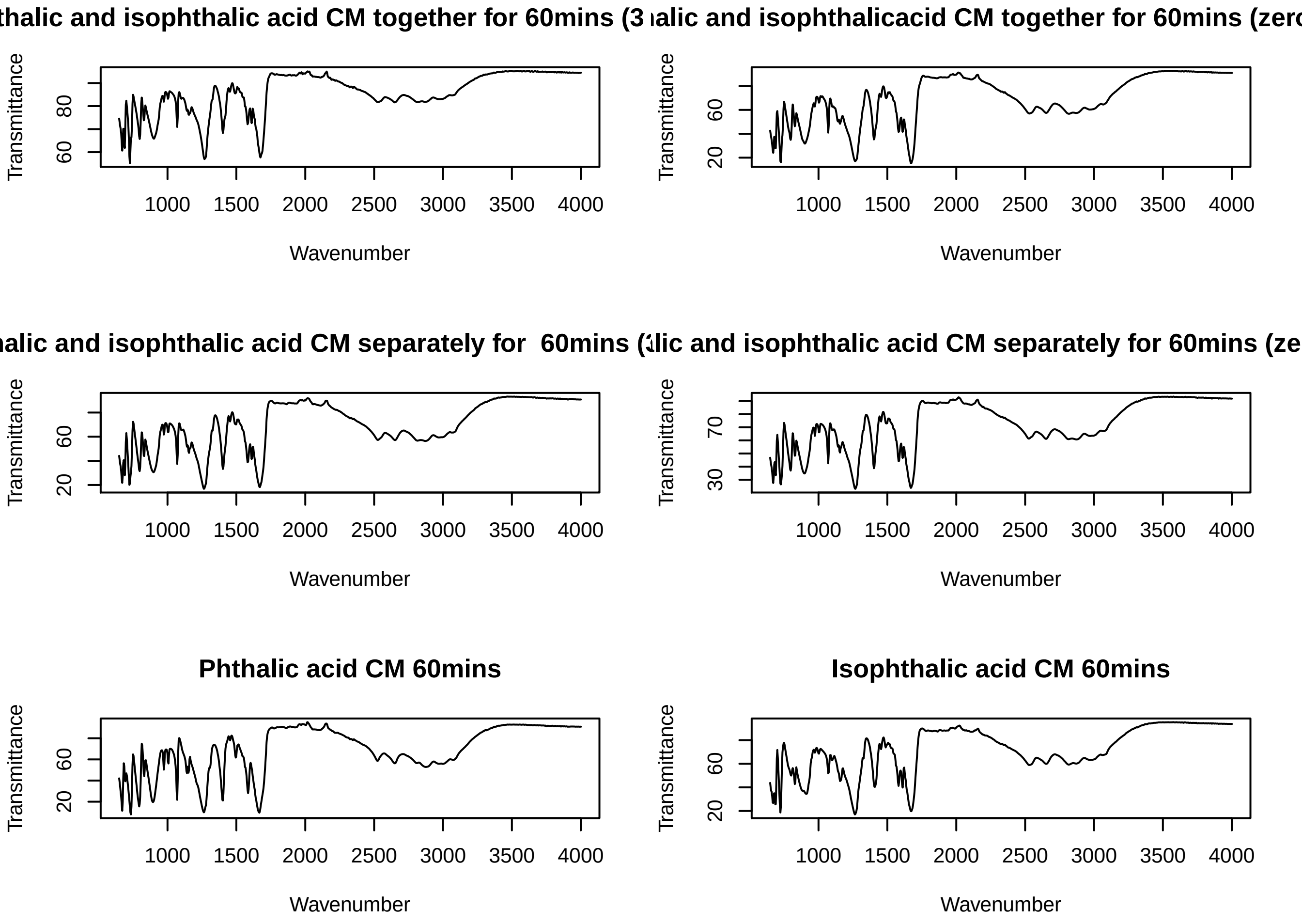
thalic and isophthalic acid CM together for 60mins (3
ıalic and isophthalicacid CM together for 60mins (zerc
ıalic and isophthalic acid CM separately for 60mins (ʒ
ılic and isophthalic acid CM separately for 60mins (ze
Phthalic acid CM 60mins
Isophthalic acid CM 60mins
Transmittance
Wavenumber
1000
1500
2000
2500
3000
3500
4000
20
30
60
70
80

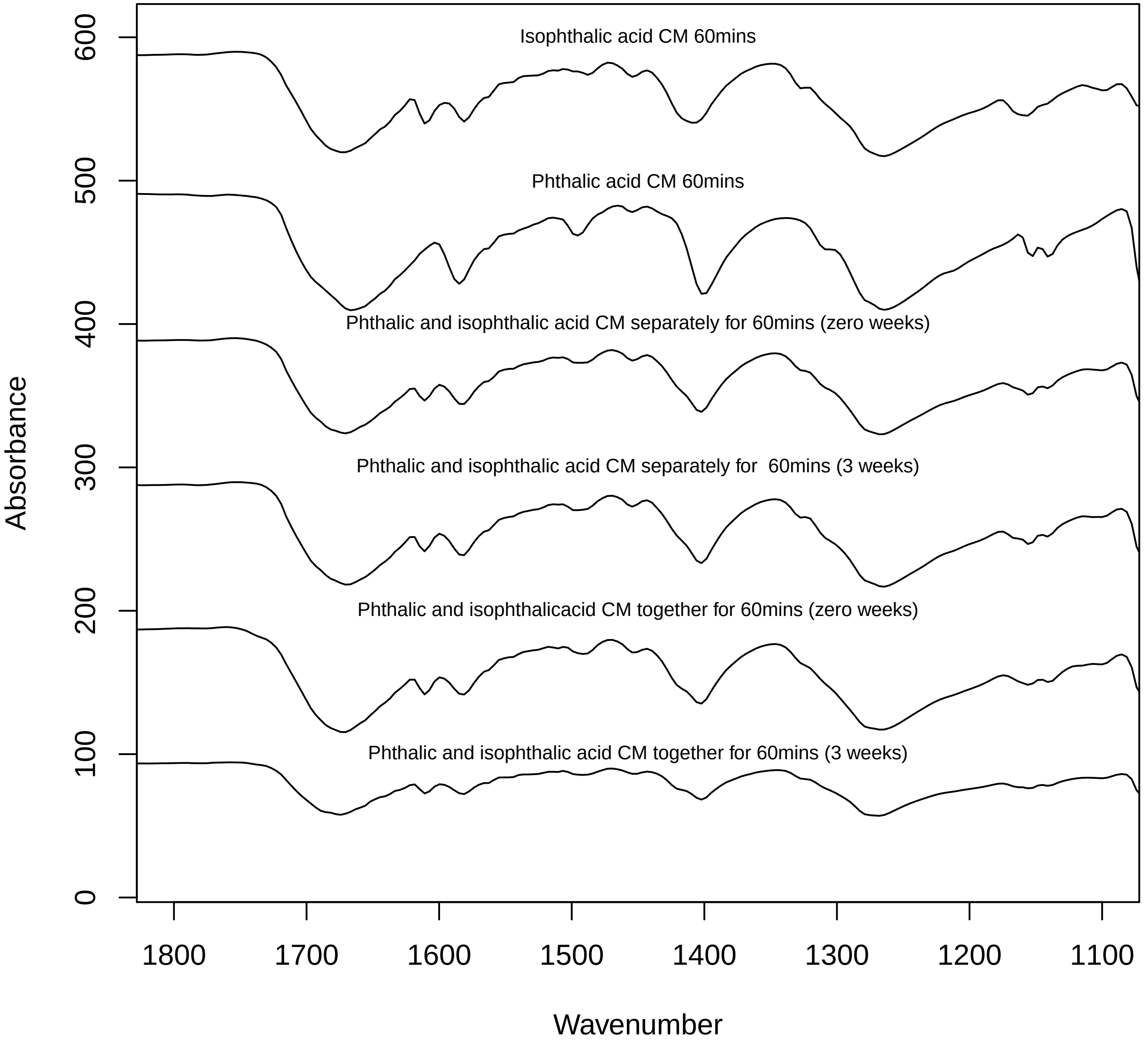
Isophthalic acid CM 60mins
Phthalic acid CM 60mins
Phthalic and isophthalic acid CM separately for 60mins (zero weeks)
Phthalic and isophthalic acid CM separately for 60mins (3 weeks)
Phthalic and isophthalicacid CM together for 60mins (zero weeks)
Phthalic and isophthalic acid CM together for 60mins (3 weeks)
Absorbance
0
100
200
300
400
500
600
Wavenumber
1800
1700
1600
1500
1400
1300
1200
1100
