# Supplementary material for: Investigation of Potential Amorphisation and Co-Amorphisation Behaviour of the Benzene Di-Carboxylic Acids upon Cryo-Milling
Source: Molecules. 2019 Nov 5;24(21):3990. doi: 10.3390/molecules24213990 (PMC6865180; doi:10.3390/molecules24213990)
Supplement: Supplementary file 1 [file molecules-24-03990-s001.zip › SI_pack/Figure_SI_ATR_FTIR_two_components/Data/PI/PI.pdf]

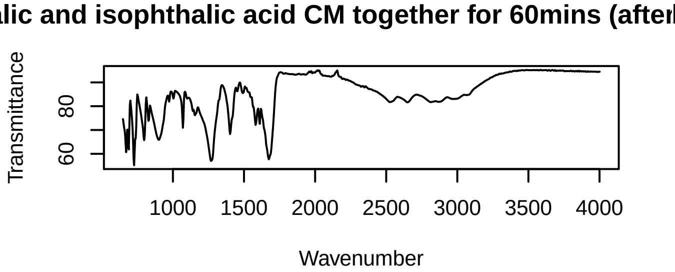
alic and isophthalic acid CM together for 60mins (afte
Transmittance
60
80
1000 1500 2000 2500 3000 3500 4000
Wavenumber

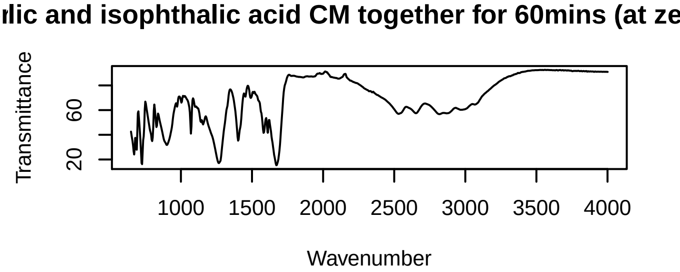
lic and isophthalic acid CM together for 60mins (at ze
Transmittance
20
60
1000 1500 2000 2500 3000 3500 4000
Wavenumber

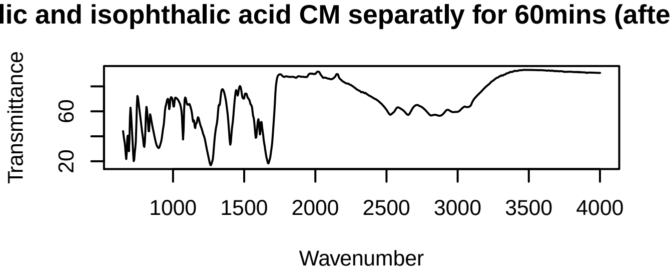
alic and isophthalic acid CM separatly for 60mins (afte
Transmittance
20
60
1000 1500 2000 2500 3000 3500 4000
Wavenumber

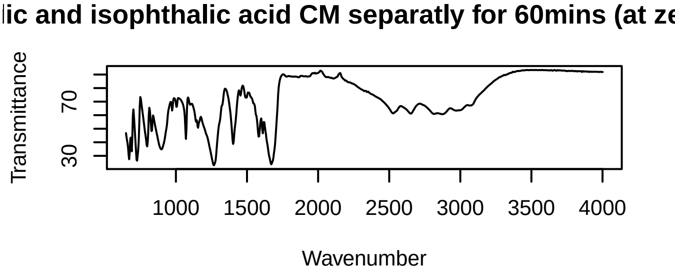
lic and isophthalic acid CM separatly for 60mins (at ze
Transmittance
30
70
1000 1500 2000 2500 3000 3500 4000
Wavenumber

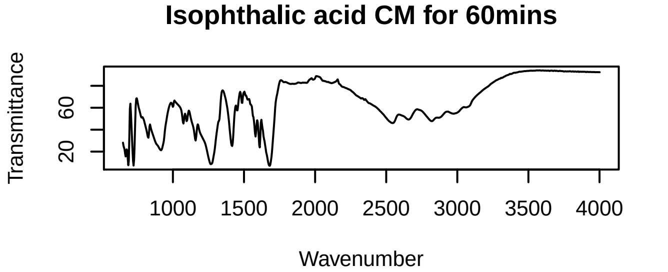
Isophthalic acid CM for 60mins
Transmittance
20
60
1000 1500 2000 2500 3000 3500 4000
Wavenumber

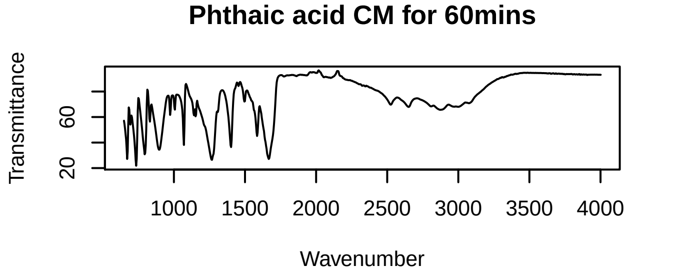
Phthaic acid CM for 60mins
Transmittance
20
60
1000 1500 2000 2500 3000 3500 4000
Wavenumber

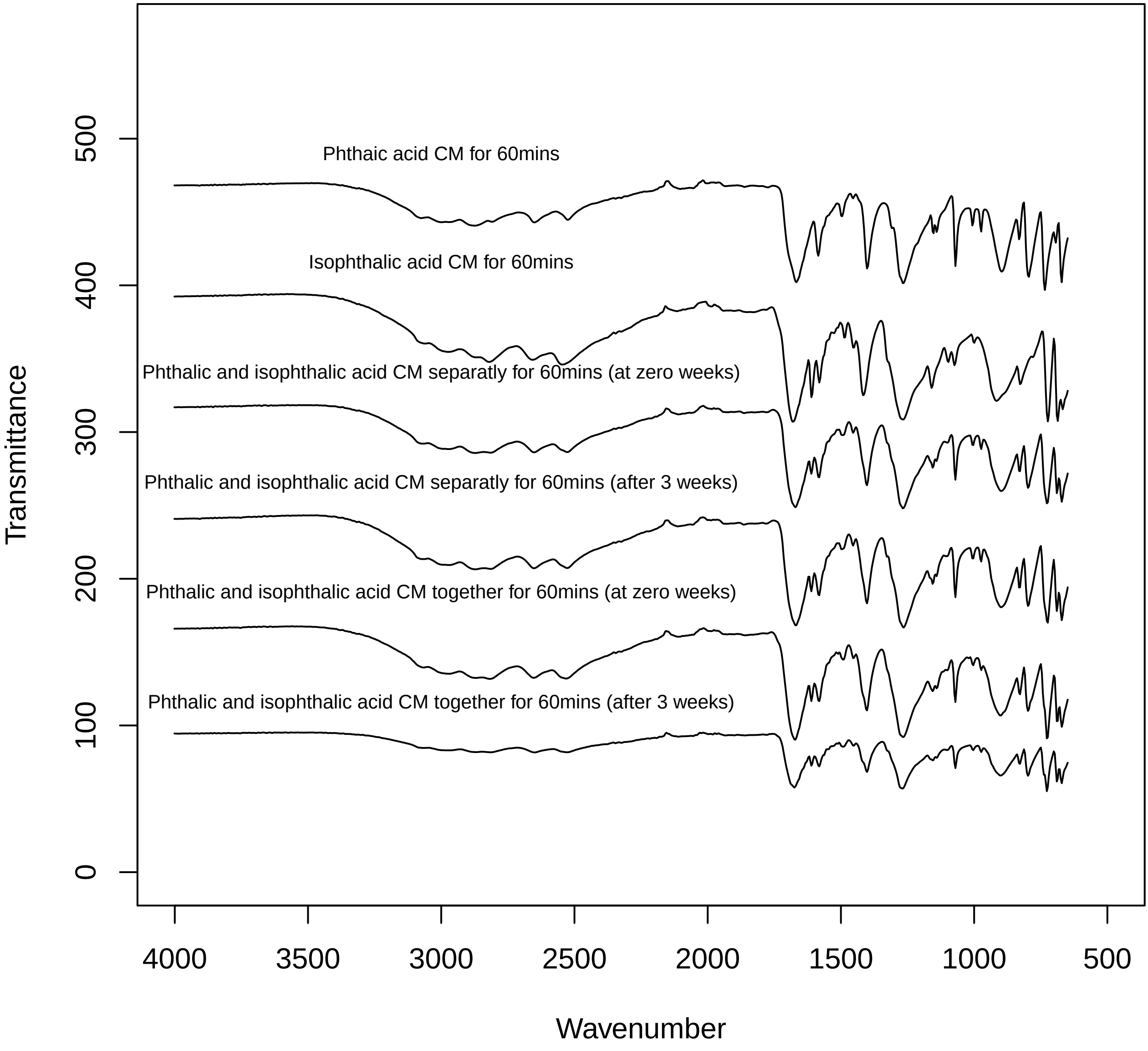

Phthaic acid CM for 60mins
Isophthalic acid CM for 60mins
Phthalic and isophthalic acid CM separatly for 60mins (at zero weeks)
Phthalic and isophthalic acid CM separatly for 60mins (after 3 weeks)
Phthalic and isophthalic acid CM together for 60mins (at zero weeks)
Phthalic and isophthalic acid CM together for 60mins (after 3 weeks)
Transmittance
0
100
200
300
400
500
Wavenumber
4000
3500
3000
2500
2000
1500
1000
500
