# Supplementary figures and images for: Investigation of Potential Amorphisation and Co-Amorphisation Behaviour of the Benzene Di-Carboxylic Acids upon Cryo-Milling
Source: Molecules. 2019 Nov 5;24(21):3990. doi: 10.3390/molecules24213990 (PMC6865180; doi:10.3390/molecules24213990)

**a) Terephthalic acid**

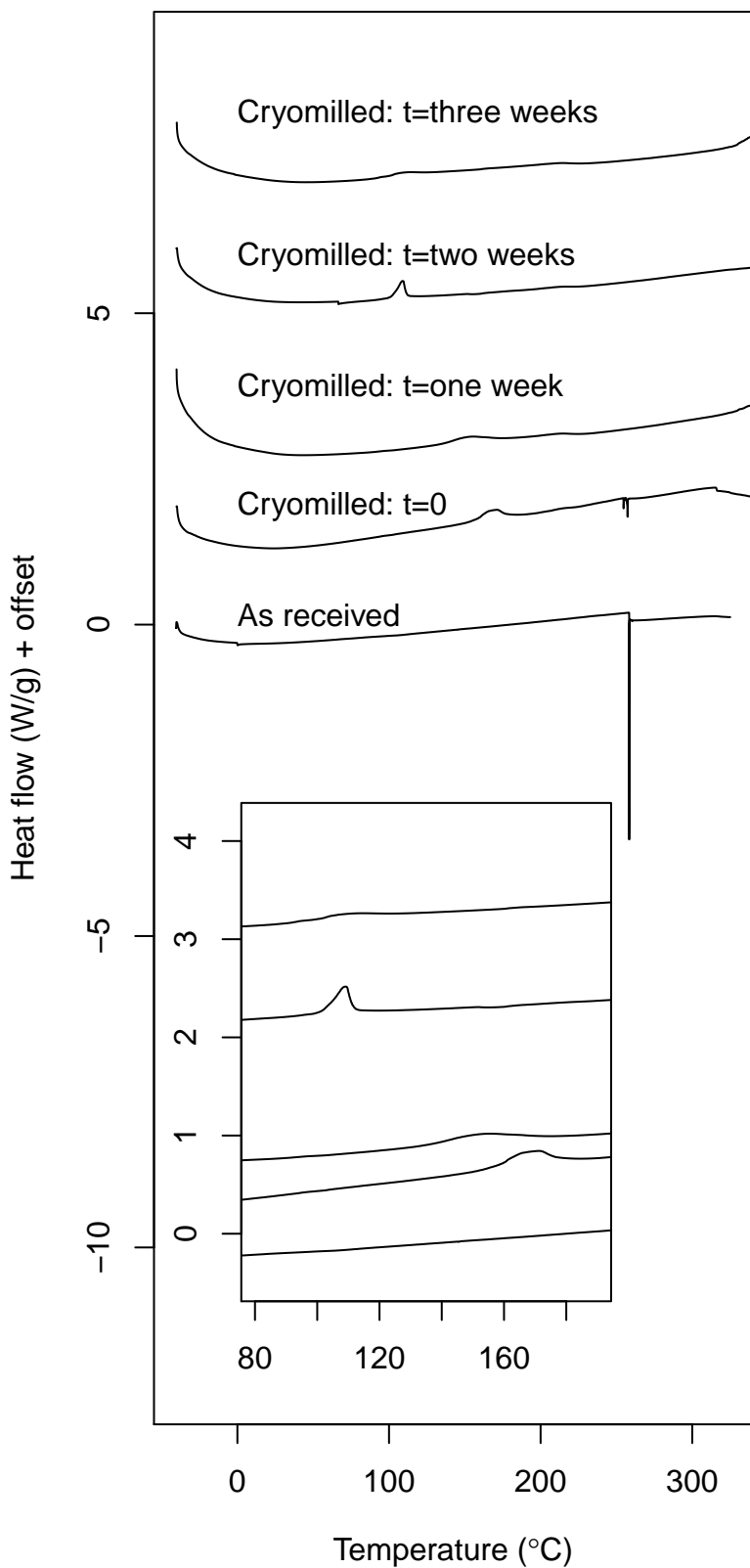

**b) Isophthalic acid**

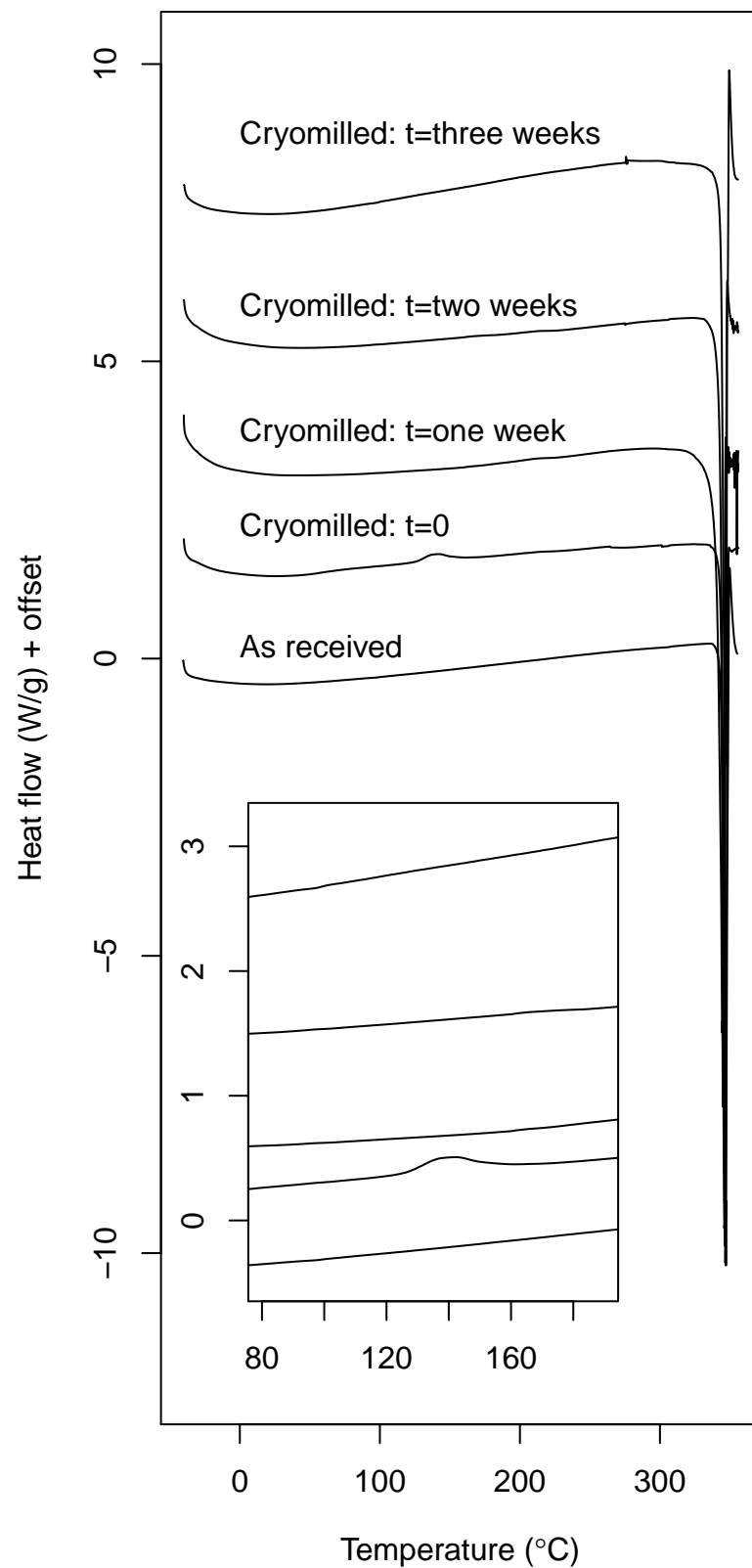

**c) Phthalic acid**

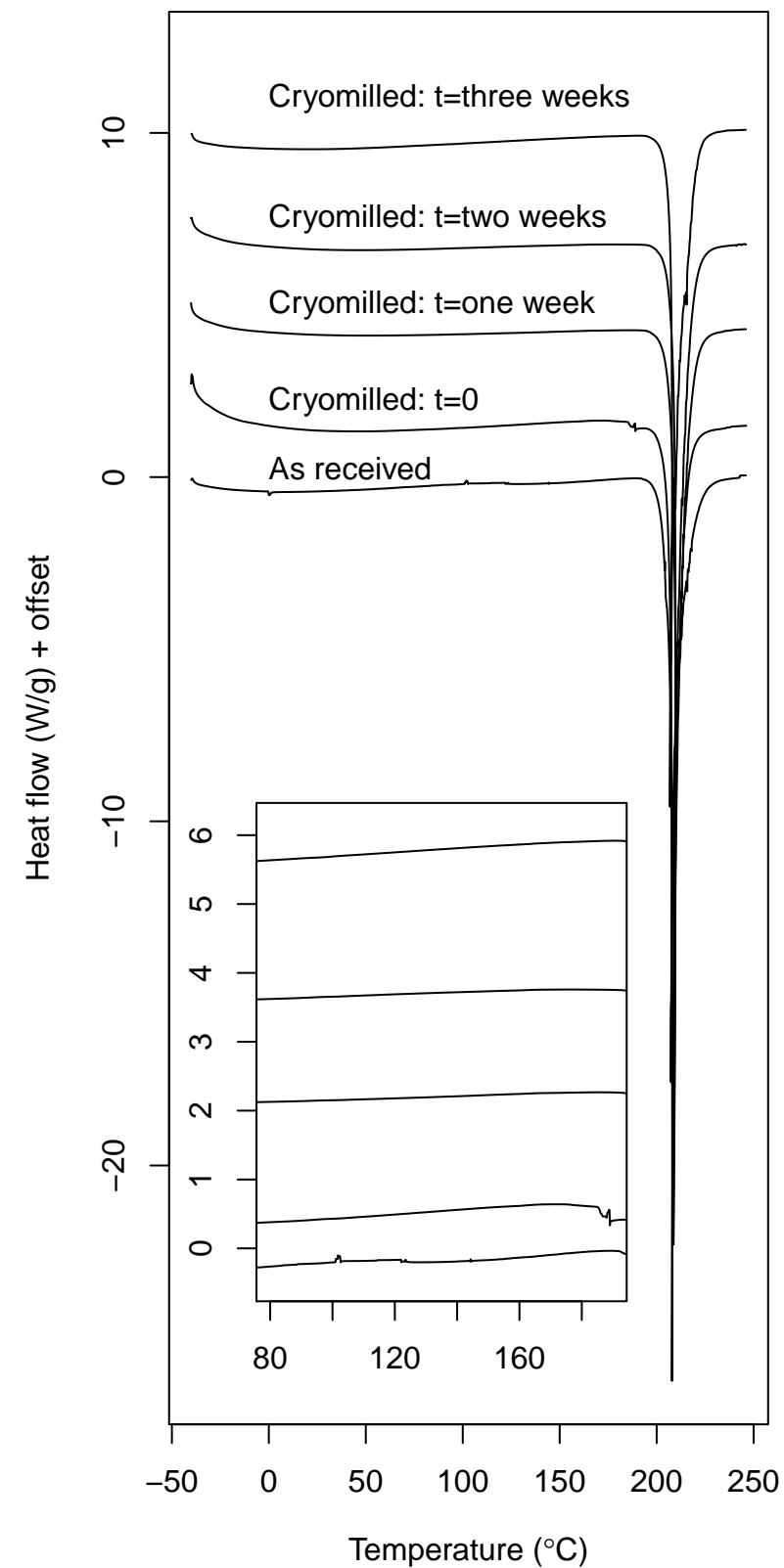

Supplement: Supplementary file 1 [file molecules-24-03990-s001.zip › SI_pack/Figure_2_DSC_single_components/Figure_2_single_components_DSC.pdf]

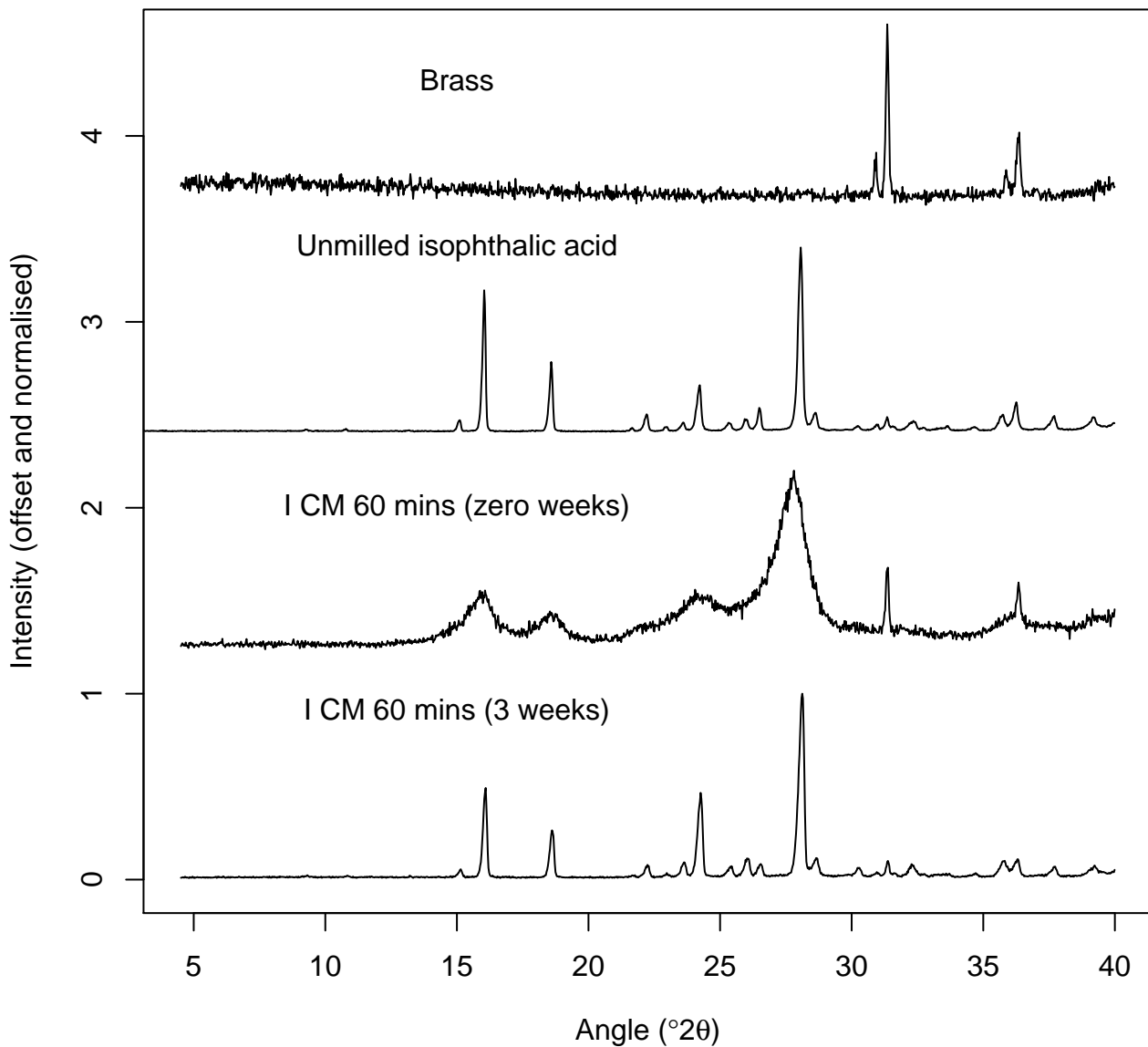

Supplement: Supplementary file 1 [file molecules-24-03990-s001.zip › SI_pack/Figure_3_XRPD_single_components/Data/Individual/Isophthalic acid/i new 22.pdf]

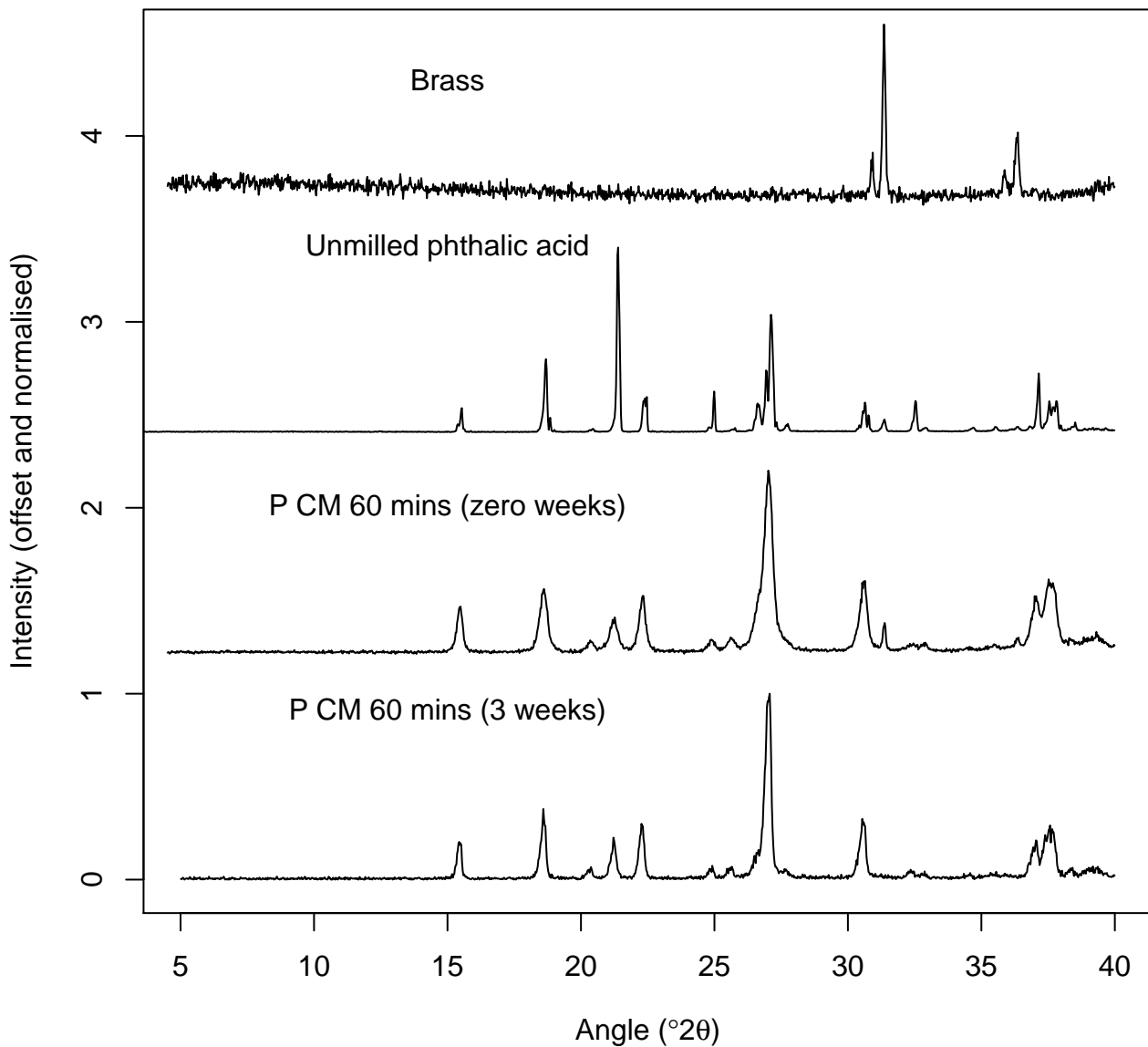

Supplement: Supplementary file 1 [file molecules-24-03990-s001.zip › SI_pack/Figure_3_XRPD_single_components/Data/Individual/Phthalic acid/P new 22.pdf]

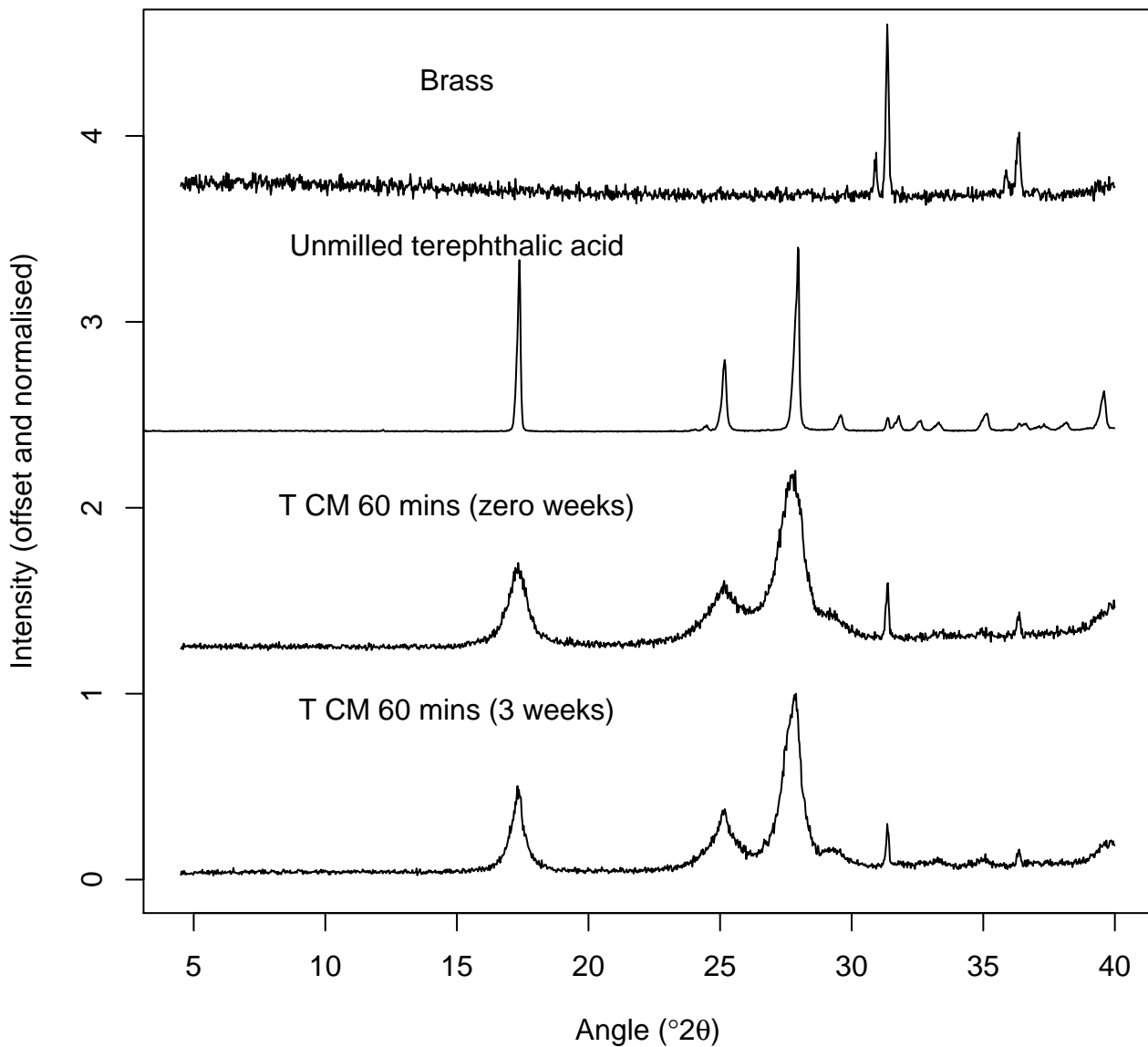

Supplement: Supplementary file 1 [file molecules-24-03990-s001.zip › SI_pack/Figure_3_XRPD_single_components/Data/Individual/Terephthalic acid/T new 22.pdf]

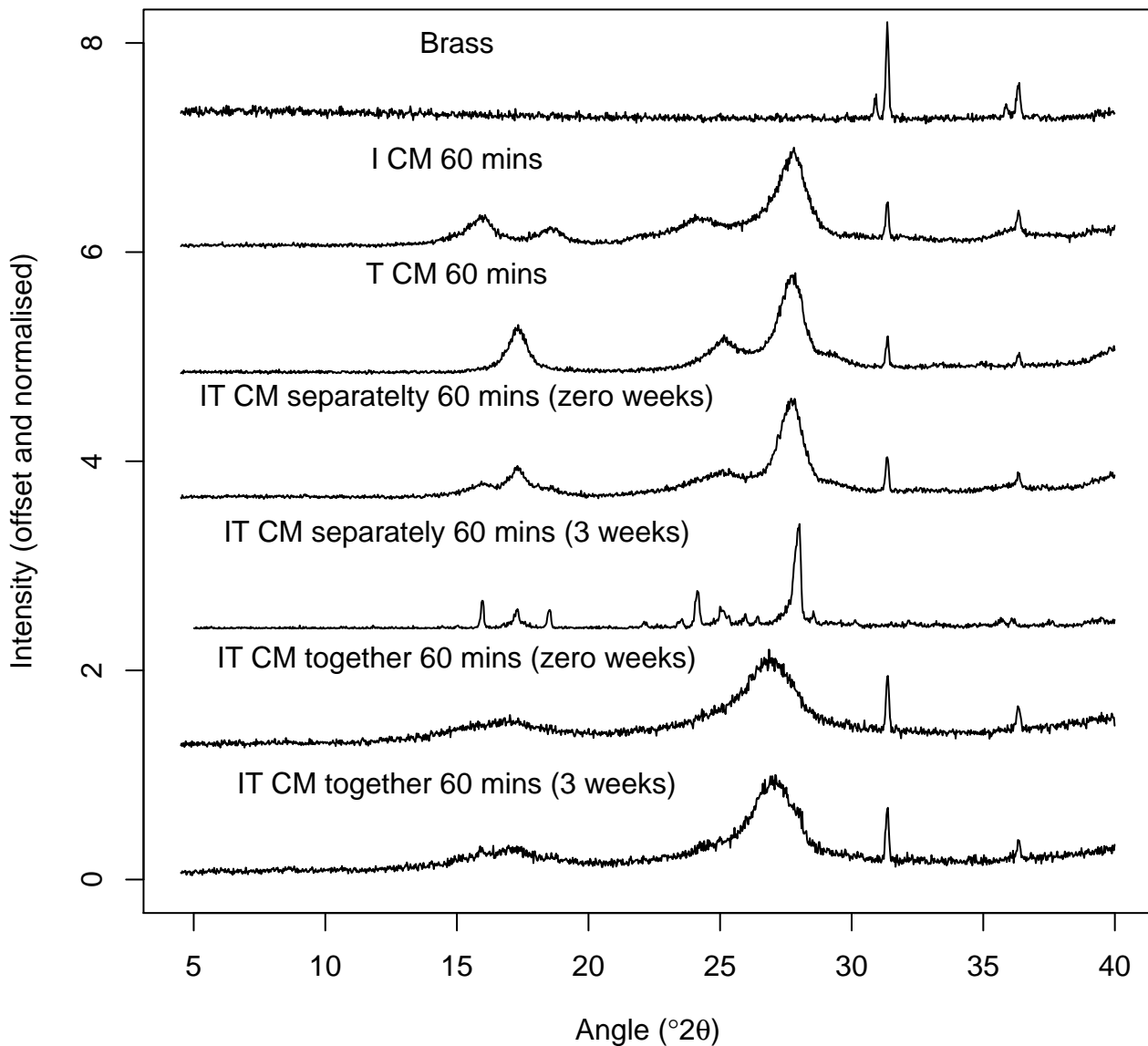

Supplement: Supplementary file 1 [file molecules-24-03990-s001.zip › SI_pack/Figure_3_XRPD_single_components/Data/Mixtures/IT/IT XRPD.pdf]

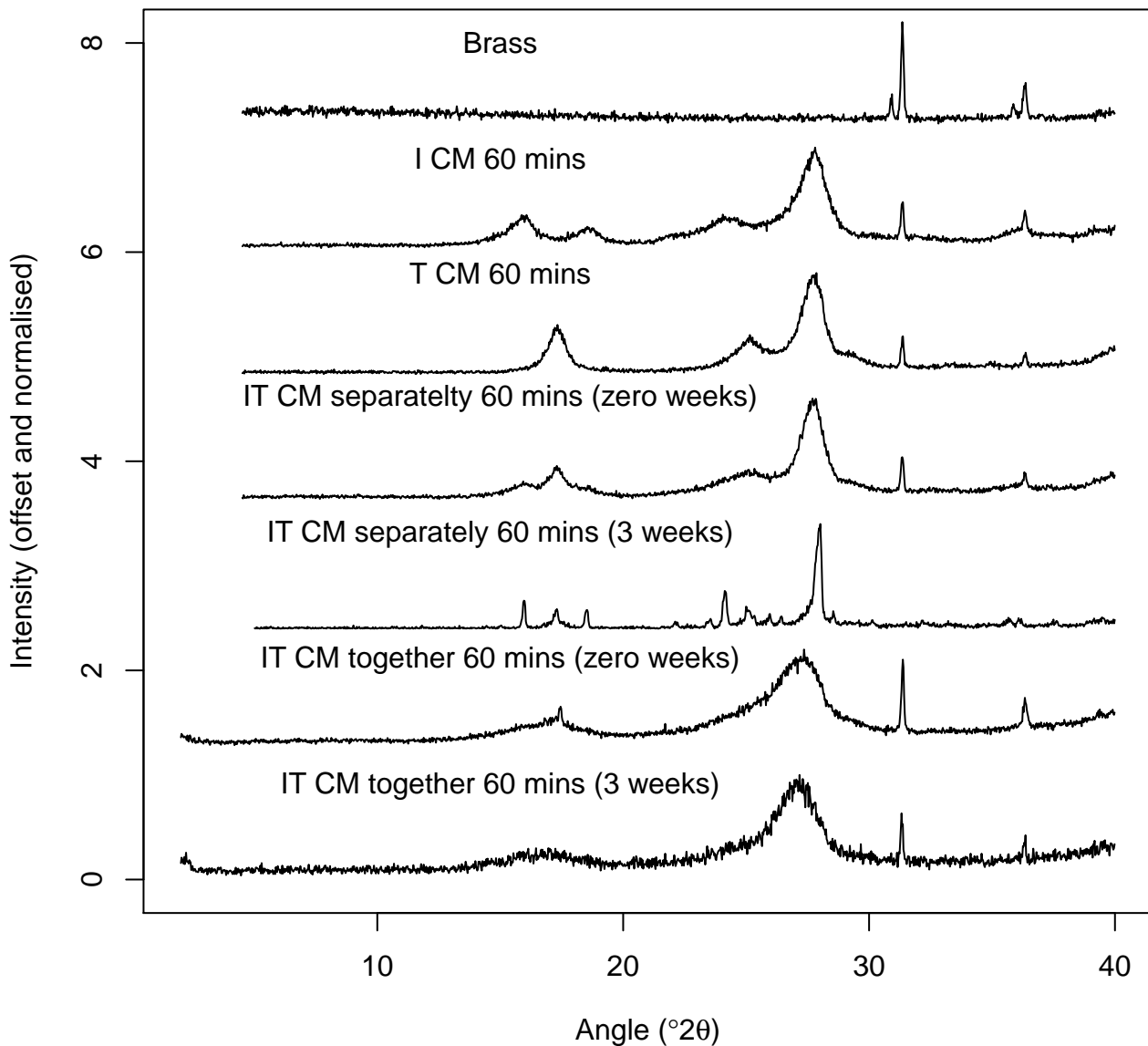

Supplement: Supplementary file 1 [file molecules-24-03990-s001.zip › SI_pack/Figure_3_XRPD_single_components/Data/Mixtures/IT/IT.pdf]

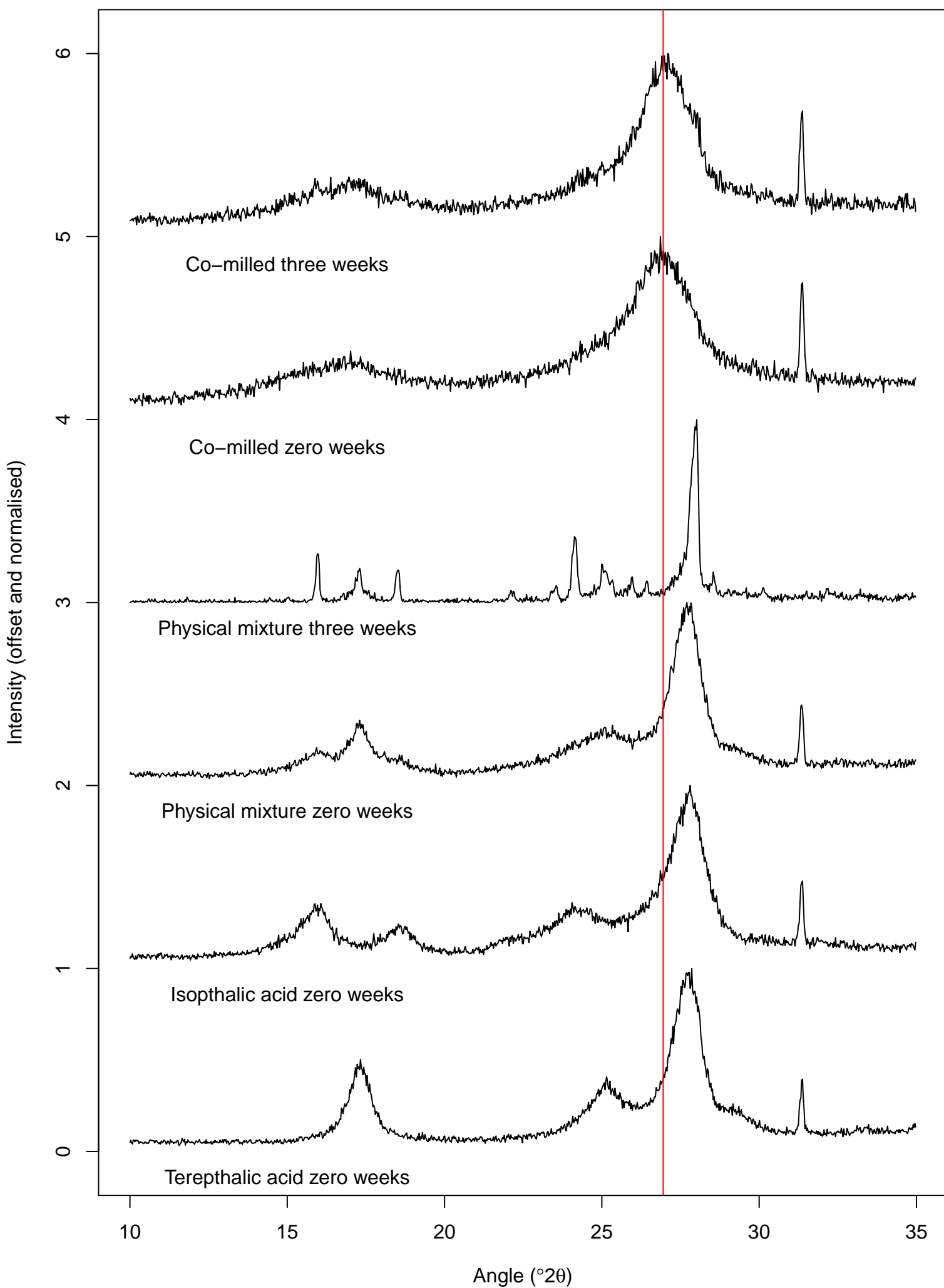

Supplement: Supplementary file 1 [file molecules-24-03990-s001.zip › SI_pack/Figure_3_XRPD_single_components/Data/Mixtures/IT/IT_XRPD_for_paper.pdf]

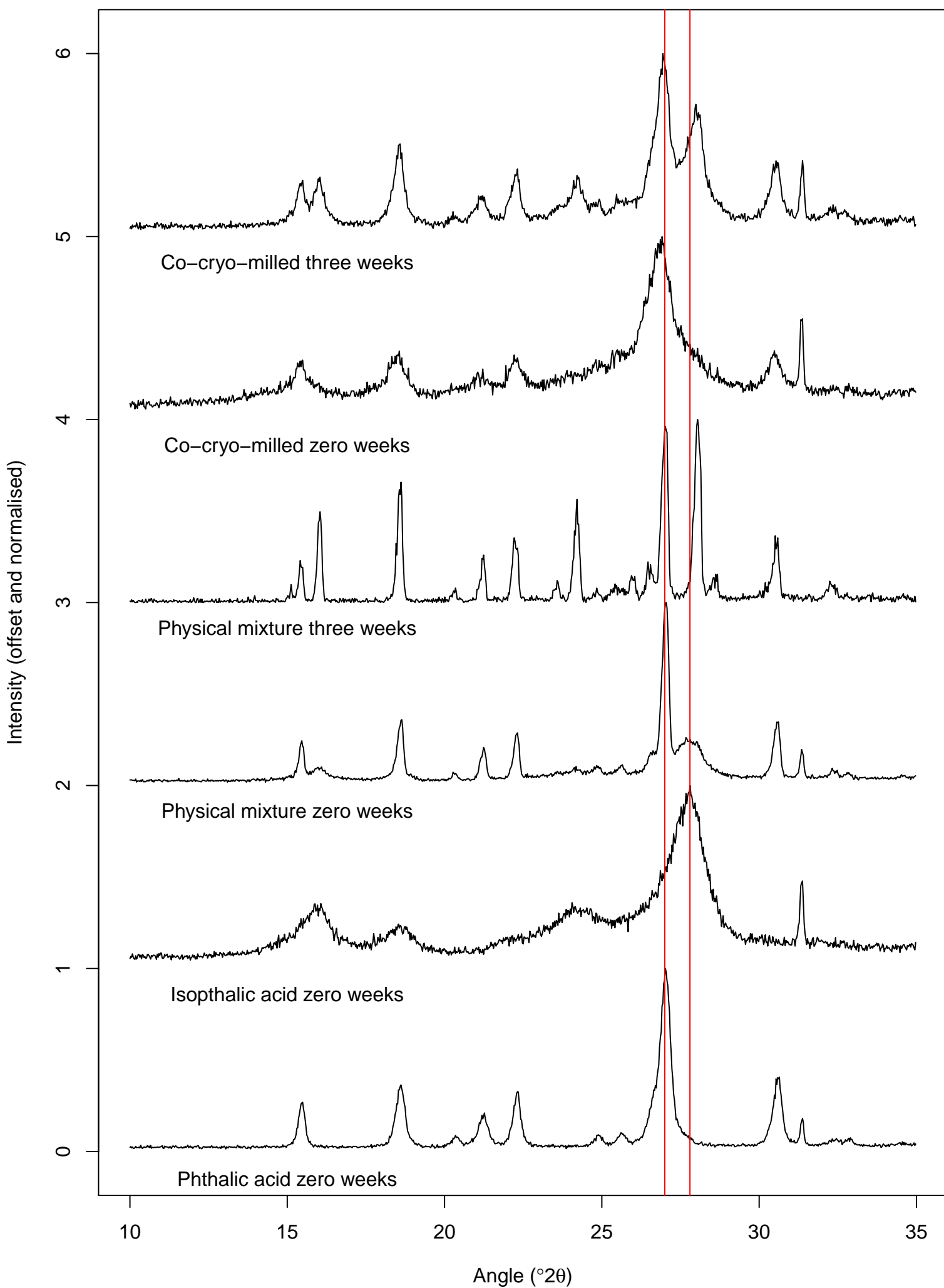

Supplement: Supplementary file 1 [file molecules-24-03990-s001.zip › SI_pack/Figure_3_XRPD_single_components/Data/Mixtures/PI/IP_XRPD_for_paper.pdf]

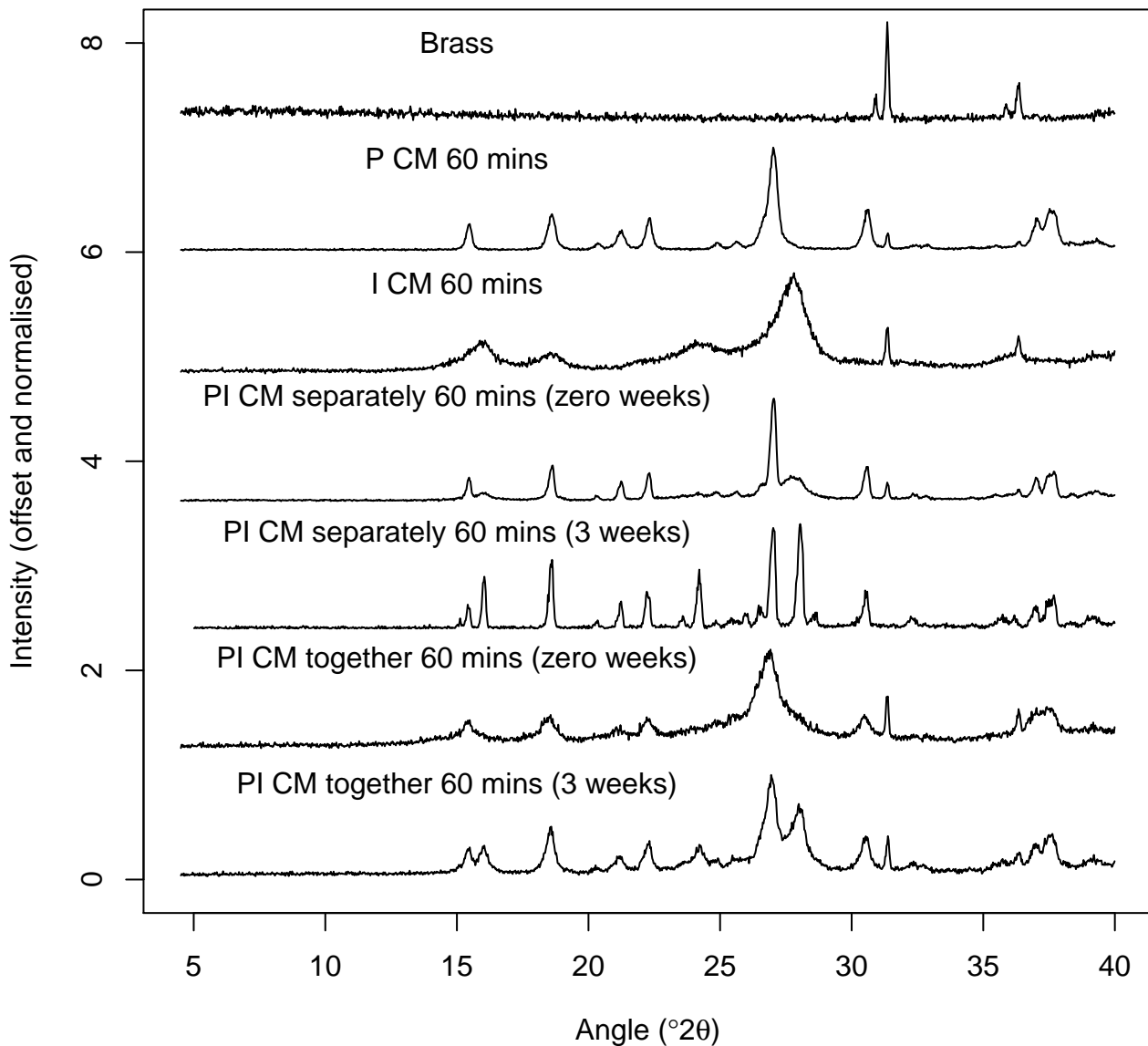

Supplement: Supplementary file 1 [file molecules-24-03990-s001.zip › SI_pack/Figure_3_XRPD_single_components/Data/Mixtures/PI/phthalic and isophthalic acid new.pdf]

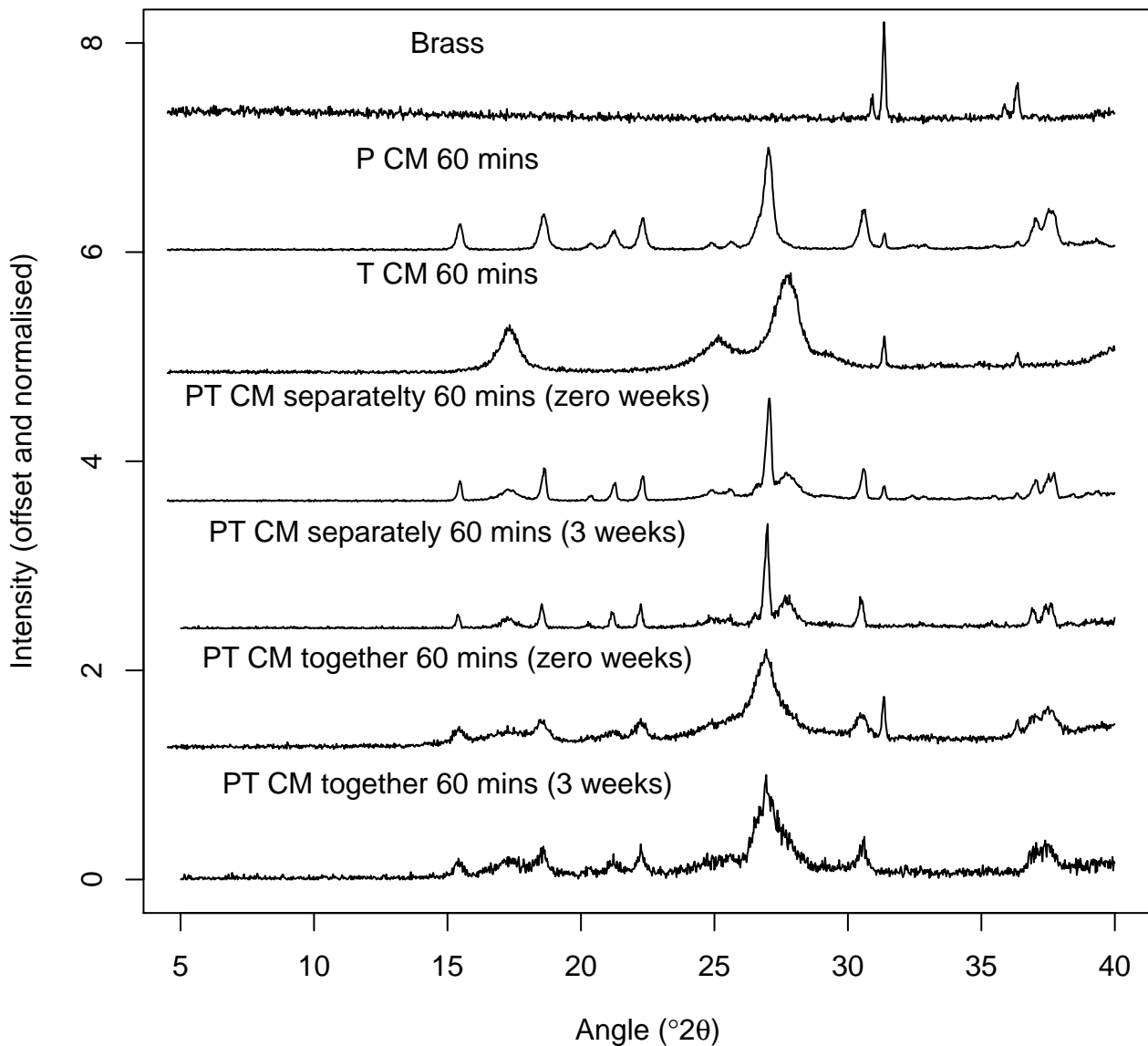

Supplement: Supplementary file 1 [file molecules-24-03990-s001.zip › SI_pack/Figure_3_XRPD_single_components/Data/Mixtures/PT/phthalic and terephthalic acid new22.pdf]

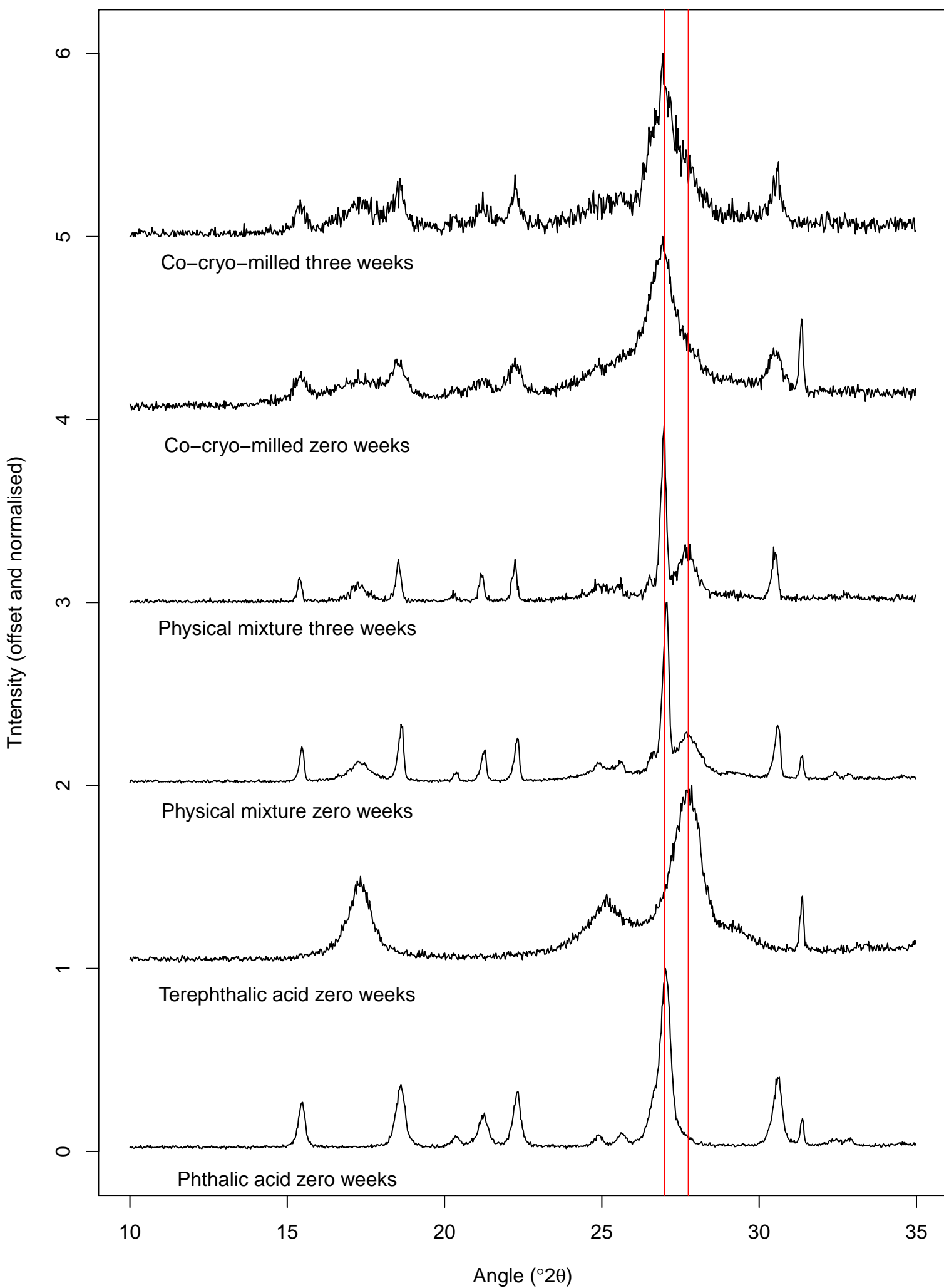

Supplement: Supplementary file 1 [file molecules-24-03990-s001.zip › SI_pack/Figure_3_XRPD_single_components/Data/Mixtures/PT/TP_XRPD_for_paper.pdf]

**a) Terephthalic acid**

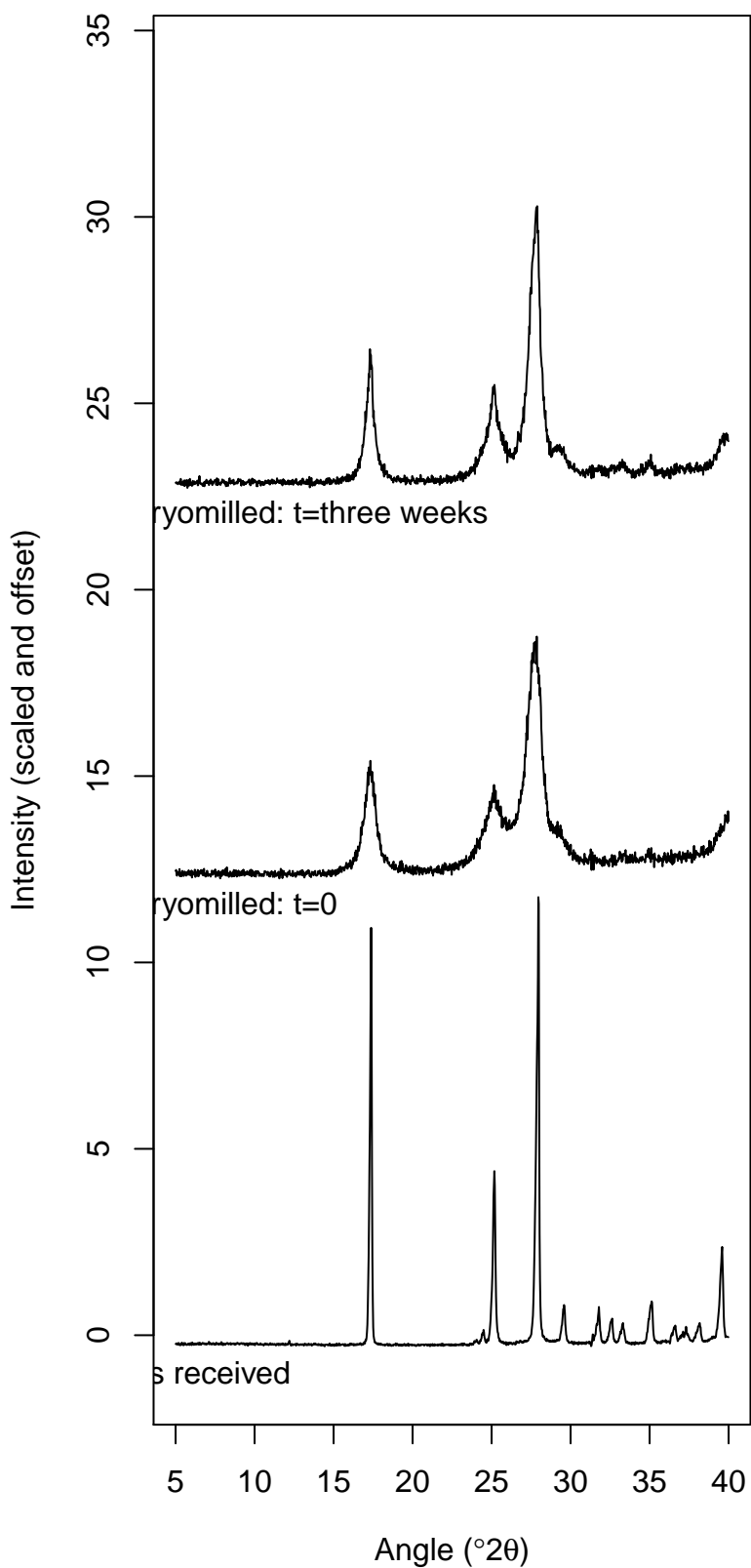

**b) Isophthalic acid**

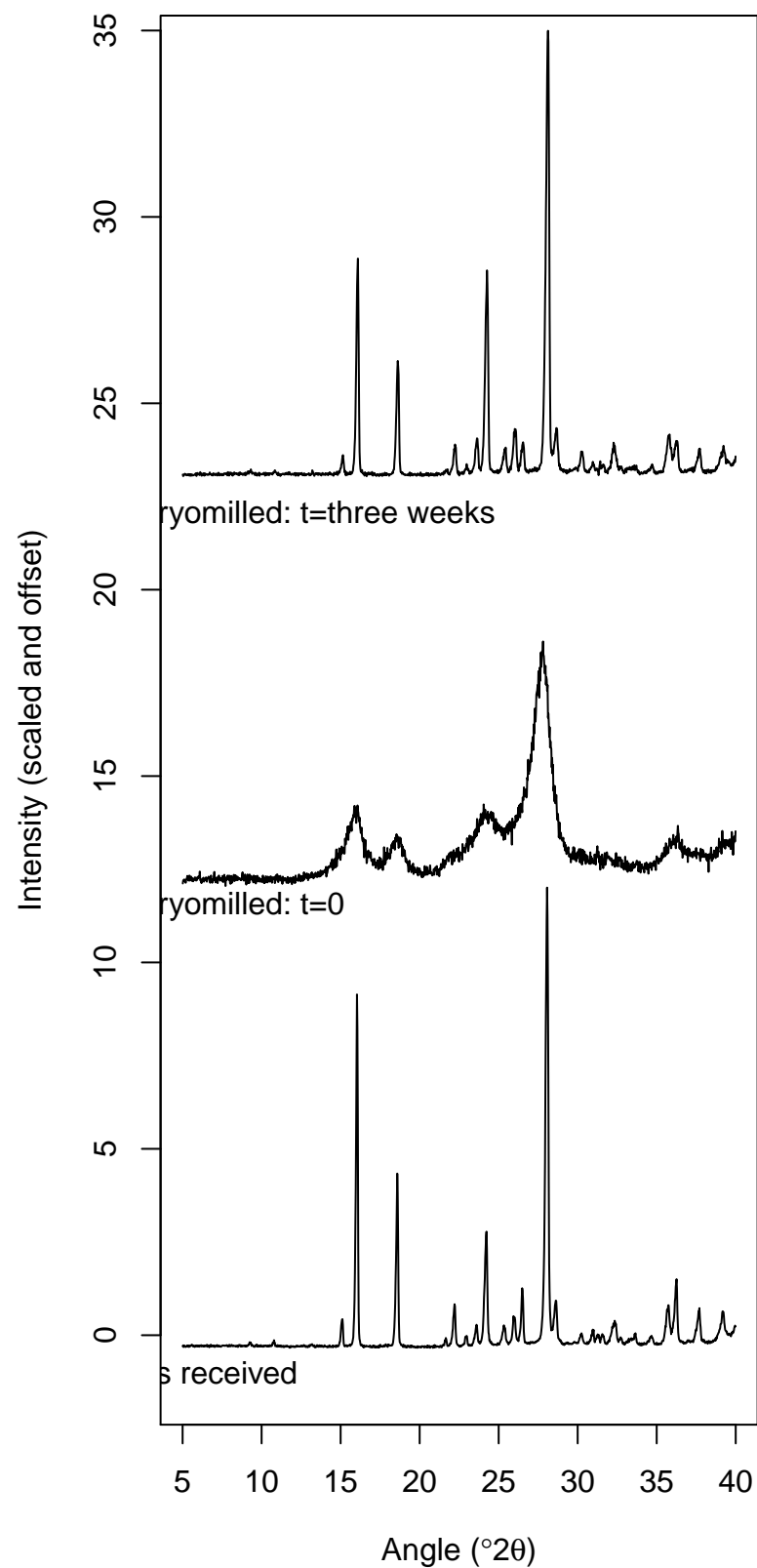

**c) Phthalic acid**

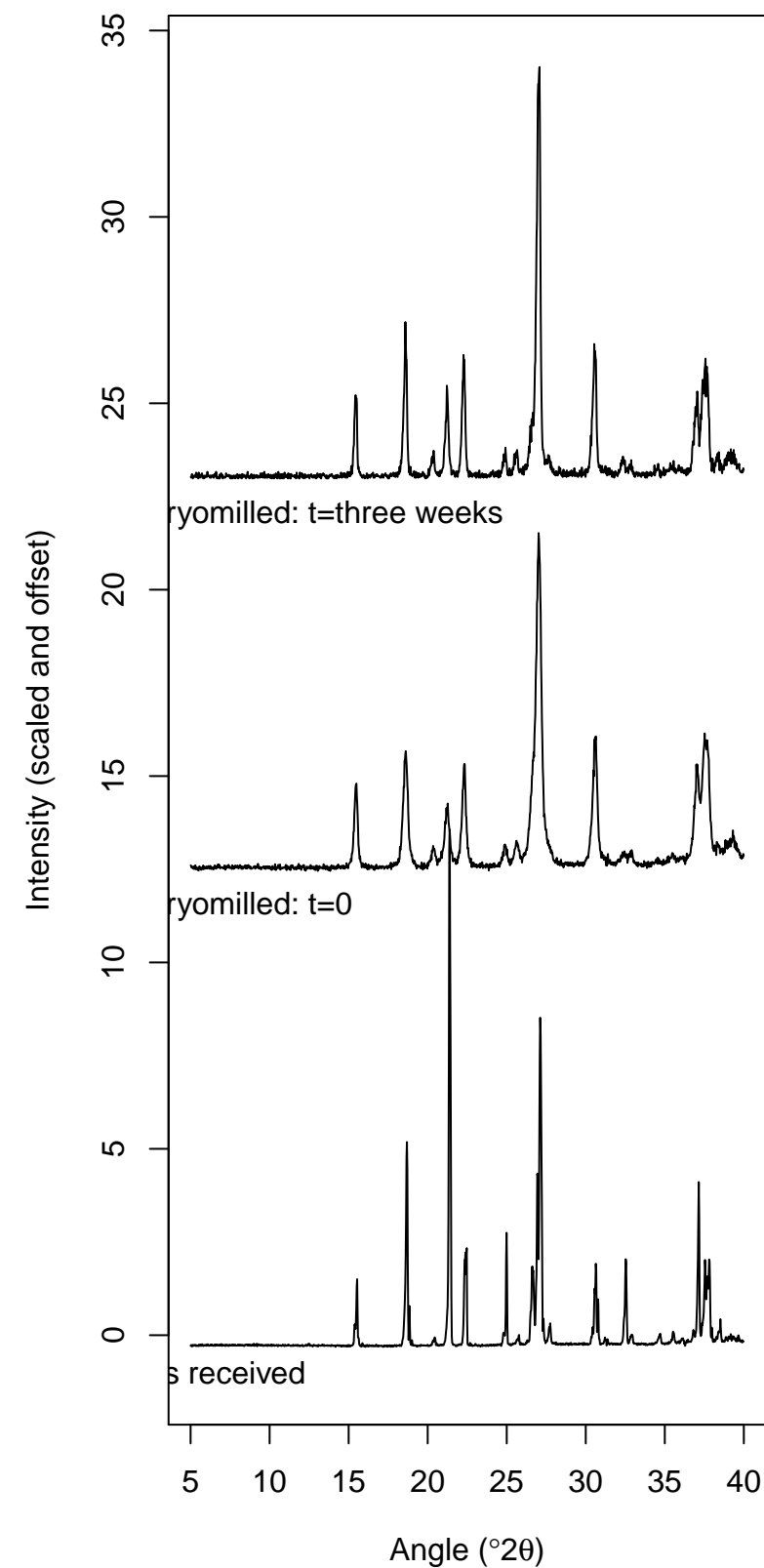

Supplement: Supplementary file 1 [file molecules-24-03990-s001.zip › SI_pack/Figure_3_XRPD_single_components/Figure_3_single_components_XRPD.pdf]

**a) Terephthalic acid**

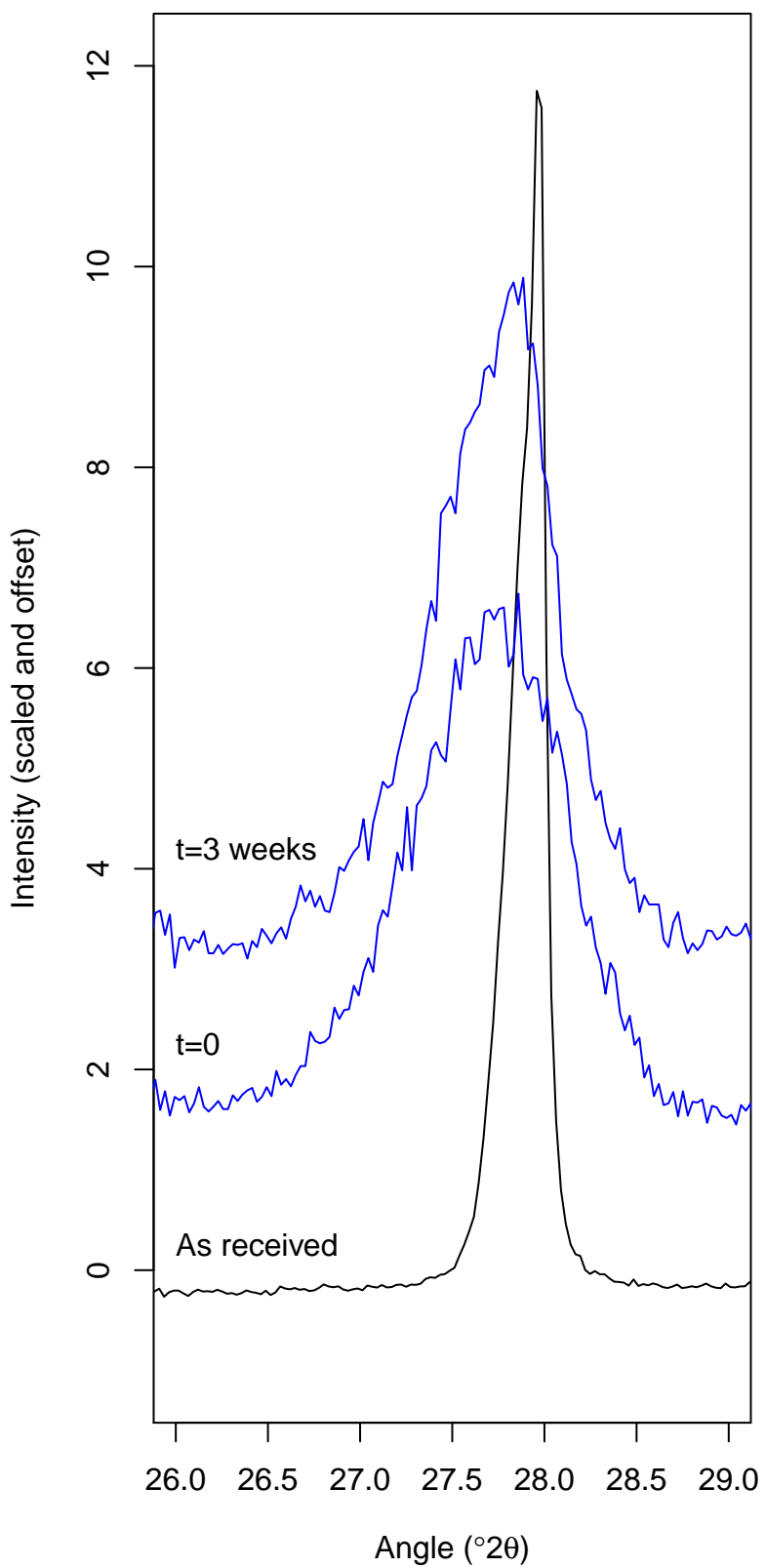

**b) Isophthalic acid**

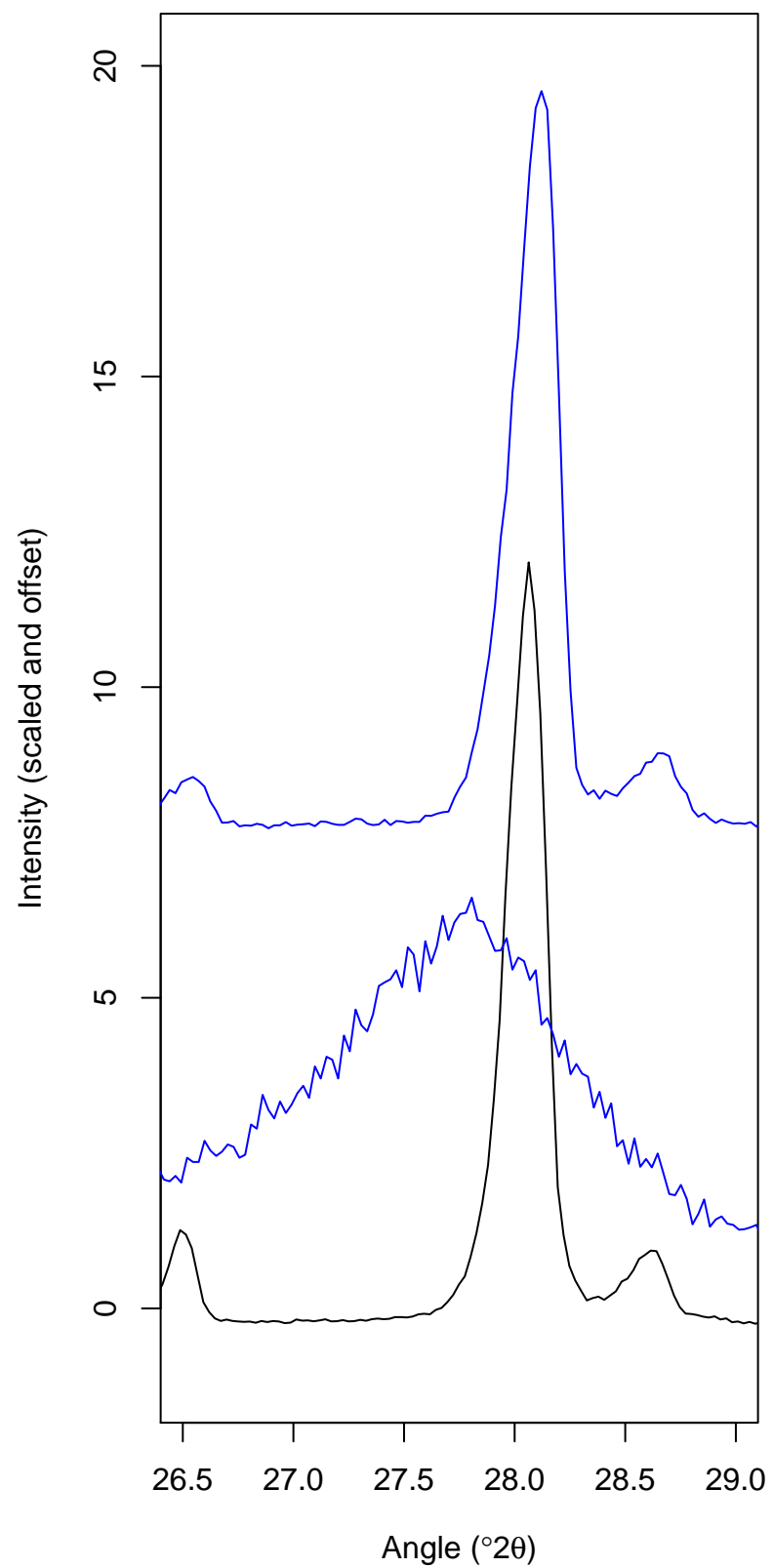

**c) Phthalic acid**

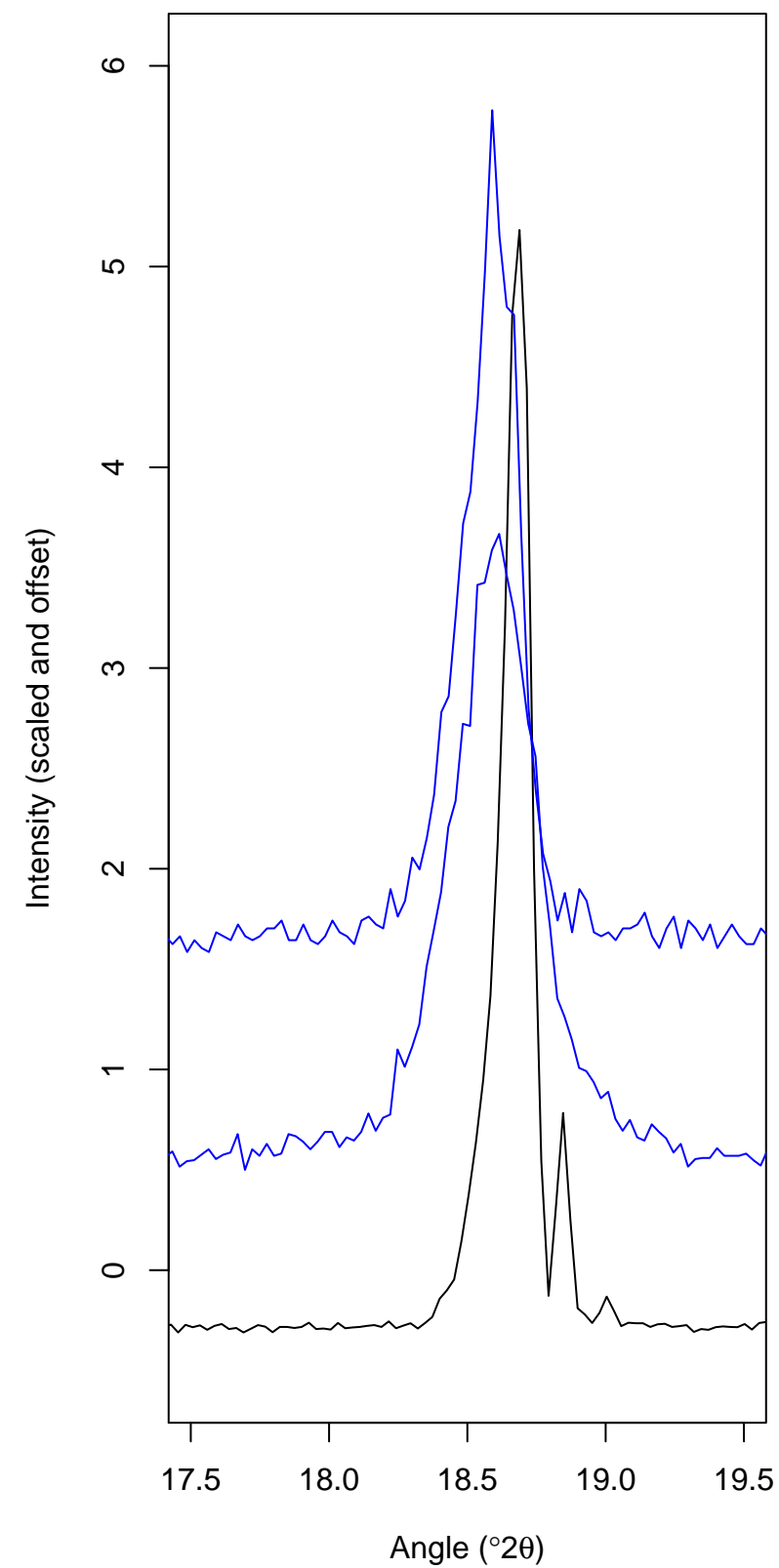

Supplement: Supplementary file 1 [file molecules-24-03990-s001.zip › SI_pack/Figure_3_XRPD_single_components/Figure_SI_1_Single_components_XRPD_peak_shifts.pdf]

**a) Terephthalic and isophthalic**   **b) Isophthalic and phthalic**   **c) Terephthalic and phthalic**

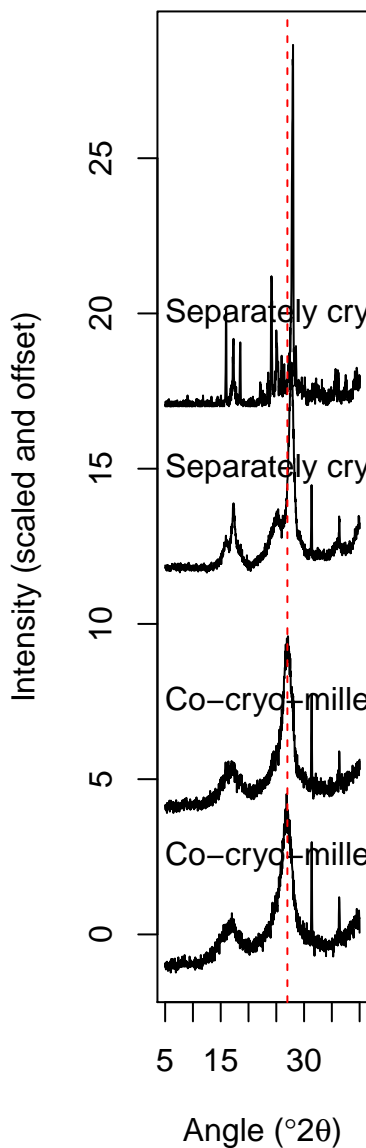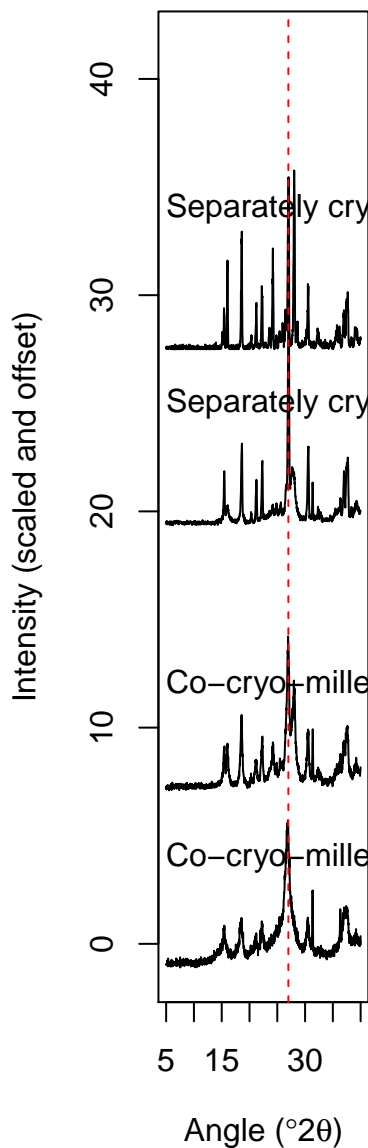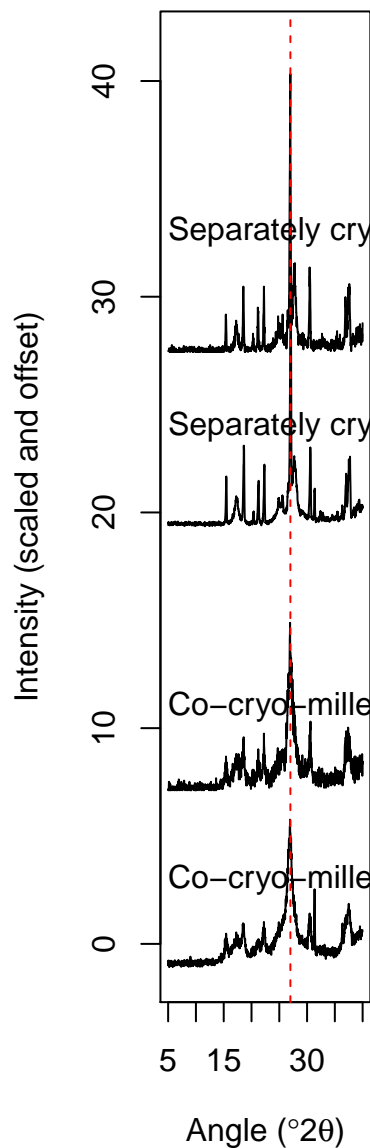

Supplement: Supplementary file 1 [file molecules-24-03990-s001.zip › SI_pack/Figure_3_XRPD_single_components/Rplots.pdf]

**a) Terephthalic acid**

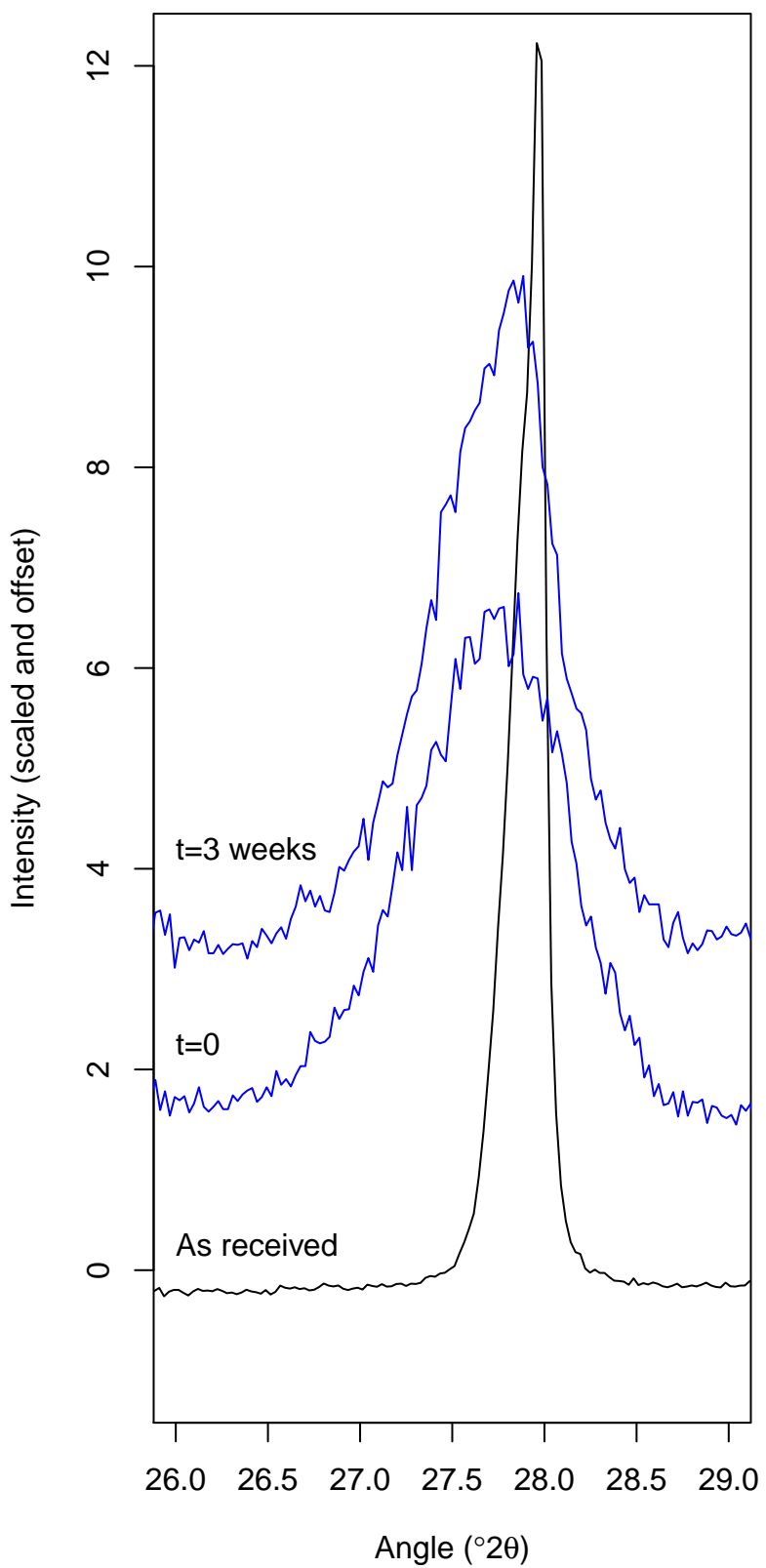

**b) Isophthalic acid**

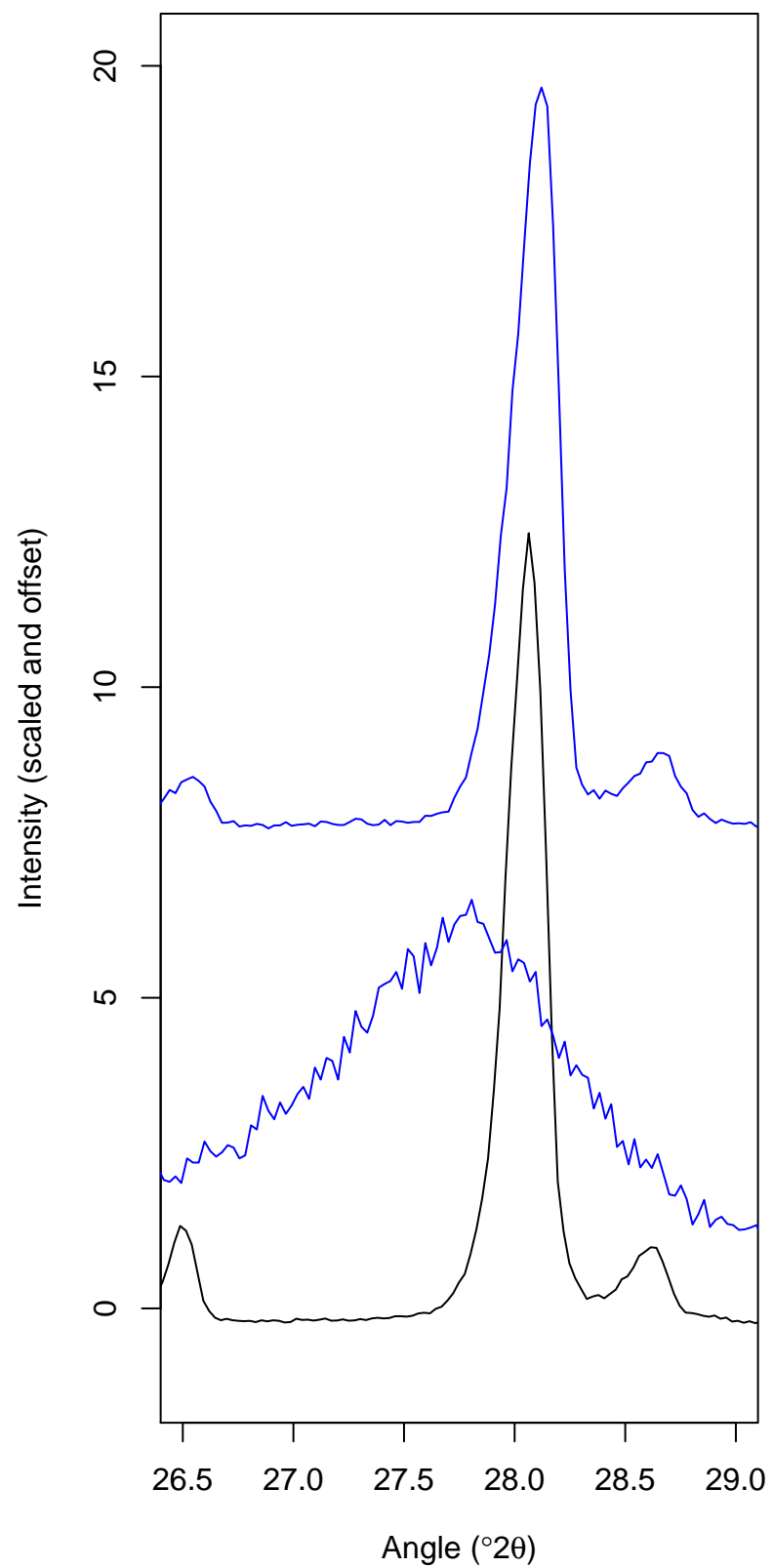

**c) Phthalic acid**

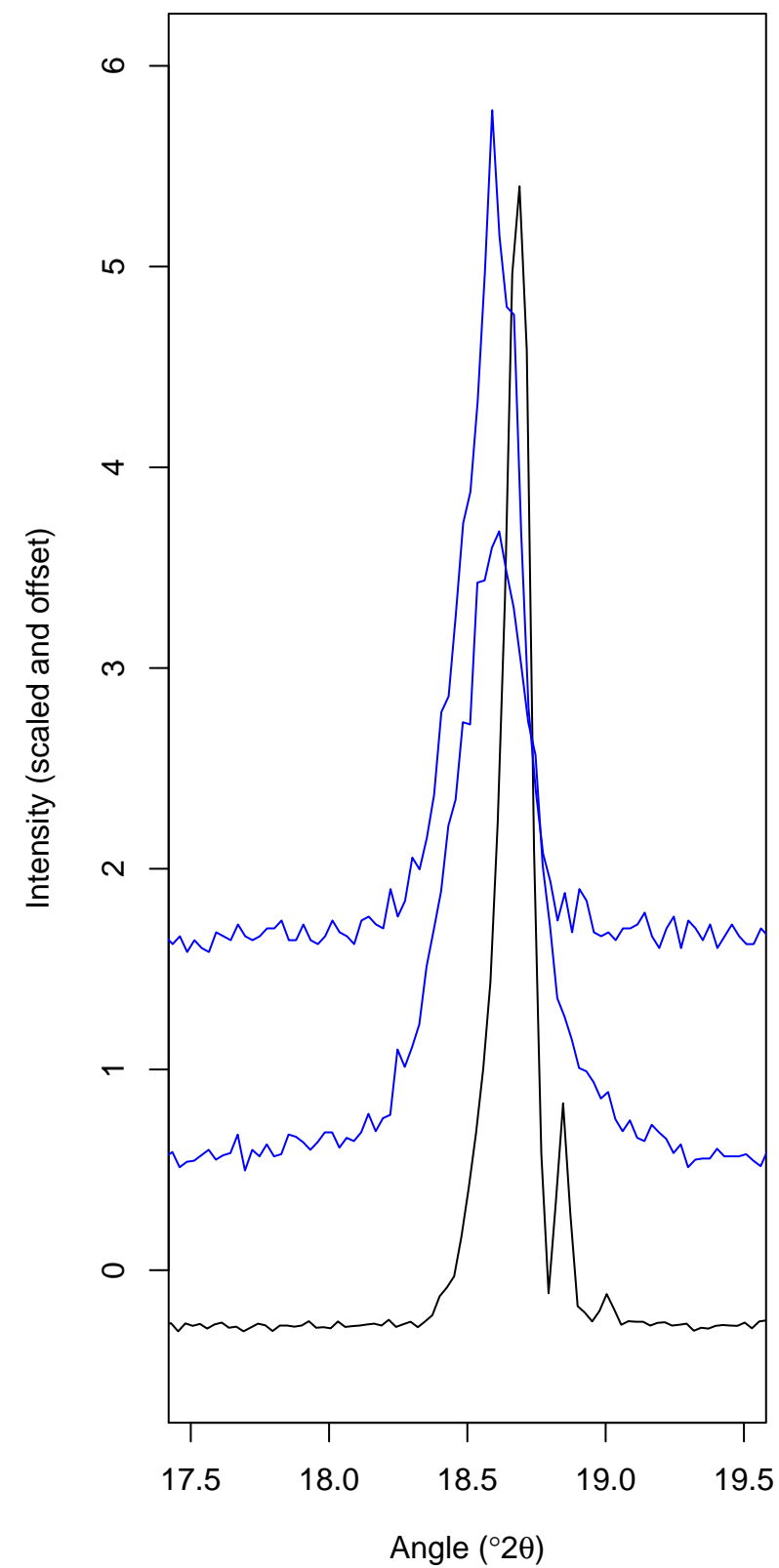

Supplement: Supplementary file 1 [file molecules-24-03990-s001.zip › SI_pack/Figure_3_XRPD_single_components/Single_components_XRPD_peak_shifts.pdf]

**isophthalic acid cryomilled for 60 mins after 3 weeks**

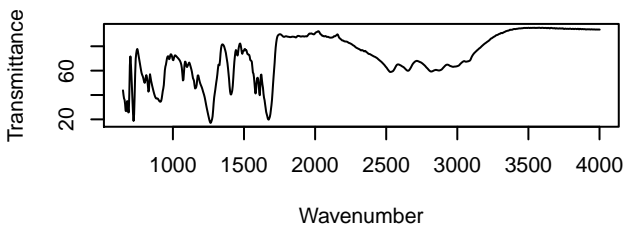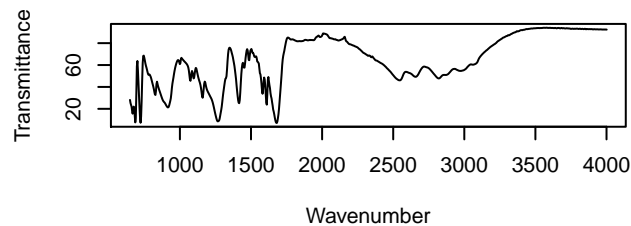

**isophthalic acid as received**

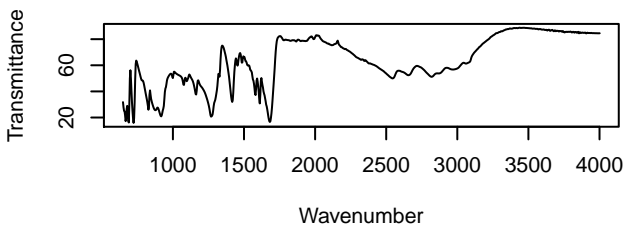

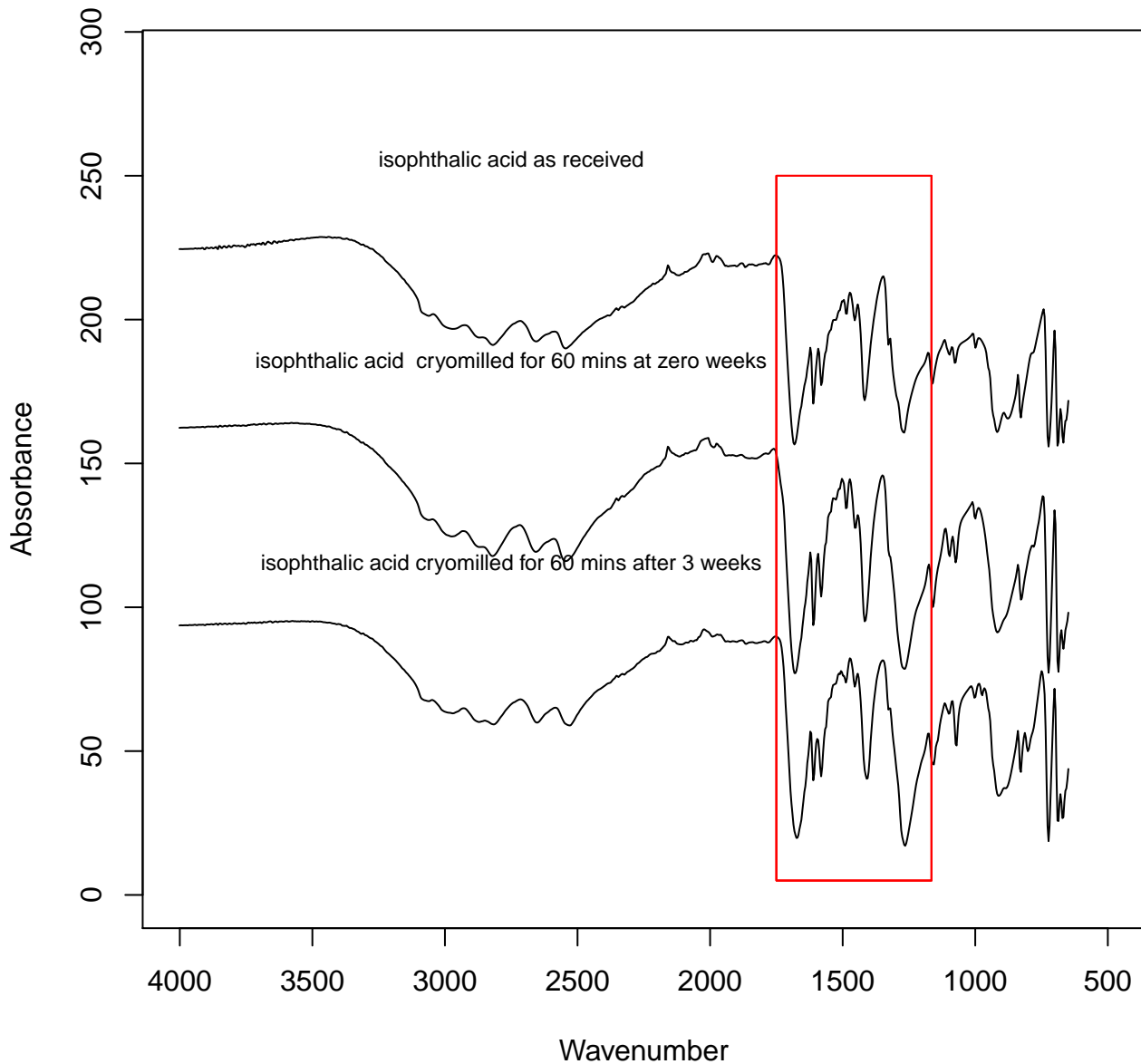

Supplement: Supplementary file 1 [file molecules-24-03990-s001.zip › SI_pack/Figure_4_FTIR_single_components/Data/Isophthalic acid/Isophthalic acid.pdf]

**a) Terephthalic acid**

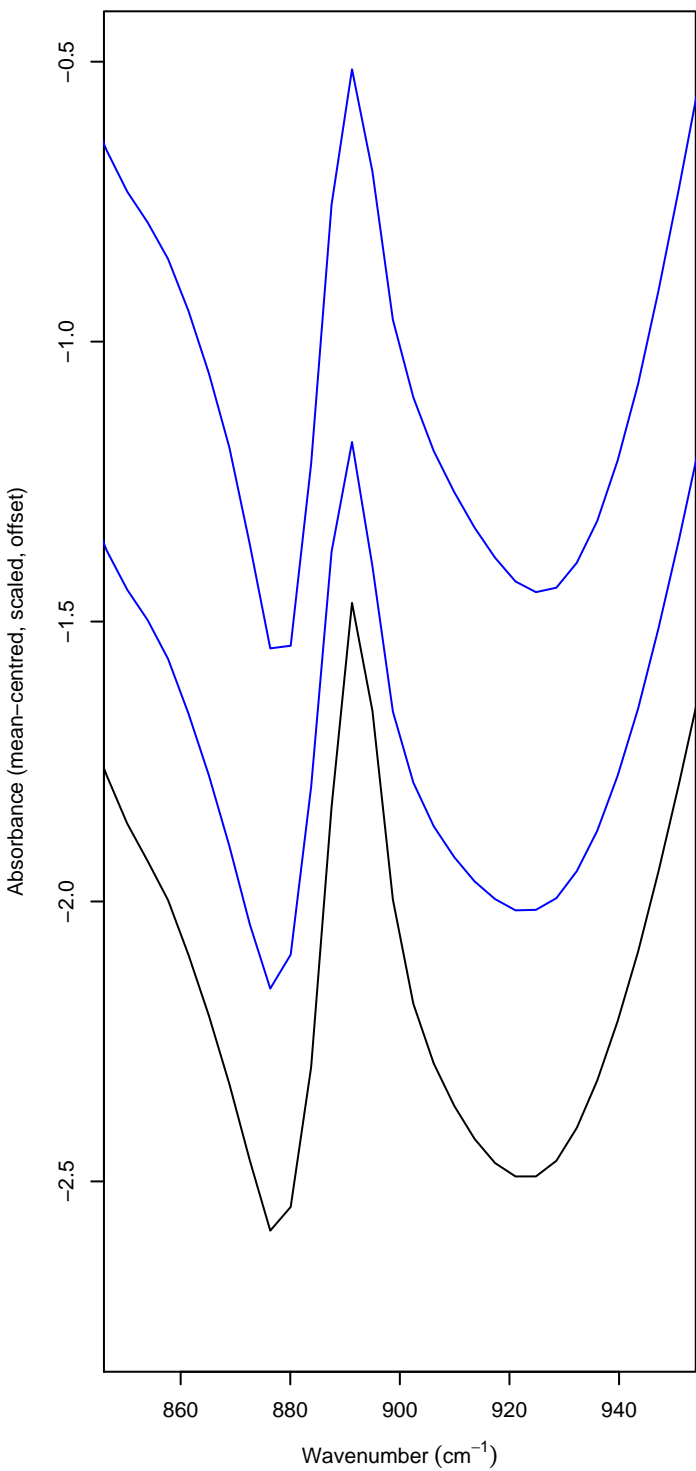

**b) Isophthalic acid**

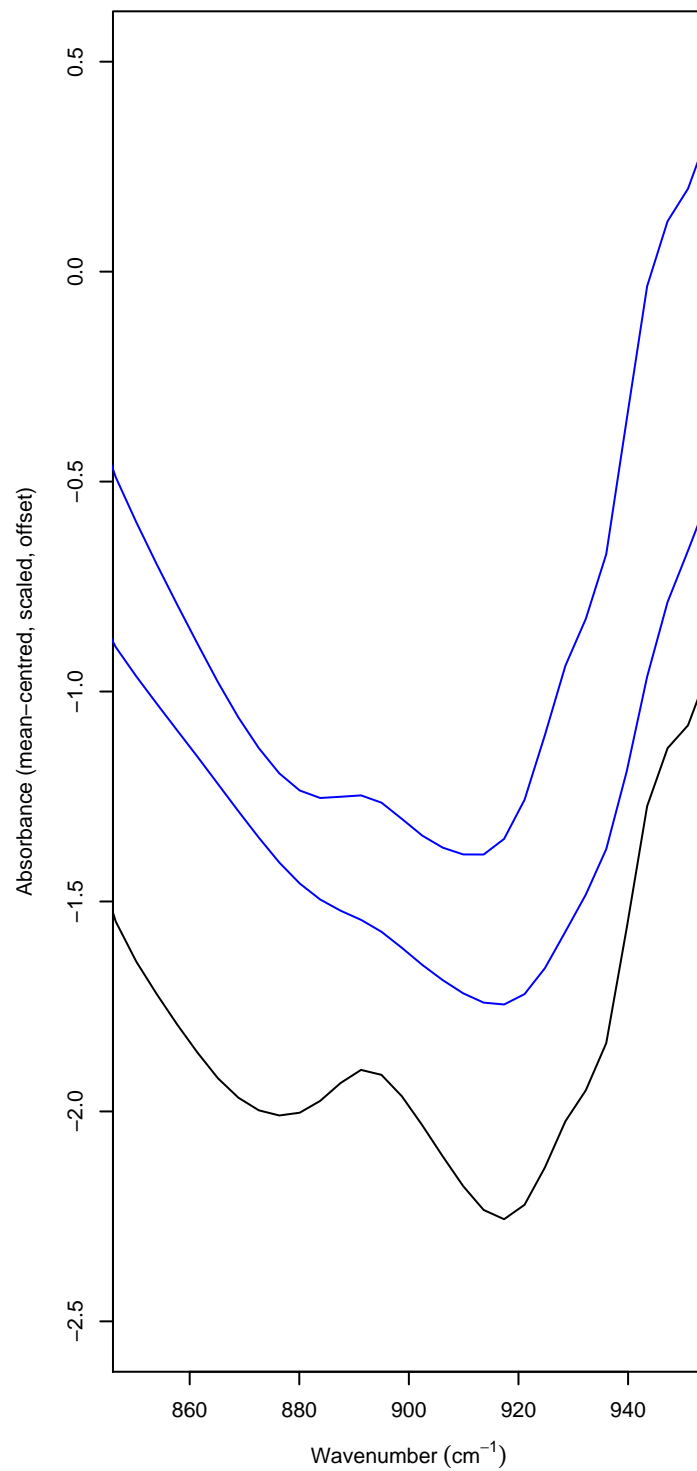

**c) Phthalic acid**

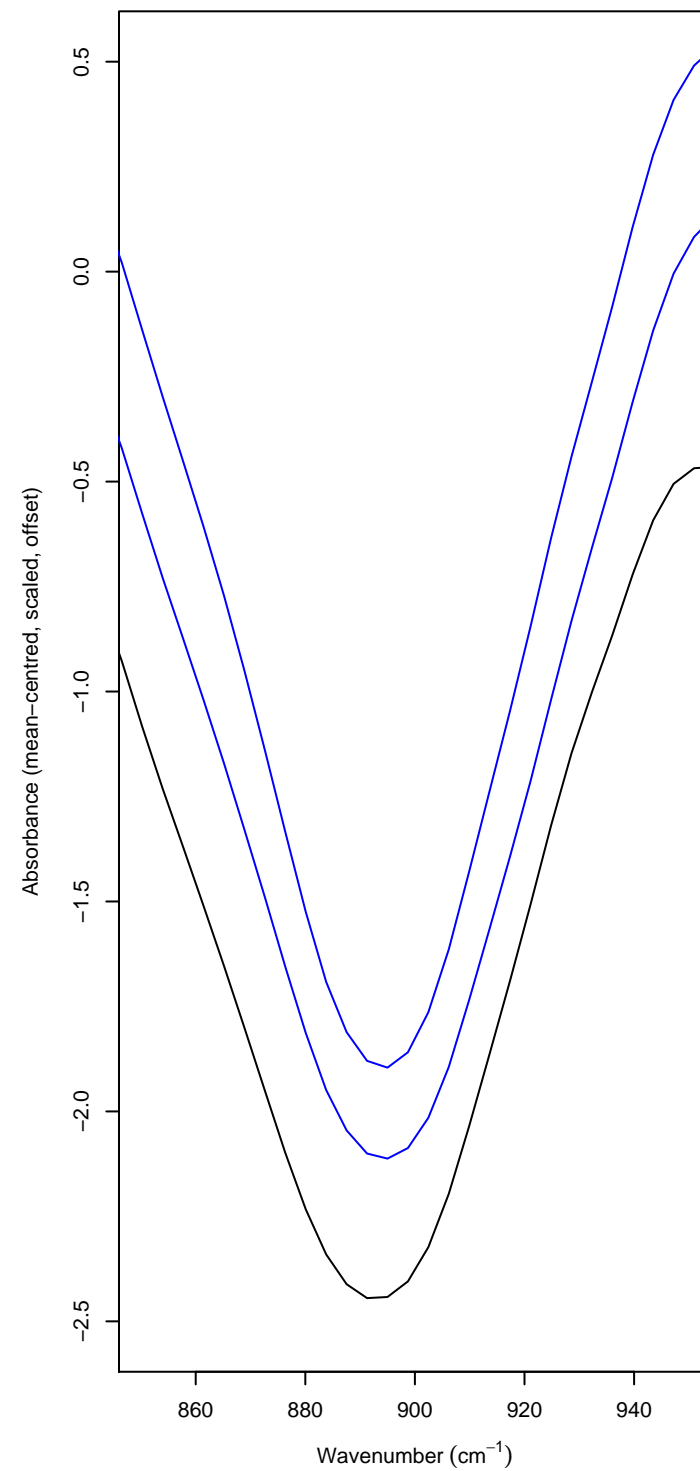

Supplement: Supplementary file 1 [file molecules-24-03990-s001.zip › SI_pack/Figure_4_FTIR_single_components/Figure_2SI_FTIR_overlay_plots.pdf]

**a) Terephthalic + isophthalic acid**

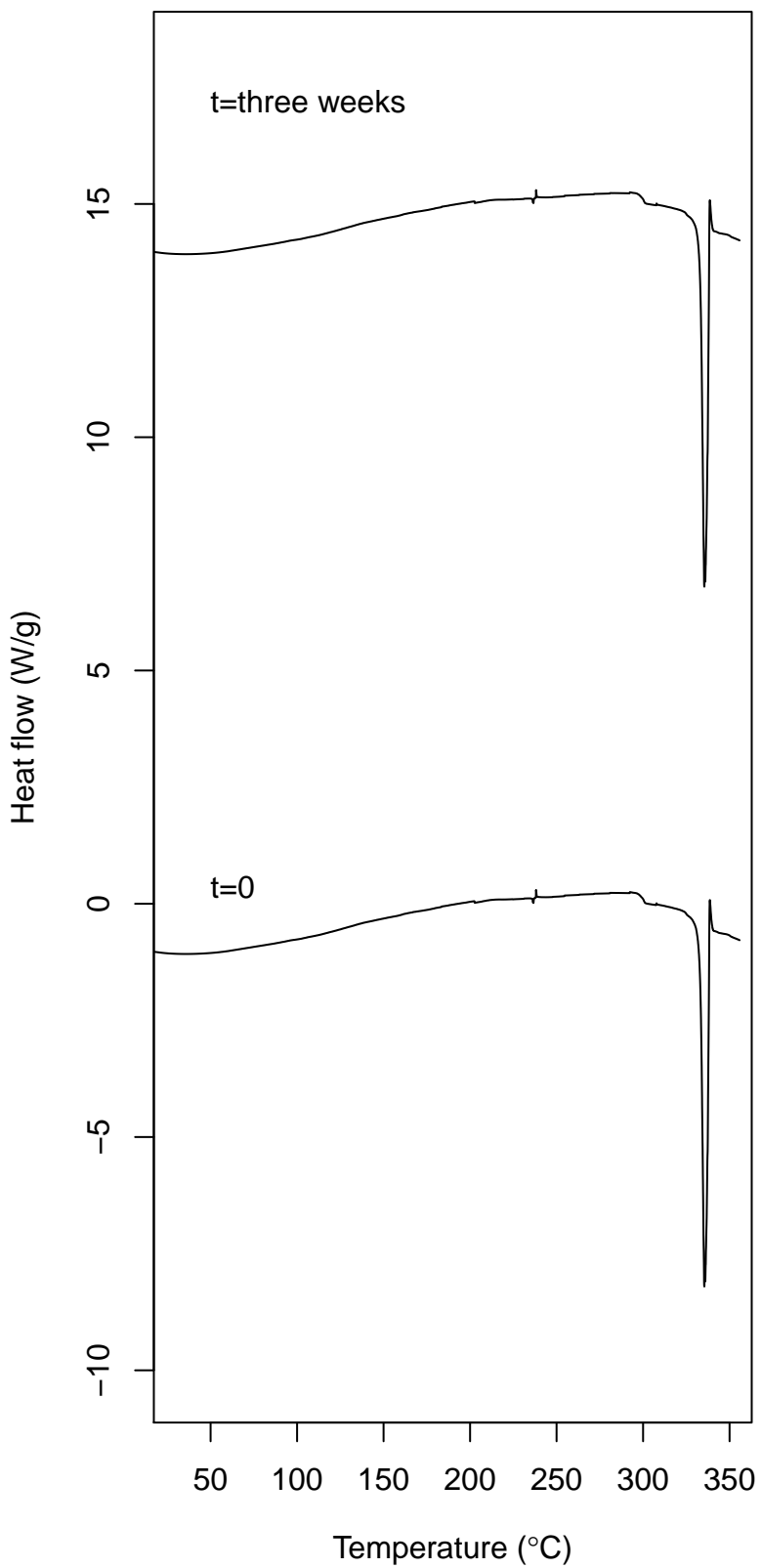

**b) Terephthalic + phthalic acid**

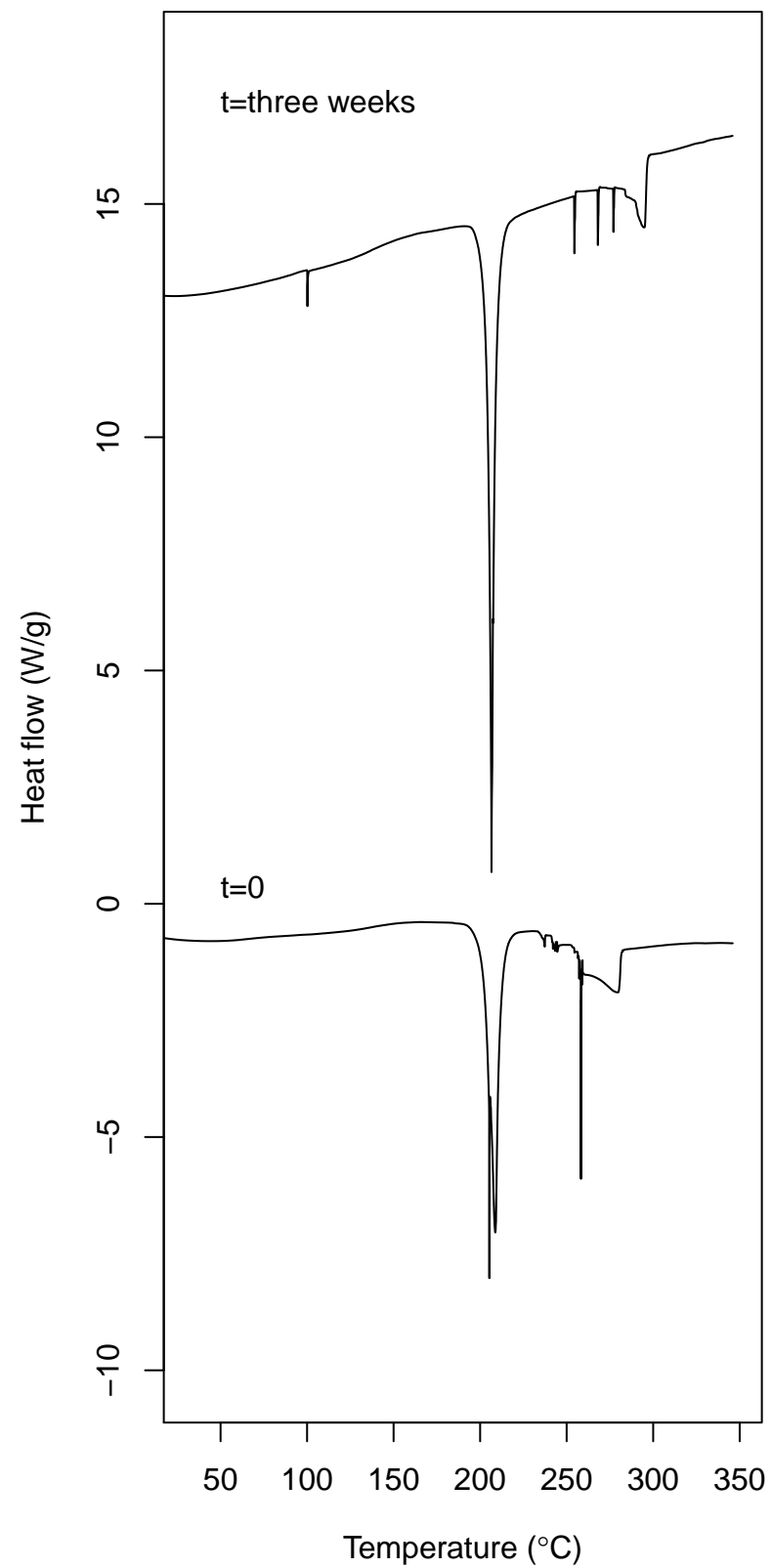

**c) Isophthalic + phthalic acid**

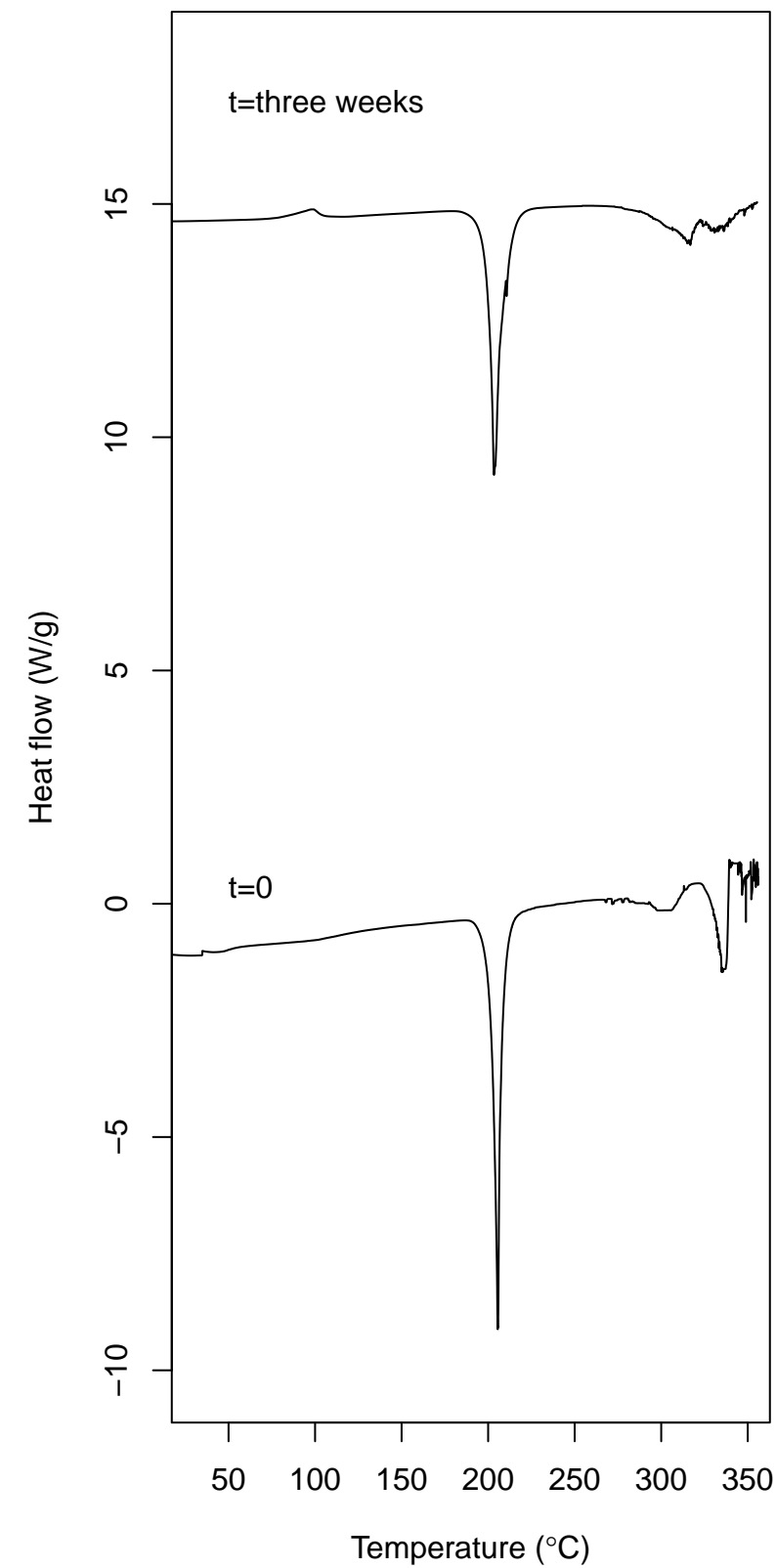

Supplement: Supplementary file 1 [file molecules-24-03990-s001.zip › SI_pack/Figure_5_DSC_two_components/Data/Two_components_DSC.pdf]

**a) Terephthalic + isophthalic acid**

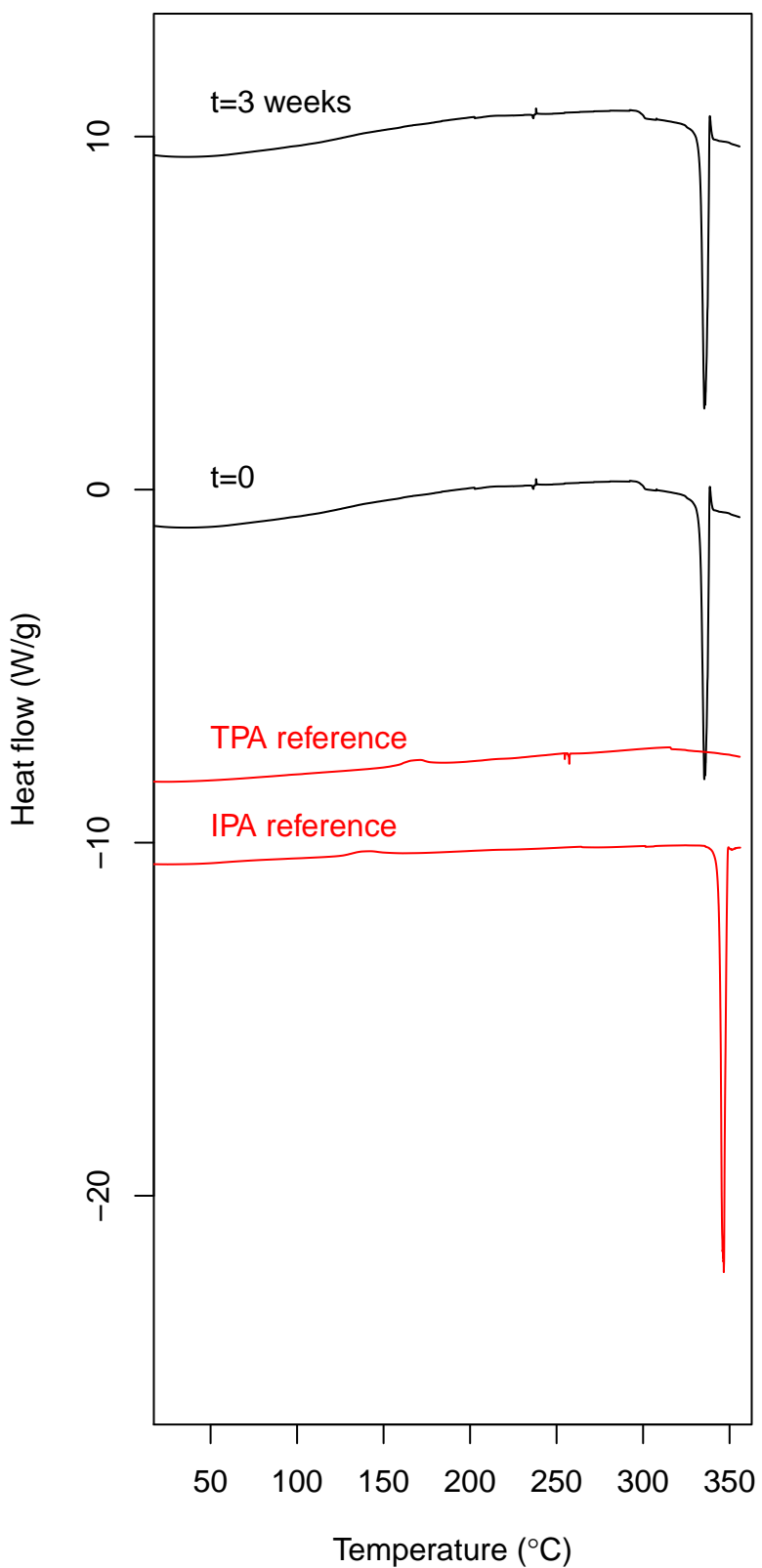

**b) Terephthalic + phthalic acid**

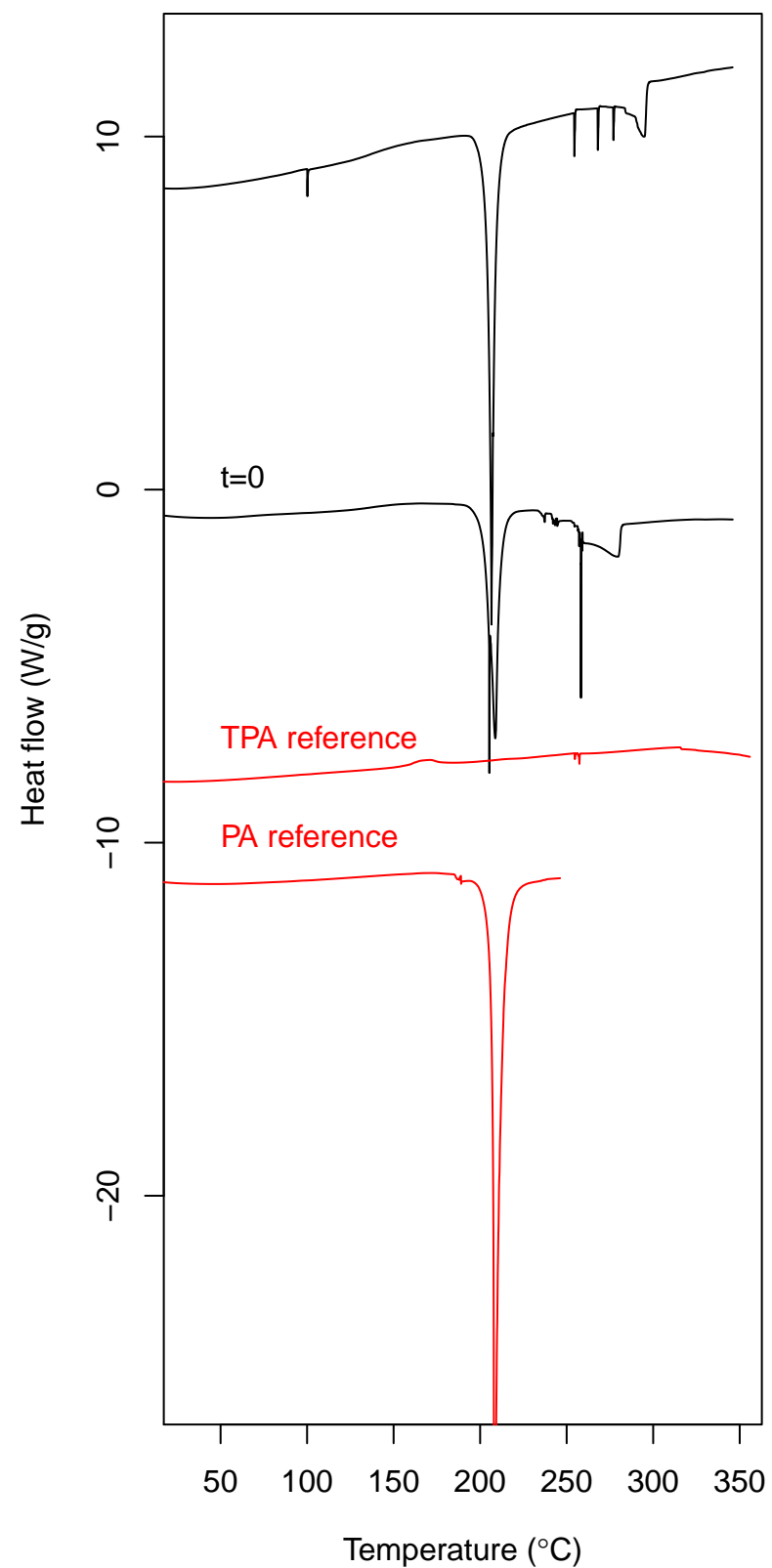

**c) Isophthalic + phthalic acid**

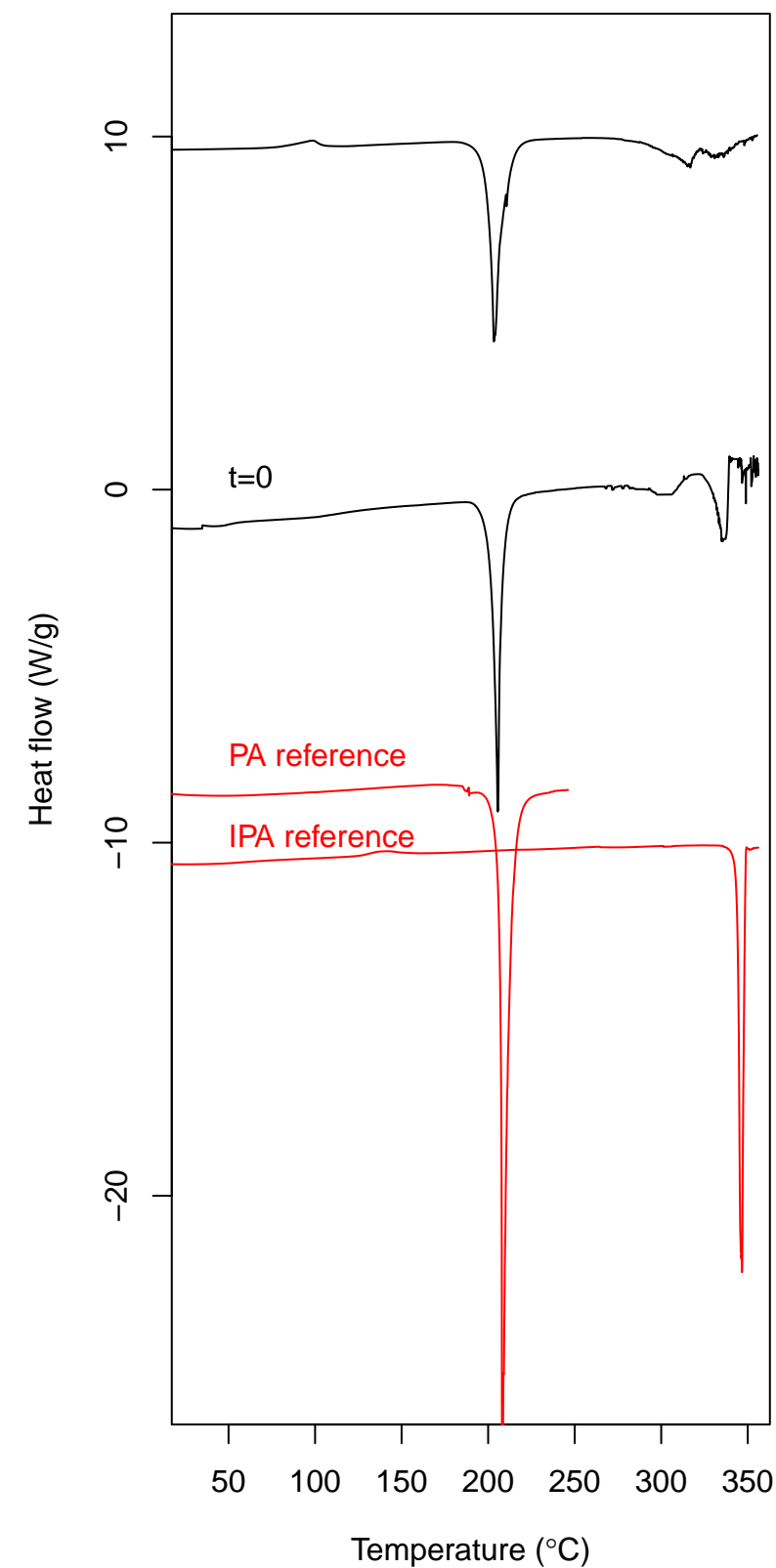

Supplement: Supplementary file 1 [file molecules-24-03990-s001.zip › SI_pack/Figure_5_DSC_two_components/Figure_5_two_components_DSC.pdf]

**a) Terephthalic + isophthalic acid**

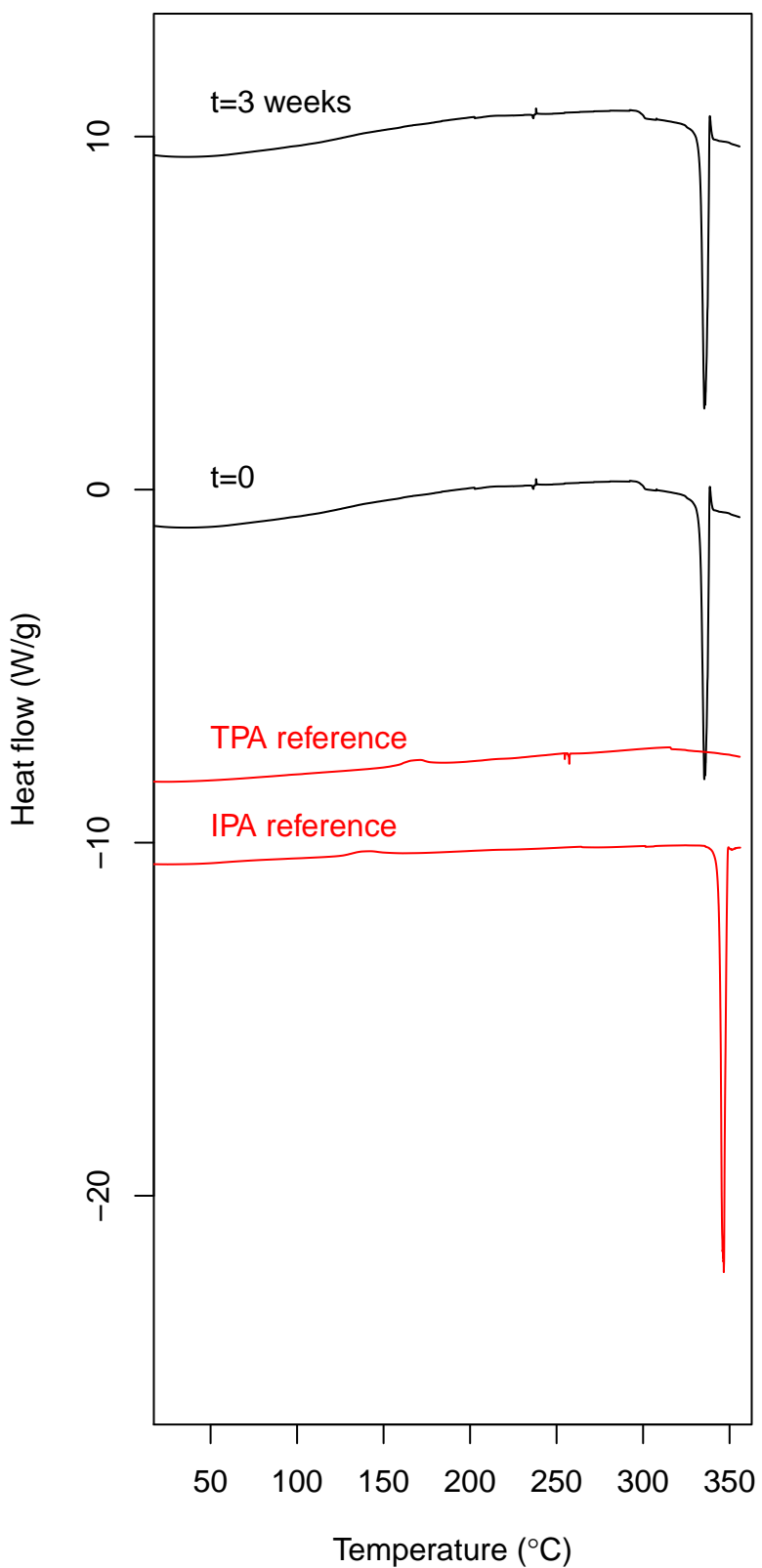

**b) Terephthalic + phthalic acid**

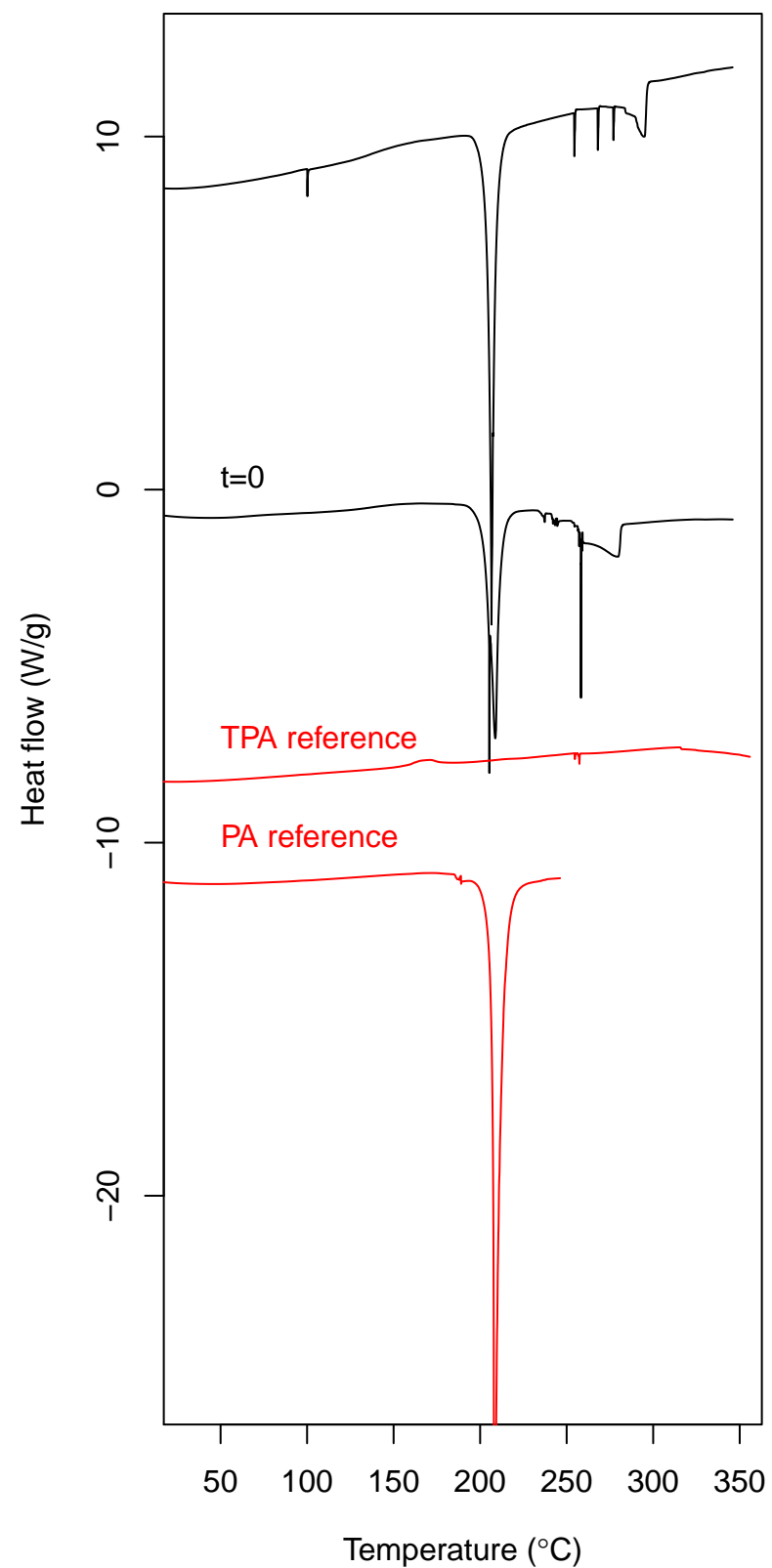

**c) Isophthalic + phthalic acid**

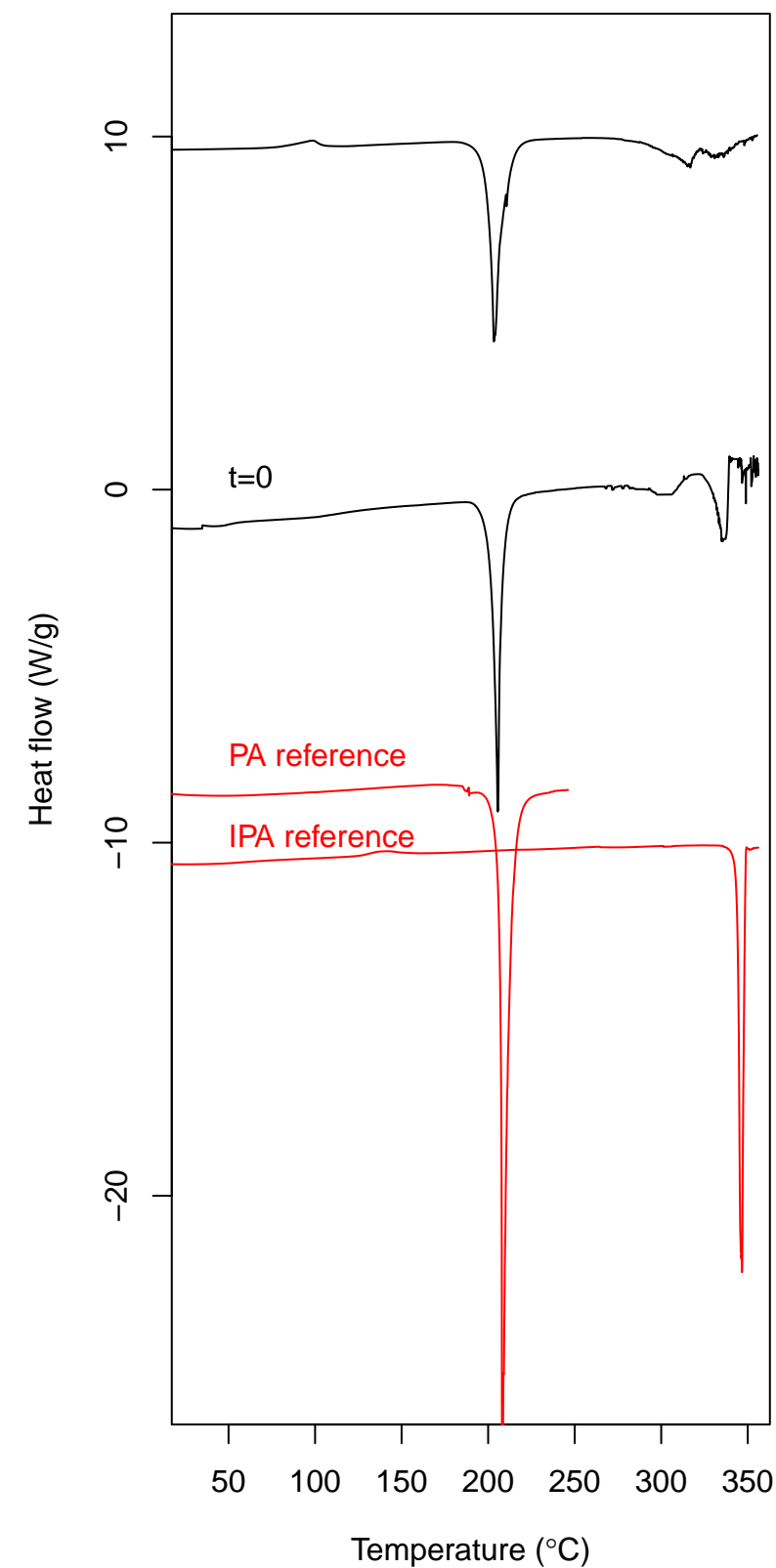

Supplement: Supplementary file 1 [file molecules-24-03990-s001.zip › SI_pack/Figure_5_DSC_two_components/Two_components_DSC.pdf]

**a) Terephthalic and isophthalic**

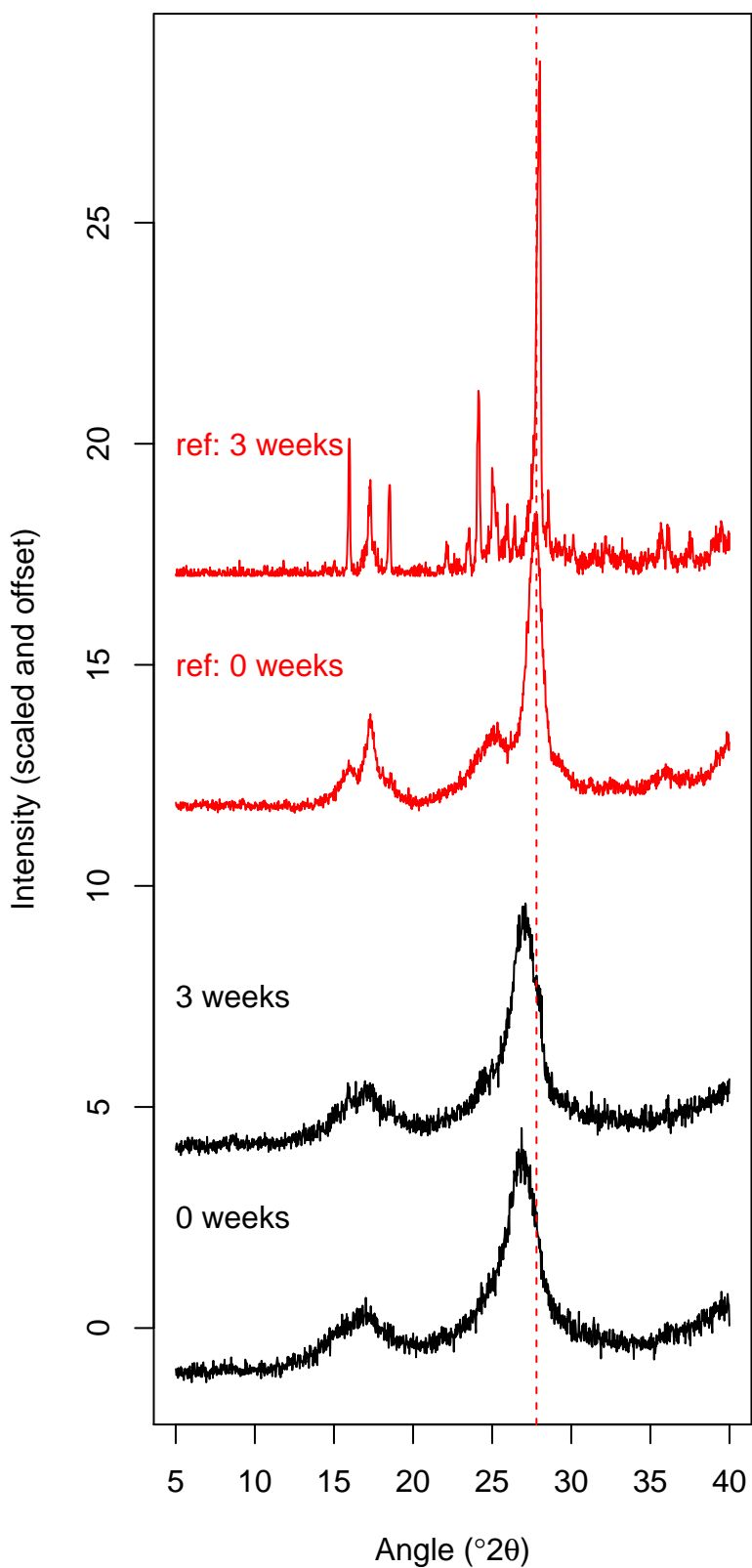

**b) Terephthalic and phthalic**

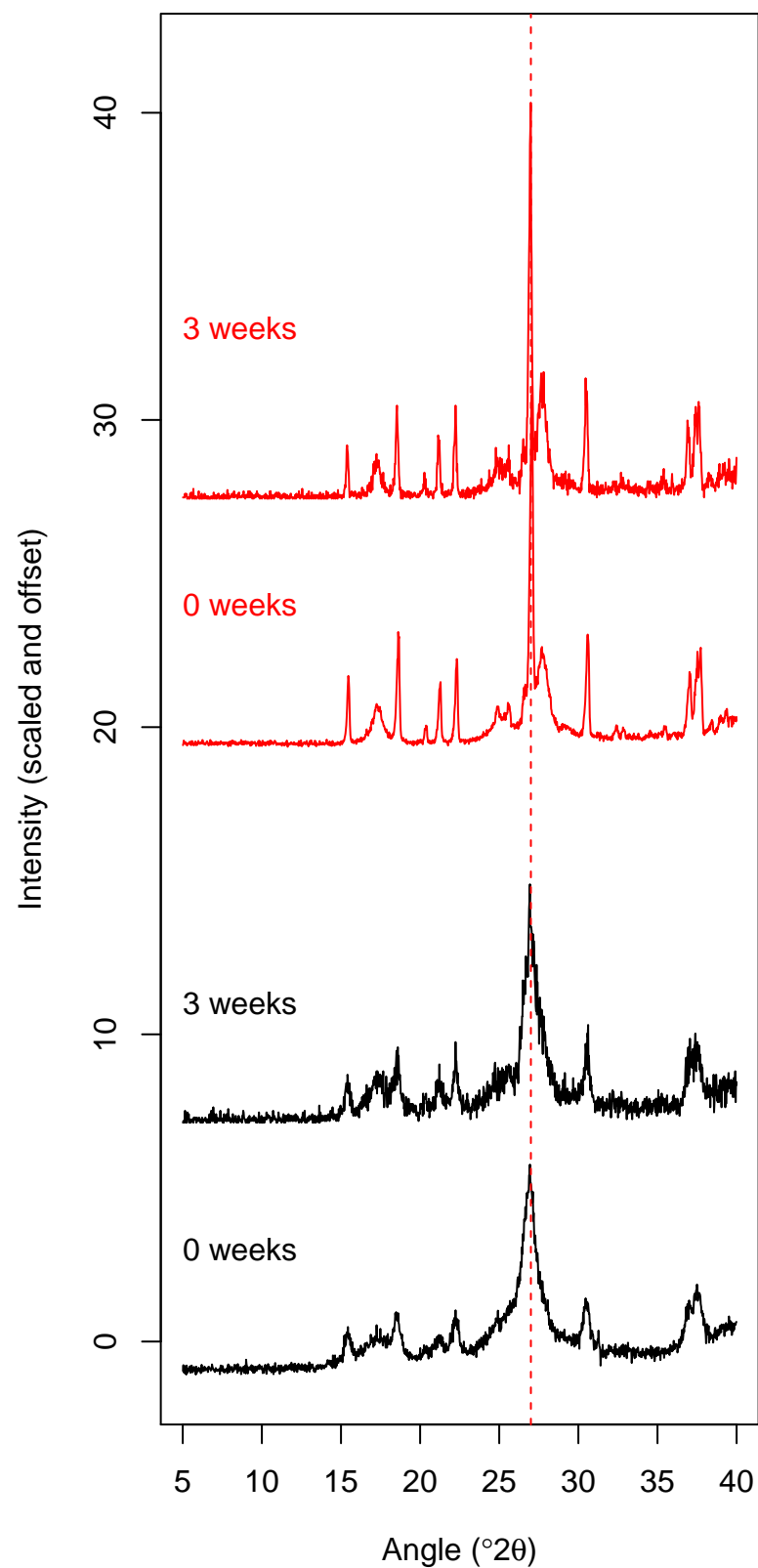

**c) Isophthalic and phthalic**

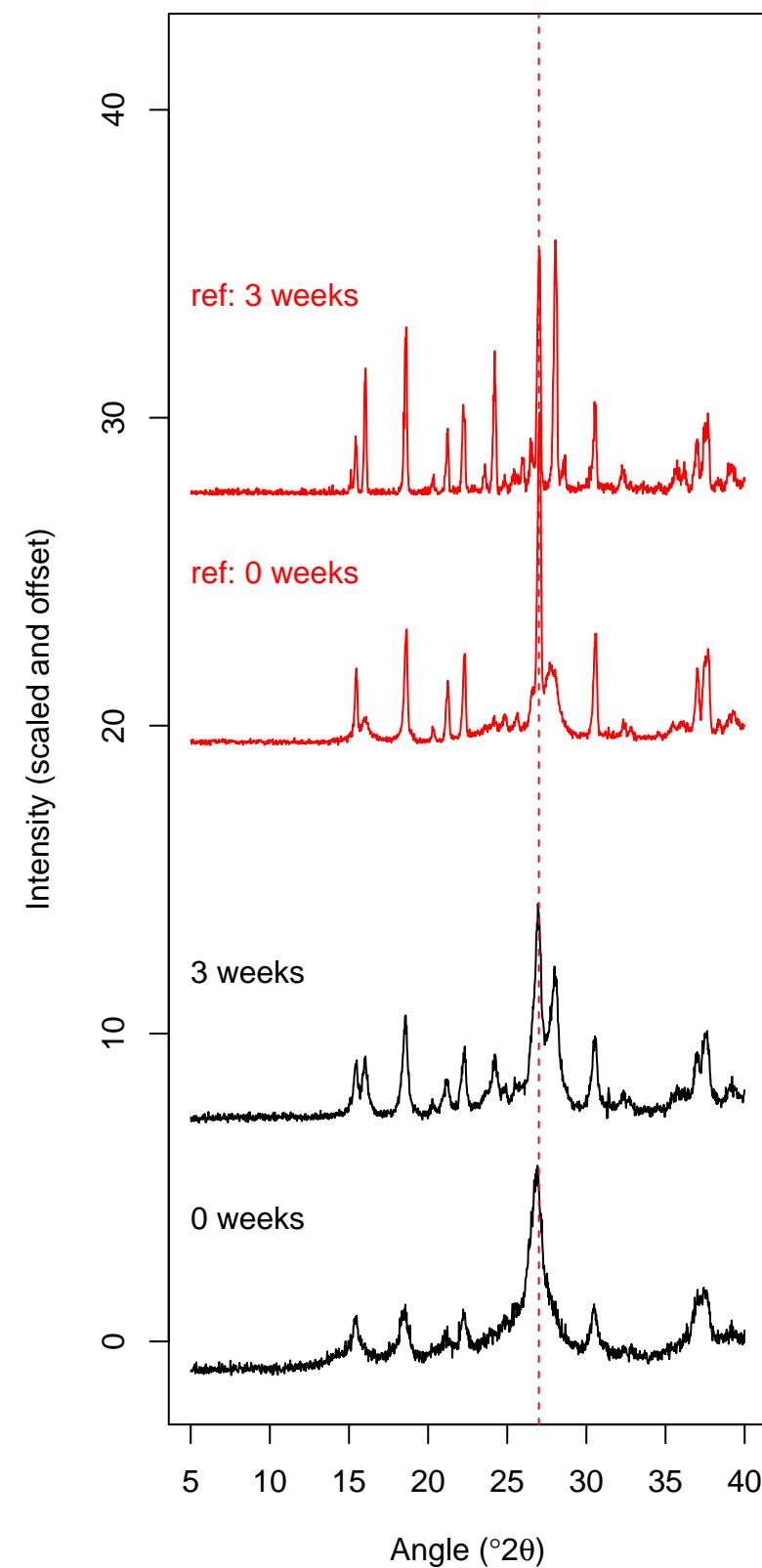

Supplement: Supplementary file 1 [file molecules-24-03990-s001.zip › SI_pack/Figure_6_XRPD_two_components/Figure_6_two_components_XRPD.pdf]

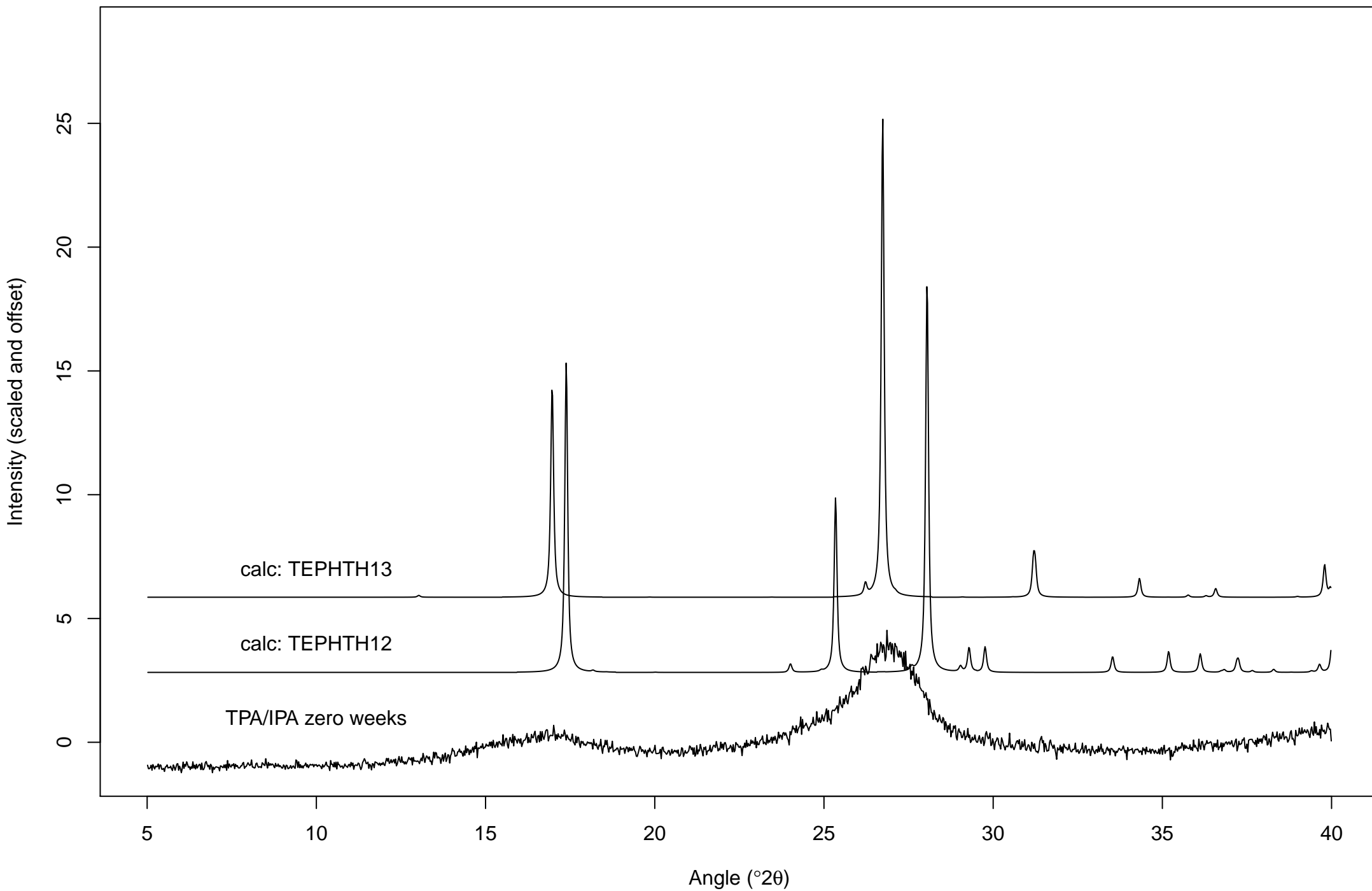

Supplement: Supplementary file 1 [file molecules-24-03990-s001.zip › SI_pack/Figure_7_Calculated_XRPD_patterns_TPA/Figure_7_TPA_IPA_vs_calculated_CCDC_patterns.pdf]

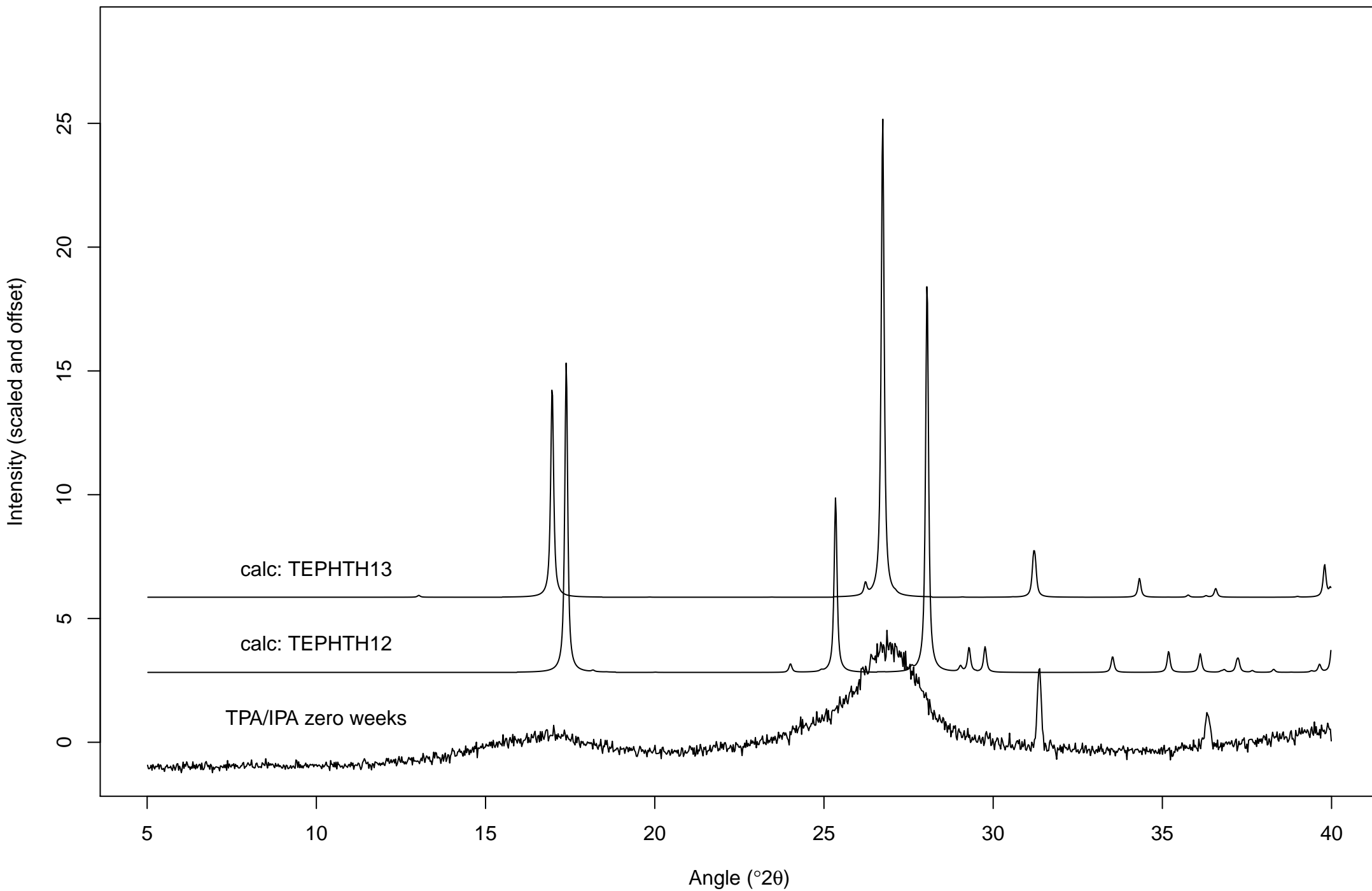

Supplement: Supplementary file 1 [file molecules-24-03990-s001.zip › SI_pack/Figure_7_Calculated_XRPD_patterns_TPA/TPA_IPA_vs_calculated_from_CCDC.pdf]
