# Supplementary material for: Investigation of Potential Amorphisation and Co-Amorphisation Behaviour of the Benzene Di-Carboxylic Acids upon Cryo-Milling
Source: Molecules. 2019 Nov 5;24(21):3990. doi: 10.3390/molecules24213990 (PMC6865180; doi:10.3390/molecules24213990)
Supplement: Supplementary file 1 [file molecules-24-03990-s001.zip › SI_pack/Figure_SI_ATR_FTIR_two_components/Data/PT/pt mixtures.pdf]

**halic and terephthalic acid CM together for 60mins (3**

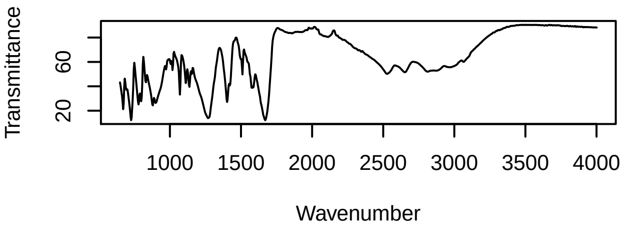


**alic and terephthalic acid CM together for 60mins (zer**

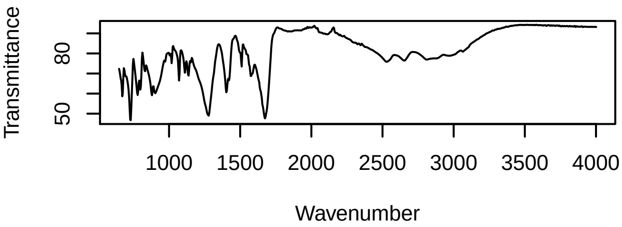


**alic and terephthalic acid CM separately for 60mins (**

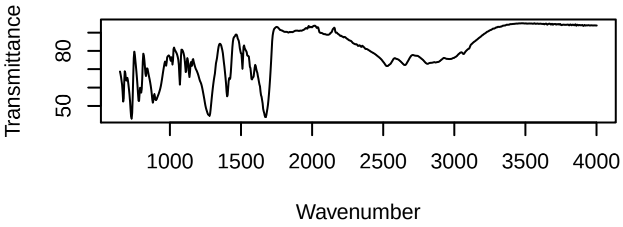


**lic and terephthalic acid CM separately for 60mins (ze**

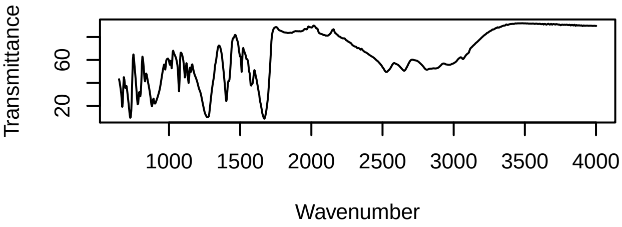


**Terepthalic acid CM 60mins**

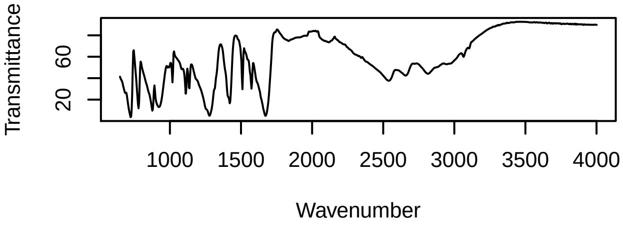


**Phthalic acid CM 60mins**

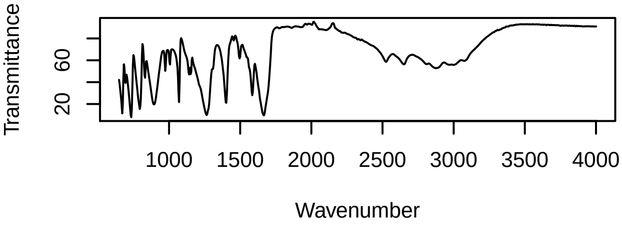

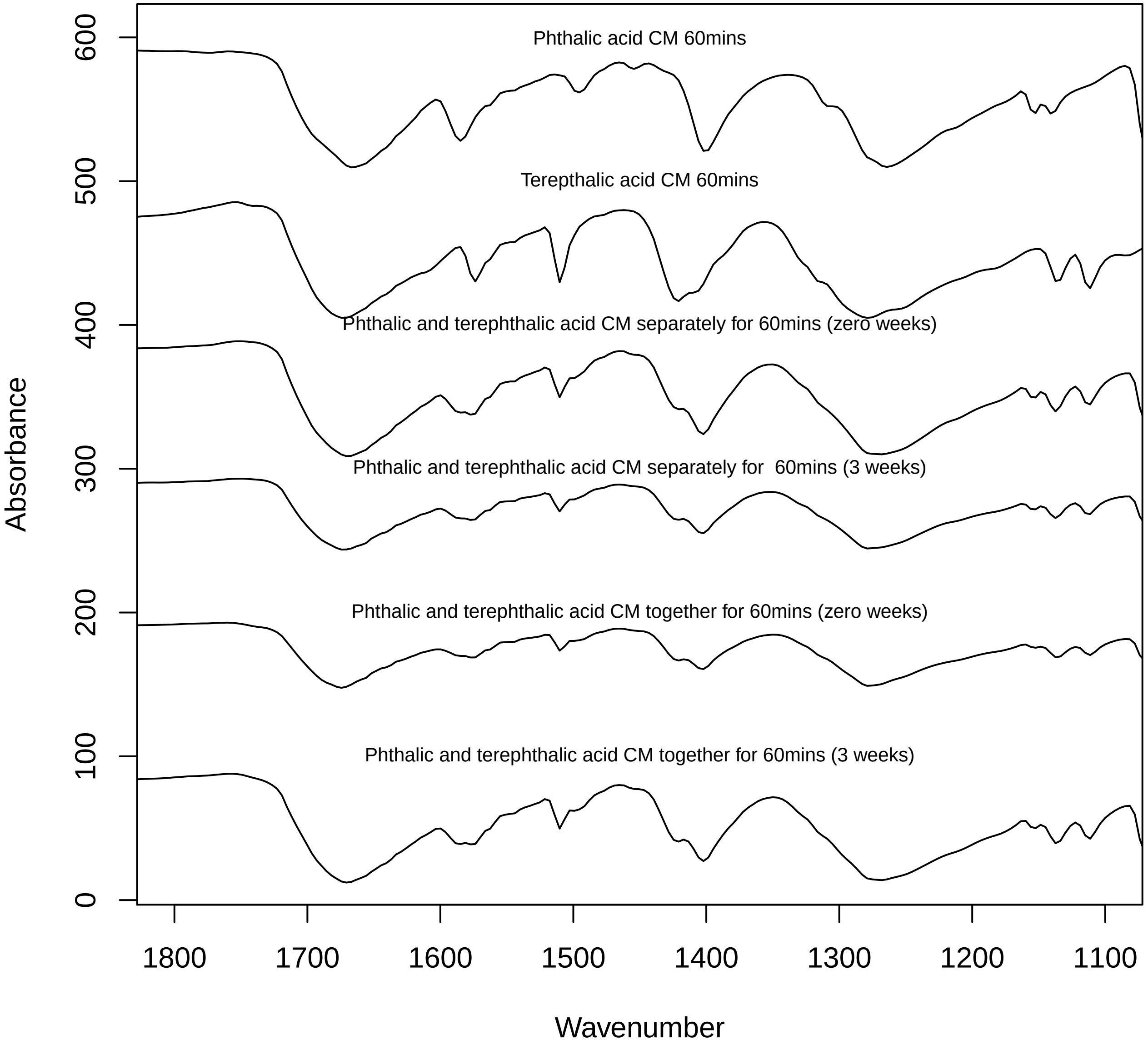

Phthalic acid CM 60mins
Terepthalic acid CM 60mins
Phthalic and terephthalic acid CM separately for 60mins (zero weeks)
Phthalic and terephthalic acid CM separately for 60mins (3 weeks)
Phthalic and terephthalic acid CM together for 60mins (zero weeks)
Phthalic and terephthalic acid CM together for 60mins (3 weeks)
Absorbance
0
100
200
300
400
500
600
Wavenumber
1800
1700
1600
1500
1400
1300
1200
1100
