# Supplementary material for: Investigation of Potential Amorphisation and Co-Amorphisation Behaviour of the Benzene Di-Carboxylic Acids upon Cryo-Milling
Source: Molecules. 2019 Nov 5;24(21):3990. doi: 10.3390/molecules24213990 (PMC6865180; doi:10.3390/molecules24213990)
Supplement: Supplementary file 1 [file molecules-24-03990-s001.zip › SI_pack/Figure_SI_ATR_FTIR_two_components/Data/PT/PT.pdf]

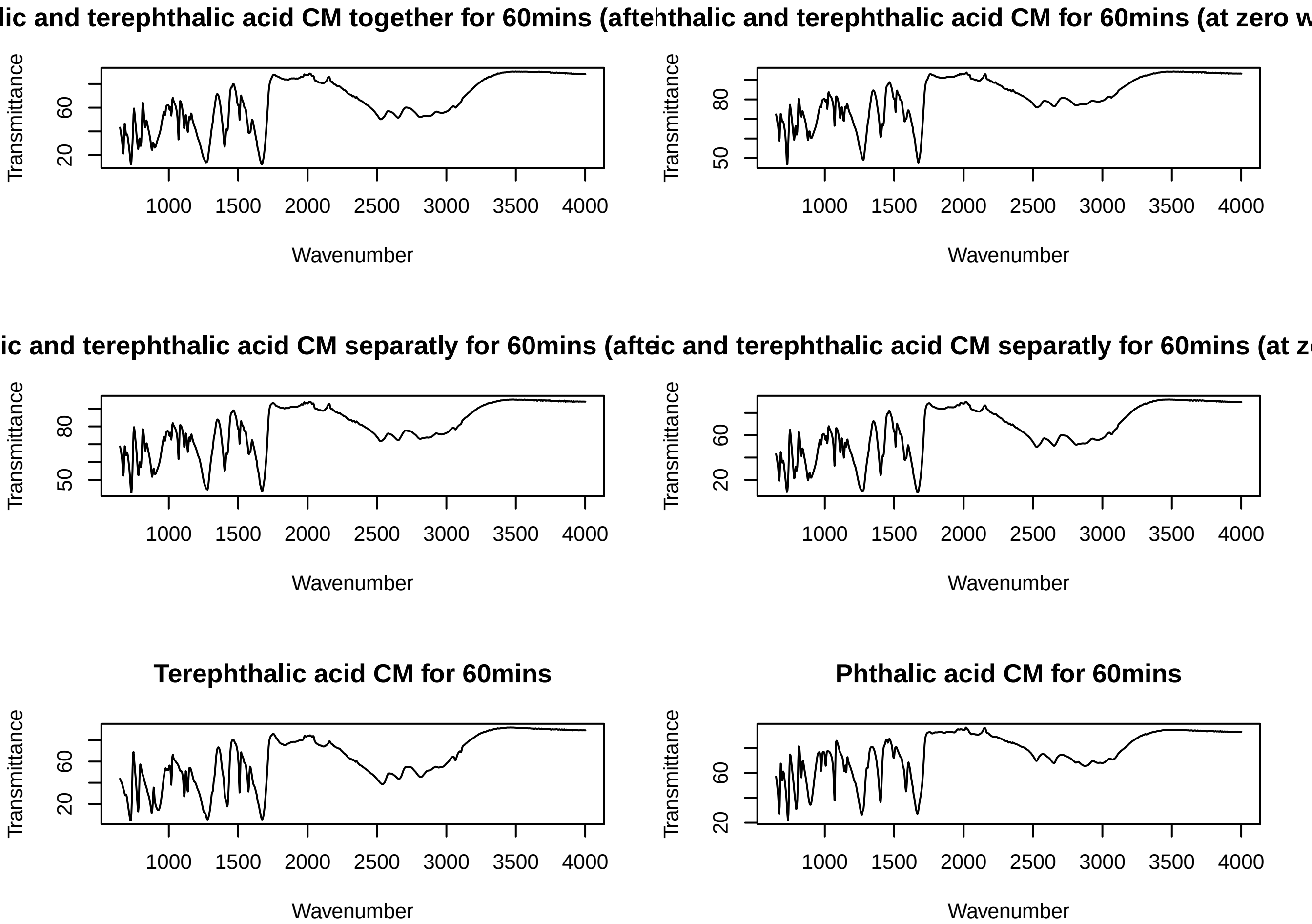
lic and terephthalic acid CM together for 60mins (afte
hthalic and terephthalic acid CM for 60mins (at zero w
ic and terephthalic acid CM separatly for 60mins (afte
ic and terephthalic acid CM separatly for 60mins (at ze
Terephthalic acid CM for 60mins
Phthalic acid CM for 60mins
Transmittance
Wavenumber
20
50
60
80
1000
1500
2000
2500
3000
3500
4000

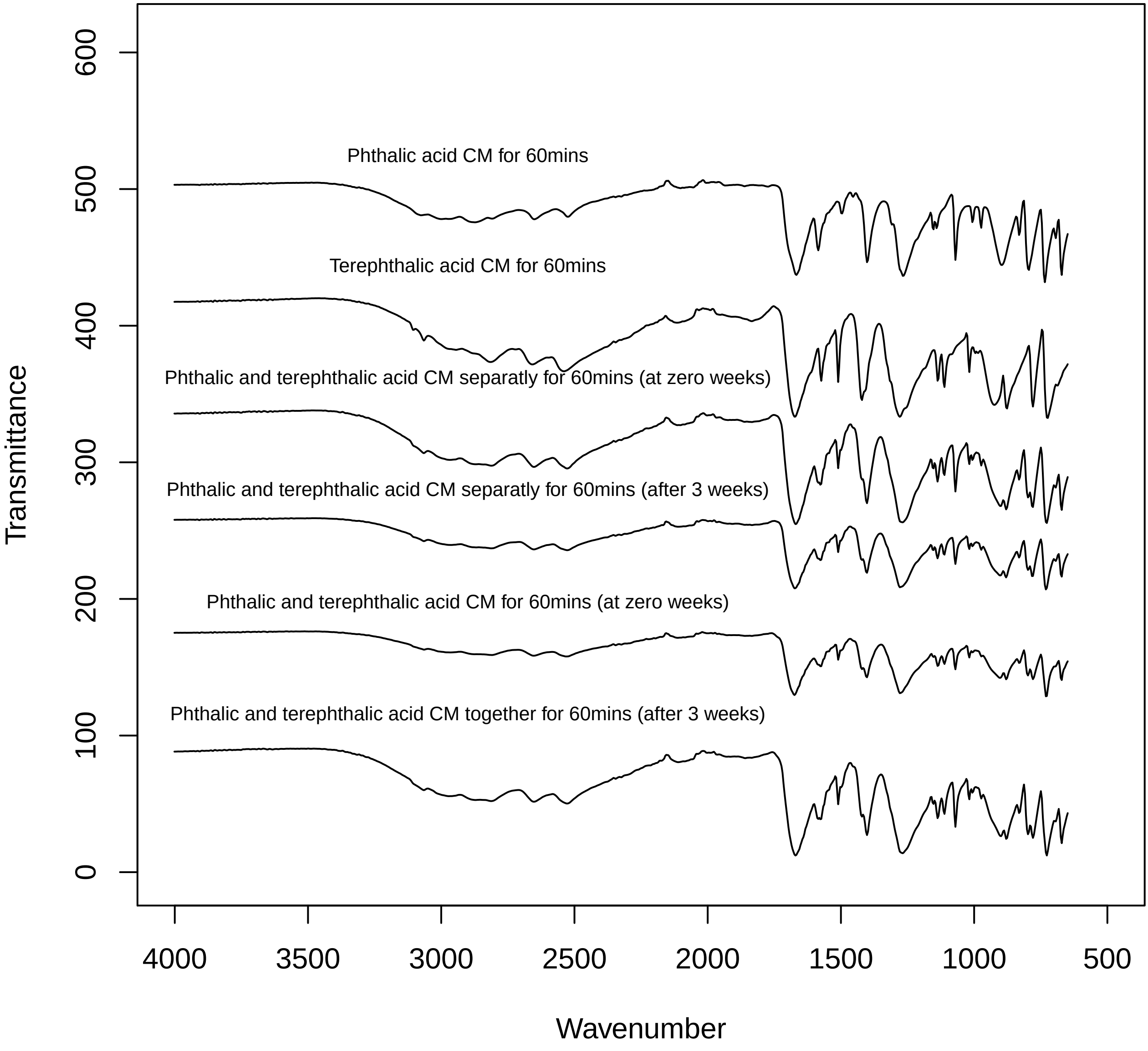
Phthalic acid CM for 60mins
Terephthalic acid CM for 60mins
Phthalic and terephthalic acid CM separatly for 60mins (at zero weeks)
Phthalic and terephthalic acid CM separatly for 60mins (after 3 weeks)
Phthalic and terephthalic acid CM for 60mins (at zero weeks)
Phthalic and terephthalic acid CM together for 60mins (after 3 weeks)
Transmittance
0
100
200
300
400
500
600
Wavenumber
4000
3500
3000
2500
2000
1500
1000
500
