# Supplementary material for: Investigation of Potential Amorphisation and Co-Amorphisation Behaviour of the Benzene Di-Carboxylic Acids upon Cryo-Milling
Source: Molecules. 2019 Nov 5;24(21):3990. doi: 10.3390/molecules24213990 (PMC6865180; doi:10.3390/molecules24213990)
Supplement: Supplementary file 1 [file molecules-24-03990-s001.zip › SI_pack/Figure_SI_ATR_FTIR_two_components/SI_ATR_FTIR_plots_two_components.pdf]

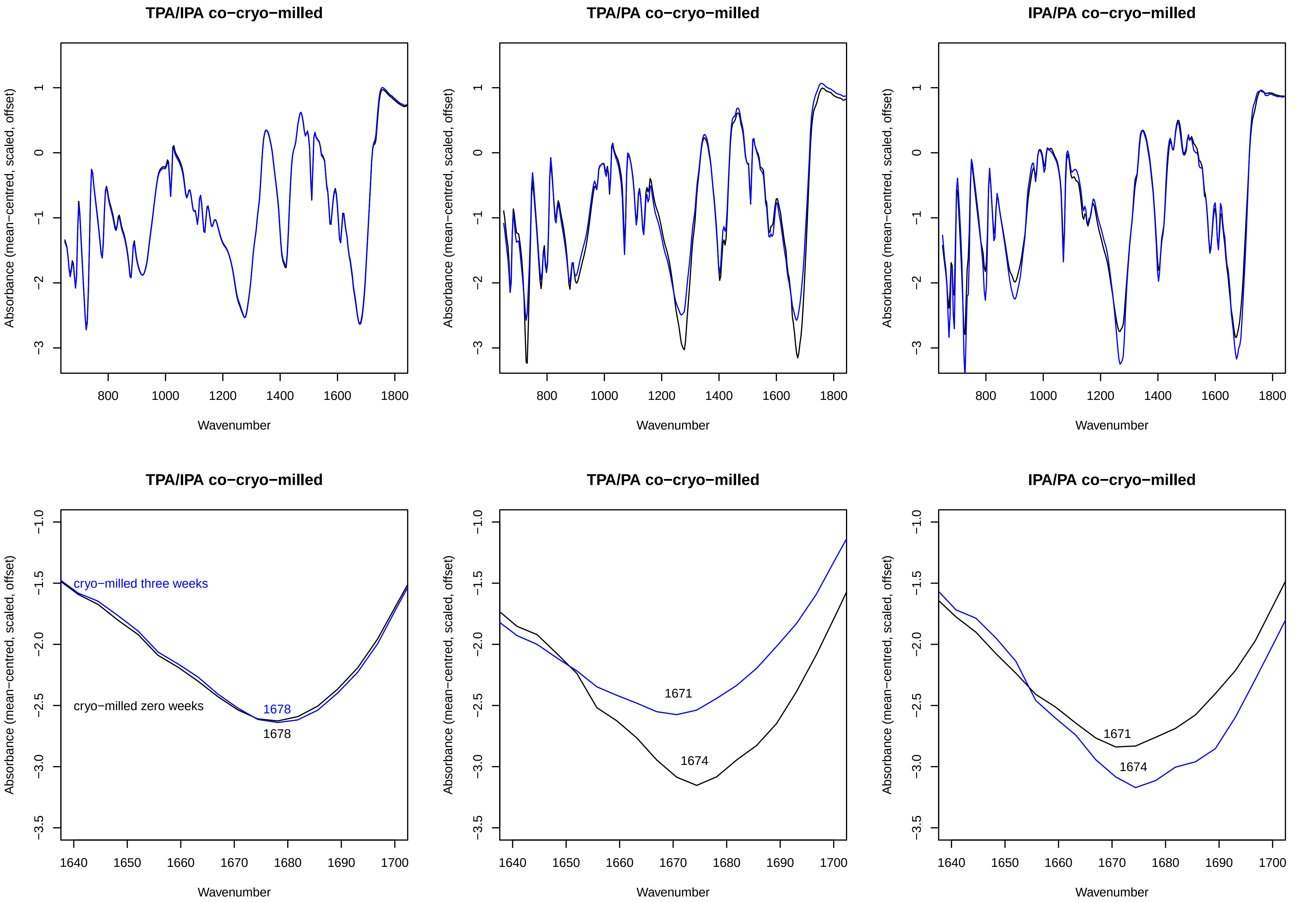

TPA/IPA co−cryo−milled
TPA/PA co−cryo−milled
IPA/PA co−cryo−milled
Absorbance (mean−centred, scaled, offset)
Wavenumber
cryo−milled three weeks
cryo−milled zero weeks
1678
1678
1671
1674
1671
1674

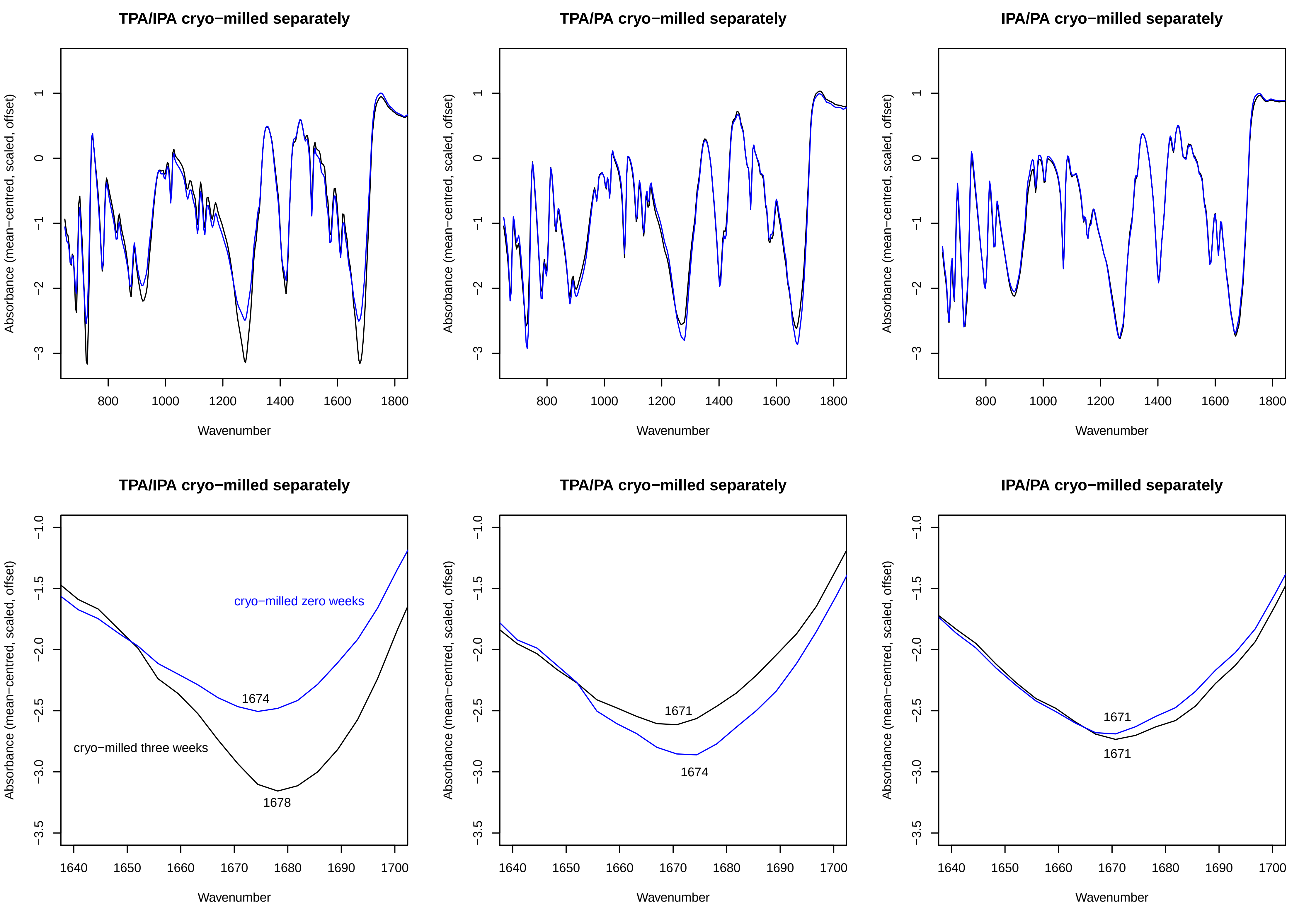
TPA/IPA cryo−milled separately
TPA/PA cryo−milled separately
IPA/PA cryo−milled separately
Absorbance (mean−centred, scaled, offset)
Wavenumber
800
1000
1200
1400
1600
1800
−3
−2
−1
0
1
TPA/IPA cryo−milled separately
TPA/PA cryo−milled separately
IPA/PA cryo−milled separately
−3.5
−3.0
−2.5
−2.0
−1.5
−1.0
1640
1650
1660
1670
1680
1690
1700
cryo−milled zero weeks
cryo−milled three weeks
1674
1678
1671
1674
1671
1671

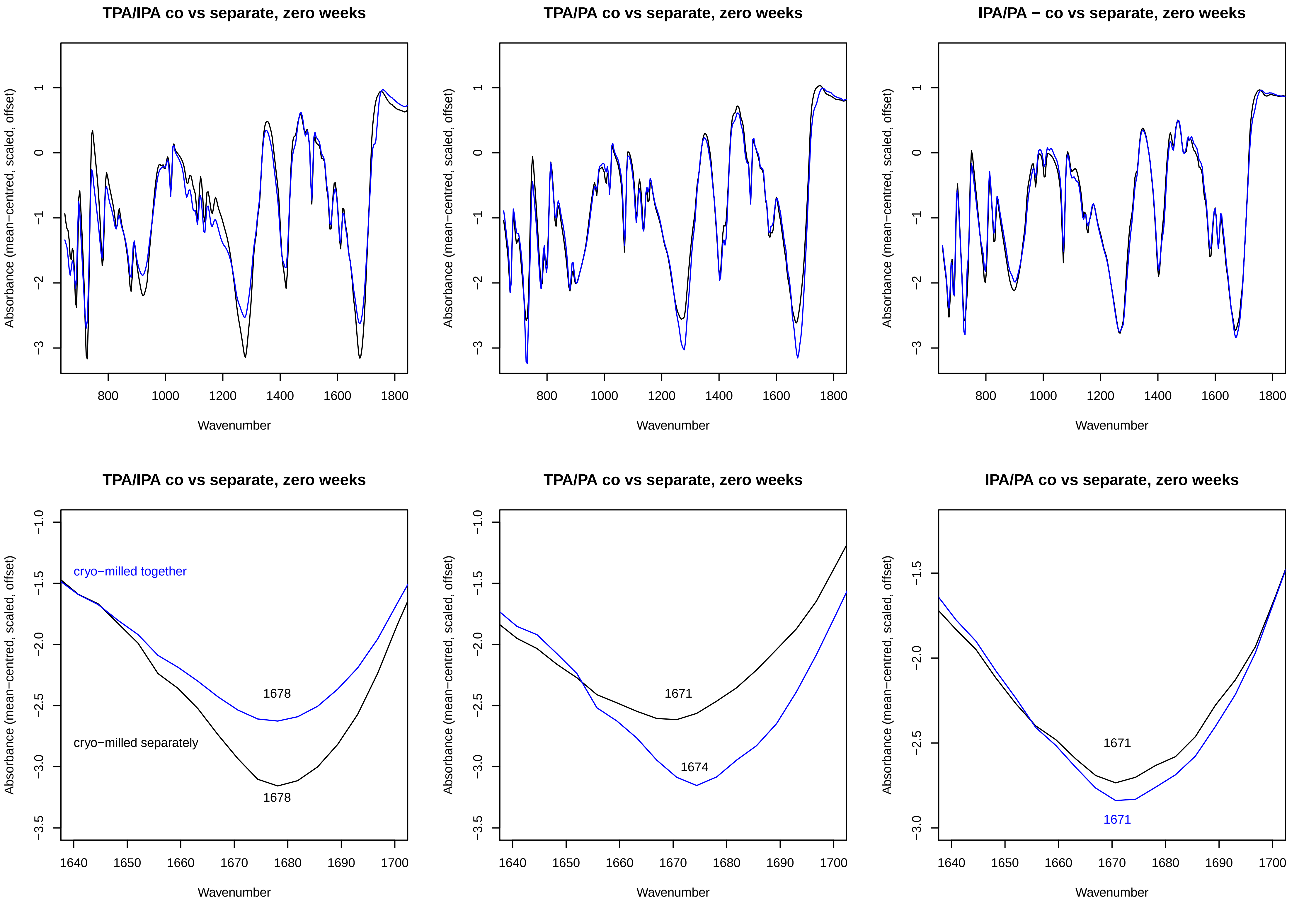
TPA/IPA co vs separate, zero weeks
TPA/PA co vs separate, zero weeks
IPA/PA − co vs separate, zero weeks
Absorbance (mean−centred, scaled, offset)
Wavenumber
TPA/IPA co vs separate, zero weeks
TPA/PA co vs separate, zero weeks
IPA/PA co vs separate, zero weeks
cryo−milled together
cryo−milled separately
1678
1678
1671
1674
1671
1671

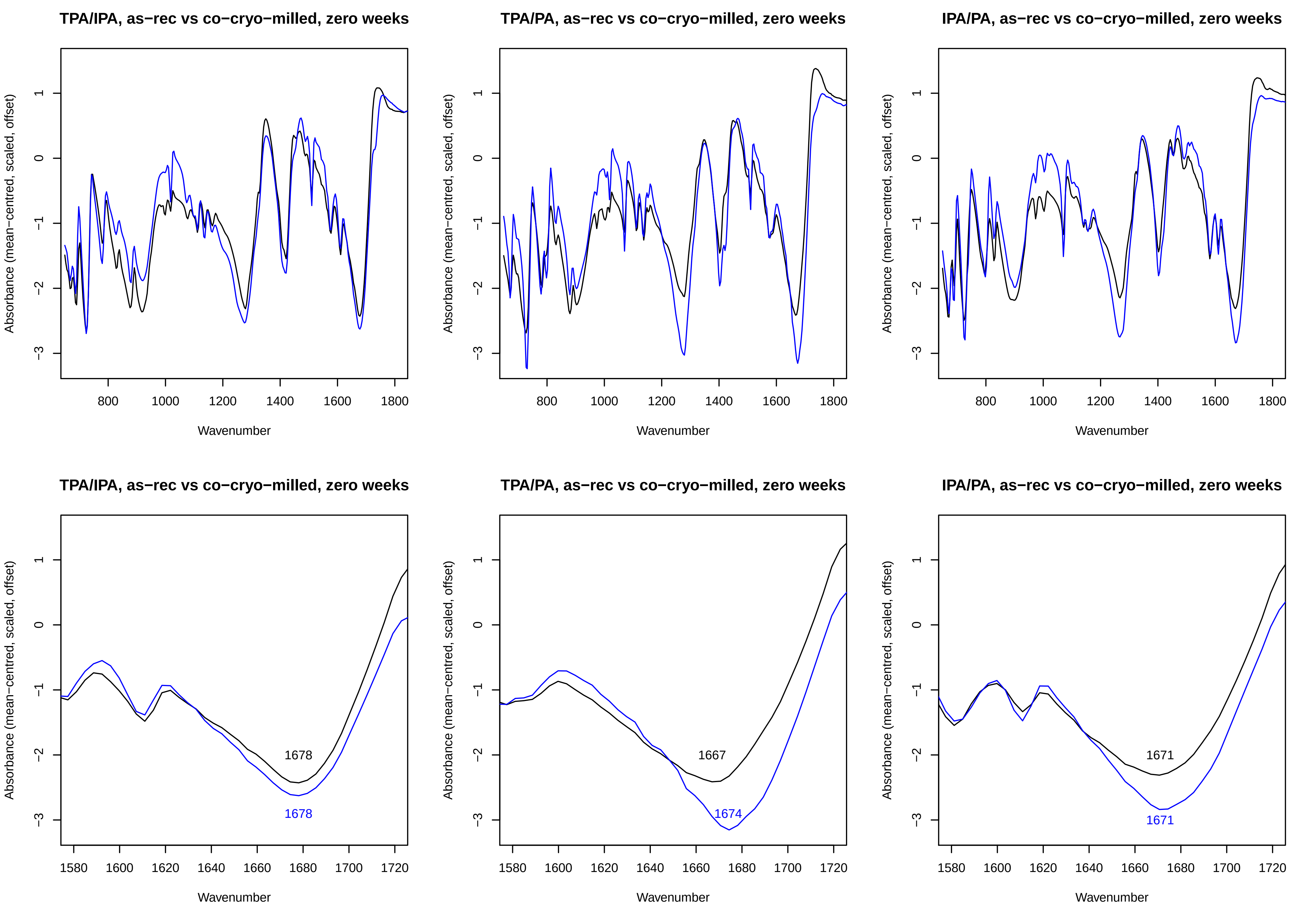
TPA/IPA, as−rec vs co−cryo−milled, zero weeks
TPA/PA, as−rec vs co−cryo−milled, zero weeks
IPA/PA, as−rec vs co−cryo−milled, zero weeks
Absorbance (mean−centred, scaled, offset)
Wavenumber
800
1000
1200
1400
1600
1800
1580
1600
1620
1640
1660
1680
1700
1720
1
0
−1
−2
−3
1678
1678
1667
1674
1671
1671
