# Supplementary material for: Investigation of Potential Amorphisation and Co-Amorphisation Behaviour of the Benzene Di-Carboxylic Acids upon Cryo-Milling
Source: Molecules. 2019 Nov 5;24(21):3990. doi: 10.3390/molecules24213990 (PMC6865180; doi:10.3390/molecules24213990)

TPA

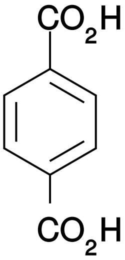

IPA

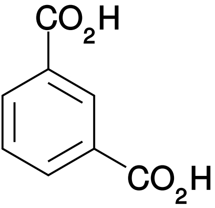

PA

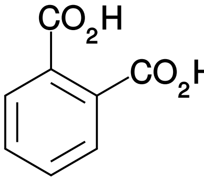

Supplement: Supplementary file 1 [file molecules-24-03990-s001.zip › SI_pack/Fig_1_TPA_IPA_PA_chemical_structures.pdf]
