# Supplementary material for: Investigation of Potential Amorphisation and Co-Amorphisation Behaviour of the Benzene Di-Carboxylic Acids upon Cryo-Milling
Source: Molecules. 2019 Nov 5;24(21):3990. doi: 10.3390/molecules24213990 (PMC6865180; doi:10.3390/molecules24213990)
Supplement: Supplementary file 1 [file molecules-24-03990-s001.zip › SI_pack/picture.pdf]

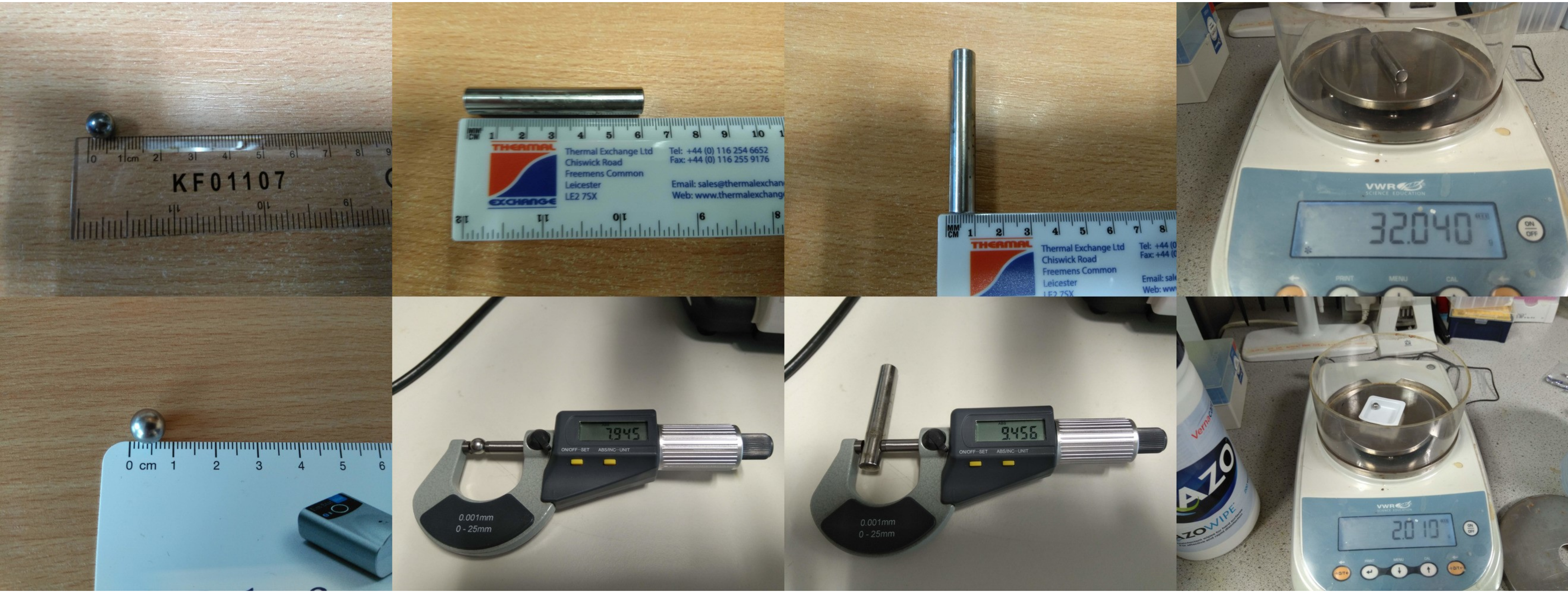
KF01107
Thermal Exchange Ltd
Chiswick Road
Freemens Common
Leicester
LE2 7SX
Tel: +44 (0) 116 254 6652
Fax: +44 (0) 116 255 9176
32.040
7.945
9.456
ON/OFF - SET
ABS/INC - UNIT
0.001mm
0 - 25mm
2.010
