# Supplementary material for: Investigation of Potential Amorphisation and Co-Amorphisation Behaviour of the Benzene Di-Carboxylic Acids upon Cryo-Milling
Source: Molecules. 2019 Nov 5;24(21):3990. doi: 10.3390/molecules24213990 (PMC6865180; doi:10.3390/molecules24213990)
Supplement: Supplementary file 1 [file molecules-24-03990-s001.zip › SI_pack/pngs/pg_0003.pdf]

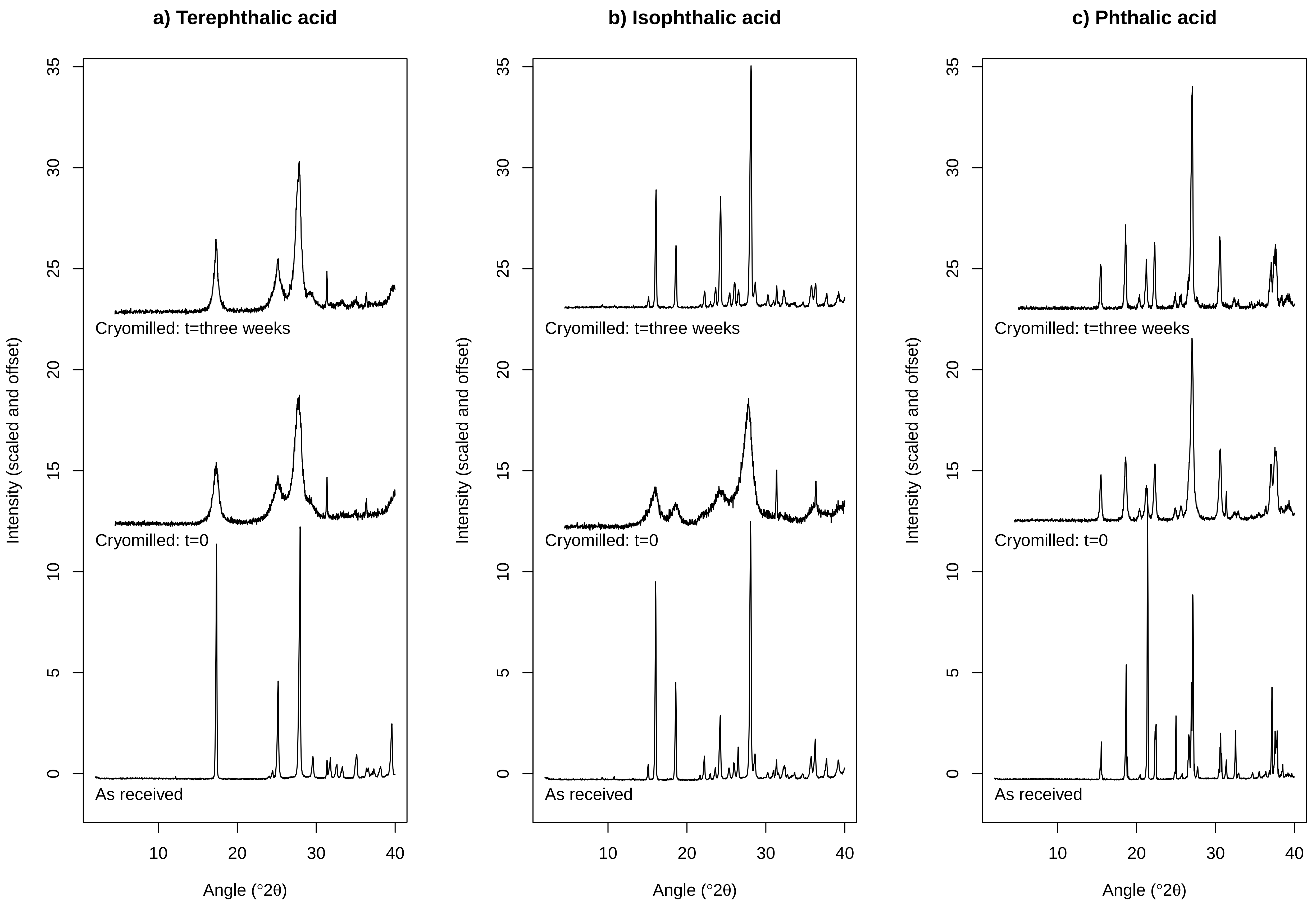

a) Terephthalic acid
b) Isophthalic acid
c) Phthalic acid
Intensity (scaled and offset)
Angle (°2θ)
Cryomilled: t=three weeks
Cryomilled: t=0
As received
0
5
10
15
20
25
30
35
10
20
30
40
