# Supplementary material for: Investigation of Potential Amorphisation and Co-Amorphisation Behaviour of the Benzene Di-Carboxylic Acids upon Cryo-Milling
Source: Molecules. 2019 Nov 5;24(21):3990. doi: 10.3390/molecules24213990 (PMC6865180; doi:10.3390/molecules24213990)
Supplement: Supplementary file 1 [file molecules-24-03990-s001.zip › SI_pack/pngs/pg_0004.pdf]

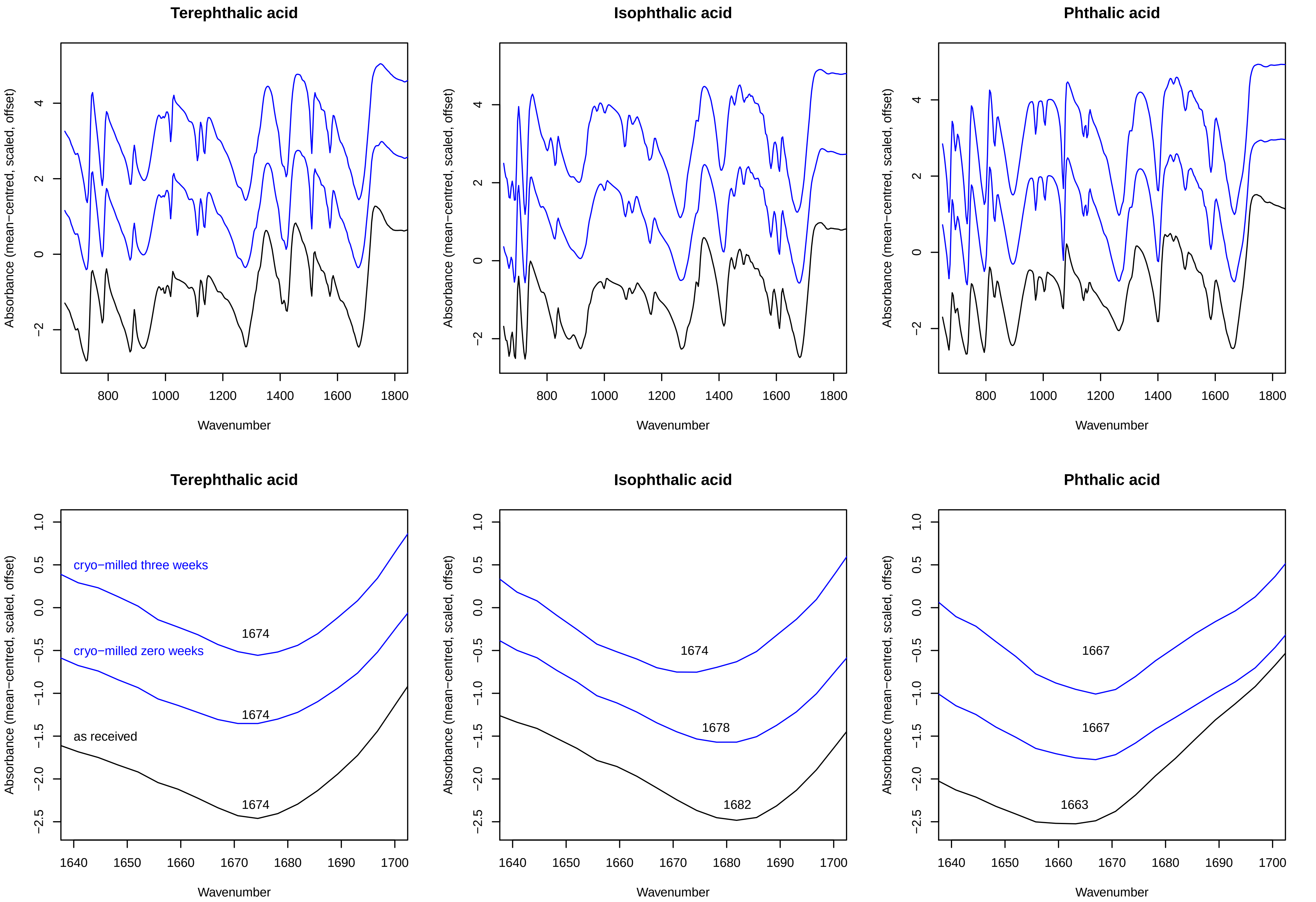
Terephthalic acid
Isophthalic acid
Phthalic acid
Absorbance (mean−centred, scaled, offset)
Wavenumber
−2
0
2
4
800
1000
1200
1400
1600
1800
Terephthalic acid
Isophthalic acid
Phthalic acid
−2.5
−2.0
−1.5
−1.0
−0.5
0.0
0.5
1.0
1640
1650
1660
1670
1680
1690
1700
cryo−milled three weeks
cryo−milled zero weeks
as received
1674
1674
1674
1674
1678
1682
1667
1667
1663
