# Supplementary material for: Investigation of Potential Amorphisation and Co-Amorphisation Behaviour of the Benzene Di-Carboxylic Acids upon Cryo-Milling
Source: Molecules. 2019 Nov 5;24(21):3990. doi: 10.3390/molecules24213990 (PMC6865180; doi:10.3390/molecules24213990)
Supplement: Supplementary file 1 [file molecules-24-03990-s001.zip › SI_pack/pngs/pg_0005.pdf]

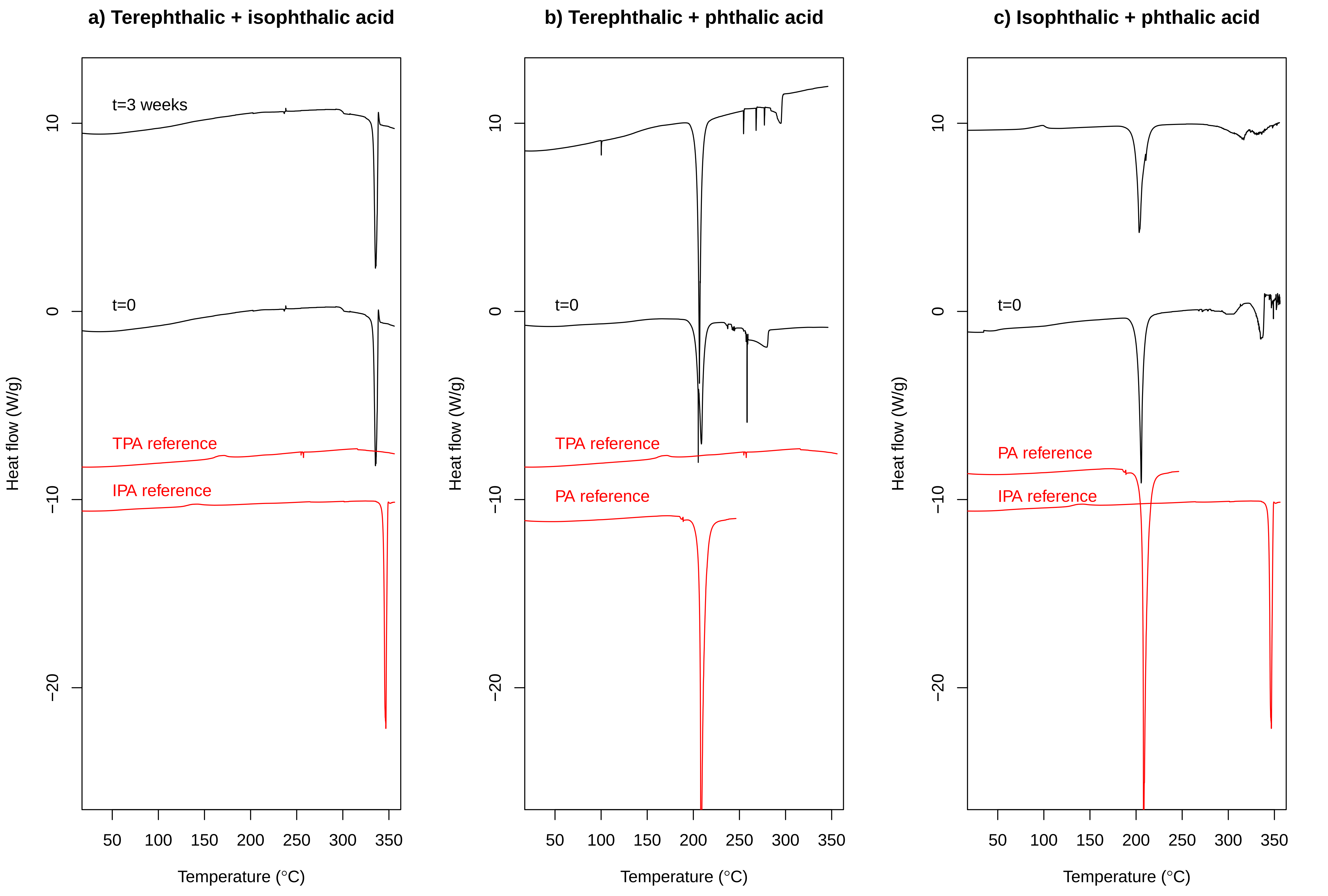

a) Terephthalic + isophthalic acid
t=3 weeks
t=0
TPA reference
IPA reference
b) Terephthalic + phthalic acid
t=0
TPA reference
PA reference
c) Isophthalic + phthalic acid
t=0
PA reference
IPA reference
Heat flow (W/g)
10
0
−10
−20
Temperature (°C)
50
100
150
200
250
300
350
