# Supplementary material for: Investigation of Potential Amorphisation and Co-Amorphisation Behaviour of the Benzene Di-Carboxylic Acids upon Cryo-Milling
Source: Molecules. 2019 Nov 5;24(21):3990. doi: 10.3390/molecules24213990 (PMC6865180; doi:10.3390/molecules24213990)
Supplement: Supplementary file 1 [file molecules-24-03990-s001.zip › SI_pack/pngs/pg_0006.pdf]

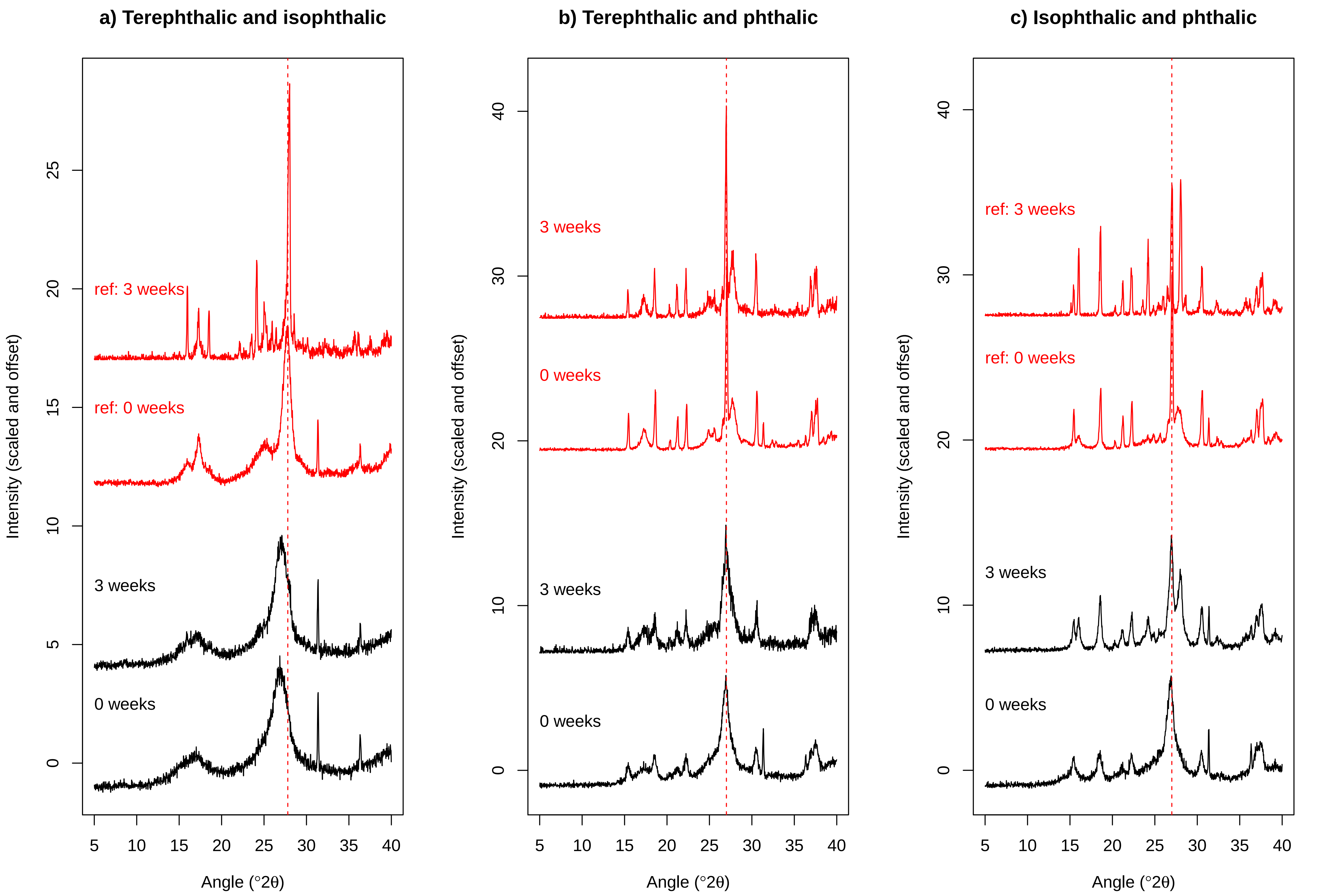
a) Terephthalic and isophthalic
b) Terephthalic and phthalic
c) Isophthalic and phthalic
Intensity (scaled and offset)
Angle (°2θ)
ref: 3 weeks
ref: 0 weeks
3 weeks
0 weeks
5
10
15
20
25
30
35
40
